# Comparison of different exercise modalities on fatigue and muscular fitness in patients with multiple sclerosis: a systematic review with network, and dose–response meta-analyses

**DOI:** 10.3389/fneur.2024.1494368

**Published:** 2024-11-26

**Authors:** Xi-Nuan Zhang, Zhi-De Liang, Ming-Da Li

**Affiliations:** ^1^School of Sports Training, Chengdu Sport University, Chengdu, Sichuan, China; ^2^Department of Physical Education, College of Physical Education, Qingdao University, Qingdao, China

**Keywords:** multiple sclerosis, fatigue, muscular fitness, exercise interventions, meta-analysis

## Abstract

**Background:**

Fatigue and muscular fitness are closely related to the quality of life in patients with multiple sclerosis (MS). However, the optimal exercise dosage to improve these outcomes remains unclear.

**Objective:**

We evaluated the effects of different exercise modalities and dosages on fatigue levels and muscular fitness in patients with MS.

**Methods:**

A systematic search was conducted across five electronic databases, including randomized controlled trials involving exercise interventions for patients with MS. The data covered literature from the establishment of each database up to August 2024. Two independent reviewers assessed the quality of the studies. Network and dose–response meta-analyses were performed using a random-effects model to evaluate the impact of exercise.

**Results:**

A total of 84 papers were included, involving 3,786 participants. The network meta-analysis revealed that mind–body exercise (MBE) had the most significant effect on reducing fatigue (SMD = −0.94; 95% CrI: −1.3 to −0.6), followed by resistance training (RT) (SMD = −0.86; 95% CrI: −1.2 to −0.58), combined exercise (COM) (SMD = −0.70; 95% CrI: −1.2 to −0.22), and high-intensity interval training (HIIT) (SMD = −0.50; 95% CrI: −1.1 to 0.14). Additionally, HIIT were found to be the most effective in improving muscular fitness (SMD = −0.86; 95% CrI: −1.5 to −0.27), followed by COM (SMD = −0.81; 95% CrI: −1.2 to −0.41), MBE (SMD = −0.64; 95% CrI: −1.1 to −0.16), and RT (SMD = −0.62; 95% CrI: −0.89 to −0.16). Moreover, a dose as low as 240 METs-min/week was sufficient to improve fatigue, while a dose of 430 METs-min/week was required to enhance muscular fitness. The optimal dose for reducing fatigue was 650 METs-min/week, and the best dose for improving muscular fitness was 530 METs-min/week.

**Conclusion:**

Exercise is an effective method for improving fatigue and muscular fitness in patients with MS. While MBE and COM rank relatively higher compared to other exercise modalities. The optimal exercise dosage for reducing fatigue and improving muscular fitness ranges between approximately 530 to 860 MET-minutes per week.

**Systematic review registration:**

https://www.crd.york.ac.uk/PROSPERO/, CRD42024577643.

## 1 Introduction

Multiple sclerosis (MS) is a chronic, immune-mediated disease of the central nervous system (CNS), with neurodegenerative processes being an important aspect of the disease (1). It is affecting approximately 2.8 million people worldwide and has been found to one of the most common causes of non-traumatic disability among young adults (aged 18–40 years) (2, 3).

The symptoms of the disease and associated dysfunctions can significantly affect patients’ daily quality of life (e.g., fatigue and mobility impairment), with substantial impacts on social, economic, and personal well-being (4). MS is typically treated with disease-modifying drugs, which control inflammation but do not address neurodegenerative processes, leading to residual symptoms and dysfunction (1). Among nonpharmacologic management, exercise can be a beneficial rehabilitation approach for MS, particularly, reducing fatigue and addressing mobility problems have received particular attention as ways to improve overall quality of life (1, 5, 6).

Exercise was also mentioned in some national guidelines. For example, the World Health Organization (WHO) mentioned in its 2020 guidelines that there is high-certainty evidence indicating that physical activity can improve function in patients with multiple sclerosis (7). In the UK, the National Institute for Health and Care Excellence (NICE) published clinical guidelines in 2022 advised people that aerobic, resistive and balance exercises, including yoga and pilates, may be helpful in treating MS-related fatigue, and for those with mobility problems, consider supervised exercise programs featuring moderate progressive resistance training (RT) and aerobic exercise (8). It can be observed that these guidelines generally encourage MS patients to engage in exercise, but few provide clear and consistent recommendations regarding dose and exercise modality. The MS Society UK also advises patients to be mindful of the impact of too much exercise on fatigue management (9). This may be because it is difficult for guideline authors to reach consistent conclusions through previous pairwise meta-analyses (10). Given the characteristics of exercise interventions, network meta-analyses (NMAs) can simultaneously compare multiple exercise modalities, thereby providing more direct and indirect comparisons of results. Additionally, regarding the classification of exercise, previous studies have often analyzed yoga, tai chi, and pilates as forms of resistive exercise, which, at this stage, appears to be inappropriate and may affect the final conclusions (11).

NMAs have already been used to study the effects of various exercise interventions on MS patients. In a previous study, it was shown that exercise could be used as a strategy for fatigue management, with RT and combined exercise significantly reducing patients’ fatigue levels (5). Another study indicated that RT and combined exercise appear to be the most effective exercises for improving muscular fitness (6). These studies categorized combined exercise to include aerobic exercise combined with RT and other auxiliary exercises; however, it has long been recognized that different exercise modalities yield different outcomes. Such broad categorization may impact the accuracy of conclusions regarding exercise (12). Therefore, precise definitions and classifications seem necessary. Moreover, current studies have also shown that attention to exercise intensity is still insufficient (5, 6). So, the dose–response relationship is critical to explore, as flagged by the WHO in 2020 (7). At the same time, the dose–response relationship has already been widely applied in the field of exercise interventions (12). However, to date, research on exercise dose for MS patients is still underexplored. These studies collectively indicate that there is a need to further conduct systematic research on exercise modality and dose for MS patients to determine the minimum effective dose, the optimal dose, and the maximum safe threshold of different exercise modalities, thereby providing more precise guidance for clinical practice in MS.

As a result, we performed a network meta-analysis (NMA) and dose–response network meta-analysis using a Bayesian model model-based NMA to evaluate the impact of various exercise modalities and dosages on fatigue in patients with MS. Given the complex factors that contribute to mobility issues in MS patients, we chose “muscular fitness” as a key outcome to investigate the effects of different exercise interventions on this aspect of health (6, 13). Additionally, the included studies assessed outcomes that were primarily related to muscle strength and power, with commonly used tests including the sit-to-stand test, curl-up test, maximal voluntary contraction, and plank-hold test. Furthermore, we analyzed how a range of variables could potentially influence the effectiveness of these exercise interventions.

## 2 Methods

### 2.1 Protocol and registration

This network meta-analysis was conducted in accordance with the Preferred Reporting Items for Systematic Reviews and Meta-Analyses extension statement for network meta-analyses statement (PRISMA-NMA) guidelines (14), and in accordance with the Cochrane Handbook for Systematic Reviews of Interventions. The study protocol was registered in PROSPERO (registration number: CRD42024577643).

### 2.2 Search strategy and study selection

Systematic searches were conducted in MEDLINE (PubMed), Embase, Web of Science, Scopus, SPORTDiscus and Cochrane databases to identify studies evaluating the effects of various exercise interventions on fatigue and muscular fitness in individuals with multiple sclerosis up until August 2024. During that time frame, all articles from around the world in any language were incorporated. Manual searches of previously published meta-analyses and reviews were carried out to uncover further sources. The search strategy employed the following medical subject terms or keywords: “exercise,” “physical activity,” “Multiple sclerosis,” “muscle,” and “fatigue.” Detailed search strategies are provided in the Electronic Supplementary Material (ESM).

Two independent reviewers (Xn-Z and Zd-L) conducted an initial screening of titles and abstracts, followed by a full-text review based on the established inclusion criteria. Any discrepancies were resolved through consultation with a third researcher (Md-L). The Endnote X9 software (Thompson ISI Research Soft, Philadelphia, PA, USA) was employed to organize and manage these records.

### 2.3 Eligibility criteria

Inclusion criteria for studies were determined based on the following guidelines: (a) Patients with multiple sclerosis; (b) Studies that compared various exercise modalities or intensities, as well as those that compared exercise interventions against non-exercise control groups, including usual care or waitlist conditions, were considered. Exercise was described as a deliberate, structured, and repeatable physical activity (15). We categorized exercise into five distinct types, with specific definitions provided in Table 1; (c) The outcomes evaluated encompassed changes in various standardized scales commonly used to assess fatigue in MS patients, alongside test results related to muscular fitness. Specific outcomes and further details are available in the ESM; (d) The study type was a randomized controlled trial (RCT).

**Table 1:** Definitions of exercise training interventions.

Modality	Definition
High-intensity interval training (HIIT)	Repetitive relatively short-interval exercise, performed with “all-out effort” or intensity ≥90% peak oxygen uptake or heart rate ≥ 90% peak heart rate (46, 47)
Combined exercise (COM)	Integrated modality of exercise that combines aerobic exercise and resistance training (12)
Resistance training (RT)	Exercise aimed at improving muscle strength, endurance, and size (48)
Aerobic exercise (AE)	Continuous exercise aimed at improving the efficiency and capacity of the cardiorespiratory system, such as walking, cycling, and jogging (48)
Mind–body exercises (MBE)	Exercise involves a sequence of movements and postures with musculoskeletal stretching and relaxation, breath control, and mental focus, such as yoga, tai-chi, and pilates (49)

Studies were excluded if they fell into any of the following categories: (a) Cross-over design studies; (b) Studies incorporating mechanical or external aids in the intervention; (c) Conference abstracts, experimental protocols, and systematic reviews; and (d) Studies where appropriate data could not be obtained.

### 2.4 Data extraction

Two separate reviewers (Xn-Z and Zd-L), were responsible for extracting data from studies that fulfilled the specified inclusion criteria. In instances where there were differences in their data extraction, a third reviewer, Md-L, was consulted to reach a consensus. The scope of the extracted data encompassed several categories, such as details regarding the first author, year and demographic details of the participants (including age, gender, and the total number of subjects involved). Moreover, information was also collected on the characteristics of the interventions studied—such as their type, duration, frequency, and intensity. Outcomes pertaining specifically to fatigue and muscular fitness in patients with MS were also documented, and the measurement method and unit of reported outcomes. If data were unavailable, the corresponding author was contacted up to three times within a three-week period.

### 2.5 Data coding and management

Based on the descriptions of the intervention details reported in the included studies, specific exercise intensities were coded according to the standards outlined in the *2024 Compendium of Physical Activities and its expansion* (16). The total intensity of each exercise unit was defined by multiplying the intensity of the specific activity [measured in Metabolic Equivalent of Task (MET)] by the duration of a single session and the weekly frequency, with the final result expressed in MET-minutes per week. The exercise frequency was represented by the total number of sessions per week, including multiple sessions per day, while the exercise duration was defined based on the descriptions provided in the articles. Additionally, if the duration of an exercise intervention gradually increased over several weeks, the average of the total duration was taken. Finally, to facilitate network connectivity and dose–response analysis, the estimated weekly MET-minutes were clustered into seven predefined categories: 0 (control), 200, 400, 600, 800, 1,000, and 2,000 MET-minutes/week (17).

### 2.6 Risk of bias and certainty of evidence

Two independent reviewers (Xn-Z and Zd-L) assessed the risk of bias (RoB) in the included studies according to the Cochrane Risk of Bias Tool (18). Any discrepancies were resolved through consultation with a third experienced reviewer (Md-L). Given the difficulty of blinding participants to exercise interventions, this aspect was not included in the overall RoB scoring. Instead, we considered the blinding of outcome assessors as a quality criterion. Subsequently, the certainty of evidence for the primary and secondary NMAs outcomes was graded using the Grading of Recommendations Assessment, Development, and Evaluation (GRADE) framework (19). The GRADE framework systematically evaluates the quality of evidence based on factors such as study limitations, consistency of results, directness of evidence, precision, and potential publication bias, providing a clear rating of confidence in the effect estimates.

### 2.7 Measures of treatment effect

This meta-analysis assessed the impact of exercise interventions by calculating the standardized mean difference (SMD) and standard deviations (SD) for changes observed between pre- and post-intervention periods. In cases where the SD was not explicitly reported in the primary studies, it was inferred using a range of statistical parameters, such as standard error, 95% confidence intervals, *p*-values, range values, or t-statistics (20). To facilitate comparison across studies, data were then converted to Hedges’ g SMD. For estimating the SD of the differences between pre- and post-intervention values, a correlation coefficient of 0.5 was assumed. This assumption is supported by a moderately accepted standard of measurement reliability found in the existing literature. The rationale behind selecting this coefficient was to mitigate potential variations in measurements taken before and after the intervention, thereby ensuring that the results remain both conservative and reliable (20).

### 2.8 Statistical analysis

#### 2.8.1 Primary analyses

To enable a simultaneous comparison of the relative effects of different exercise interventions, we began by assessing the transitivity assumption of the network meta-analysis (NMA). This was done through an examination of the key characteristics of each intervention and baseline participant data to ensure that the comparison across studies was valid (20). Network diagrams were then generated to visualize the direct treatment comparisons. Following this, we conducted the Bayesian NMA using the “Metainsight” tool (version 6.1.0),^1^ which implements the Bayesian framework through the R packages “Gemtc” and “BUGSnet” (21). This method utilizes a Markov chain Monte Carlo (MCMC) simulation to estimate the intervention effects along with their associated uncertainties (22, 23). Model convergence was confirmed through the application of Brooks-Gelman Rubin diagnostic statistics (24). To evaluate the heterogeneity of the results, we calculated the standard deviation (*τ*) along with the 95% credible intervals (CrI). Global inconsistency was checked by comparing model fit and variance using the deviance information criterion (DIC) and by contrasting the results of the consistency model against the uncorrelated mean effects model (25). When testing for inconsistency between direct and indirect effect estimates within the network, node-splitting analysis was employed, and results were considered significant if the *p*-value was below 0.05. Finally, we ranked the interventions based on their efficacy by calculating surface under the cumulative ranking curve (SUCRA) values. Forest plots were also generated to provide a visual summary of the results. The SUCRA values ranged from 0 to 100%, where higher values correspond to greater efficacy (26).

We further extended the analysis using a random-effects Bayesian model-based NMA (MBNMA) to investigate the dose–response relationship between exercise and outcomes such as fatigue and muscular fitness in patients with MS (27). The transitivity of the network, data consistency, and network connectivity were evaluated prior to this analysis, ensuring that no key assumptions of MBNMA were violated (ESM) (17, 28, 29). The effect sizes for exercise interventions on fatigue and muscular fitness were assessed using standardized mean differences (SMD) with 95% CrIs providing confidence intervals. To model the dose–response relationship, we applied several widely recommended functions, including the Emax model, restricted cubic splines, quadratic functions, and non-parametric approaches (30). These functions were compared by examining key fit indices such as the DIC, the between-study SD, model parameters, and residual deviance (31). Across all scenarios, the restricted cubic splines model showed superior fit, suggesting that it best captured the underlying dose–response relationship (ESM). The *β* coefficients generated from the model allowed us to estimate the minimum dose of exercise required to yield significant improvements in fatigue and muscular fitness. Furthermore, this model enabled the ranking of different exercise modalities based on their likelihood of triggering meaningful improvements (27). When interpreting the dose–response results, we considered findings statistically significant if the 95% CrI for the effect size excluded zero. Lastly, 95% prediction intervals were used to describe the potential variability in outcomes for future studies, providing insights into the potential impact of these exercise interventions when implemented in practice (32). The entire MBNMA and dose–response analysis was performed using the “MBNMAdose” package in R (version 4.3.1), and the graphical representation of the dose–response curves was accomplished with the “ggplot2” package. At last, to evaluate the effects of the findings, we performed a sensitivity analysis, specifically examining how studies with large residual deviance (≥2) might influence the overall model fit and the efficacy of the interventions (33).

#### 2.8.2 Additional analyses

To examine the potential moderating effects of these continuous variables on model fit—and to avoid possible biases introduced by subjective grouping criteria in subgroup analyses—network meta-regressions were applied. A common treatment effect across all interventions was assumed. The analysis focused on continuous variables, including patient age, disease duration, EDSS (Expanded Disability Status Scale), and the duration of the exercise program. In cases of missing data, mean imputation was employed to ensure comprehensive analysis (34). All statistical procedures were executed in the R environment, using the “Gemtc” package for conducting the network meta-regressions.

## 3 Results

### 3.1 Study selection and characteristics

A total of 4,420 studies were retrieved from the database mentioned earlier. After excluding duplicates and studies that did not match the topic based on titles and abstracts, 917 articles remained for full-text evaluation. Of these, 821 articles were excluded, leaving 84 randomized controlled trials (RCTs) for inclusion in the network meta-analysis (NMA). Among these, 55 studies reported outcomes related to fatigue, while 50 studies provided data on muscular fitness. In total, 3,786 participants were included, with a mean age of 42.46 years, an average disease duration of 8.36 years, and a mean Expanded Disability Status Scale (EDSS) of 3.33.

In studies that primarily targeted fatigue as the main outcome, aerobic exercise (AE; *n* = 25) emerged as the most frequently used intervention, while resistance training (RT; *n* = 23) was the second most implemented method. Conversely, in studies focusing on muscular fitness, resistance training (RT; *n* = 25) took the lead as the preferred intervention, with aerobic exercise (AE; *n* = 11) being the next most commonly applied approach. The exercise protocols varied significantly in frequency, ranging from 1 to 14 sessions per week, and the typical duration for each session was 51 min on average. Full details of the studies included in this analysis can be found in the ESM.

### 3.2 Risk of bias

**Figure 1:**
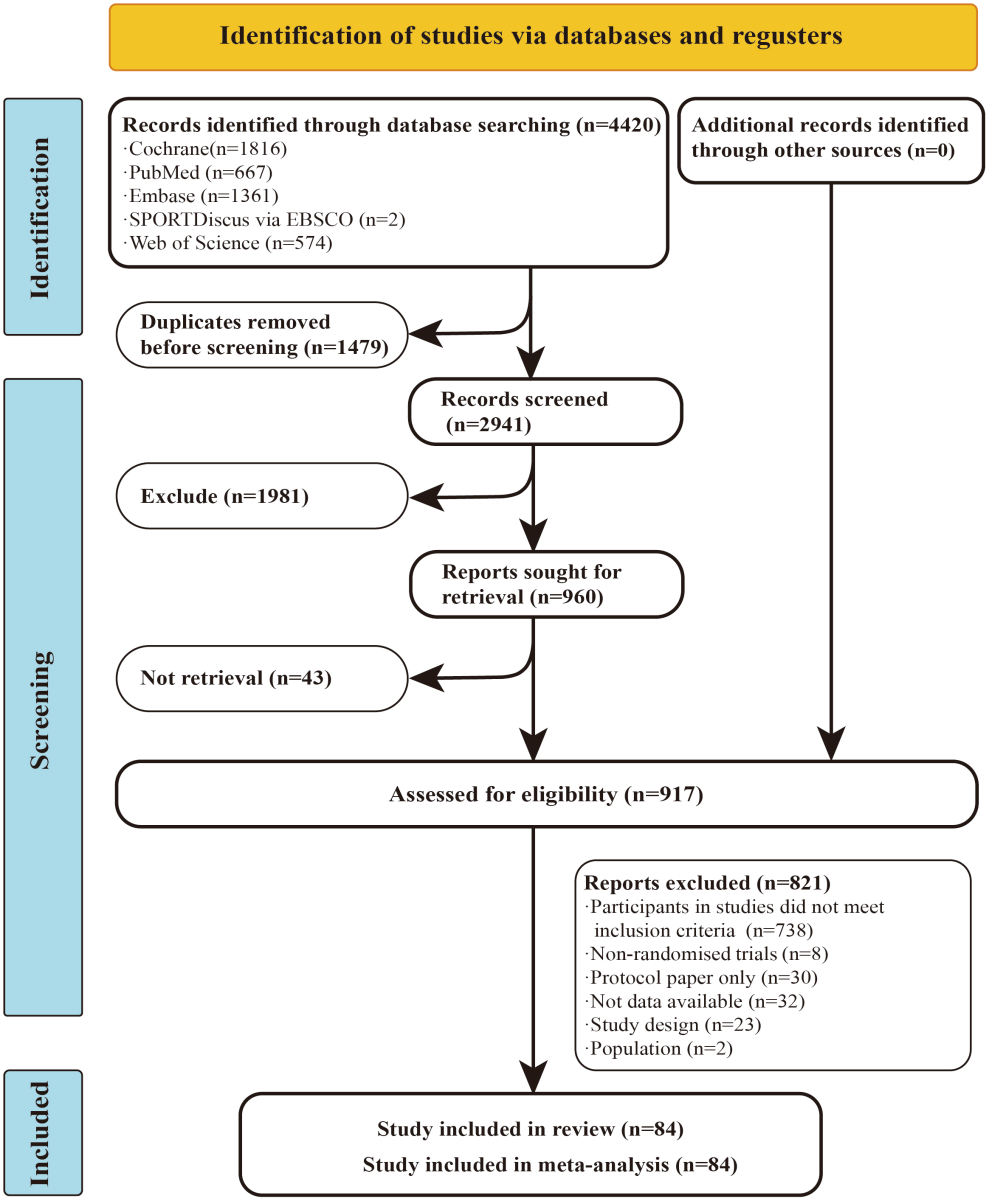
Literature review flowchart. RCT, randomized controlled trial.

Among the 84 studies included in this meta-analysis, 32 were identified as having a high risk of bias, 45 exhibited some concerns regarding bias, and 7 were categorized as having a low risk of bias. Detailed information on the risk of bias assessment can be found in the ESM (Figure 1).

### 3.3 Certainty of evidence

The quality of evidence concerning fatigue and muscular fitness in this study was evaluated as ranging from very low to moderate. Due to issues related to imprecision, risk of bias, and inconsistency, the five exercise interventions were determined to have a low certainty of evidence. A detailed summary of the study results is provided in the ESM.

### 3.4 NMAs

**Figure 2:**
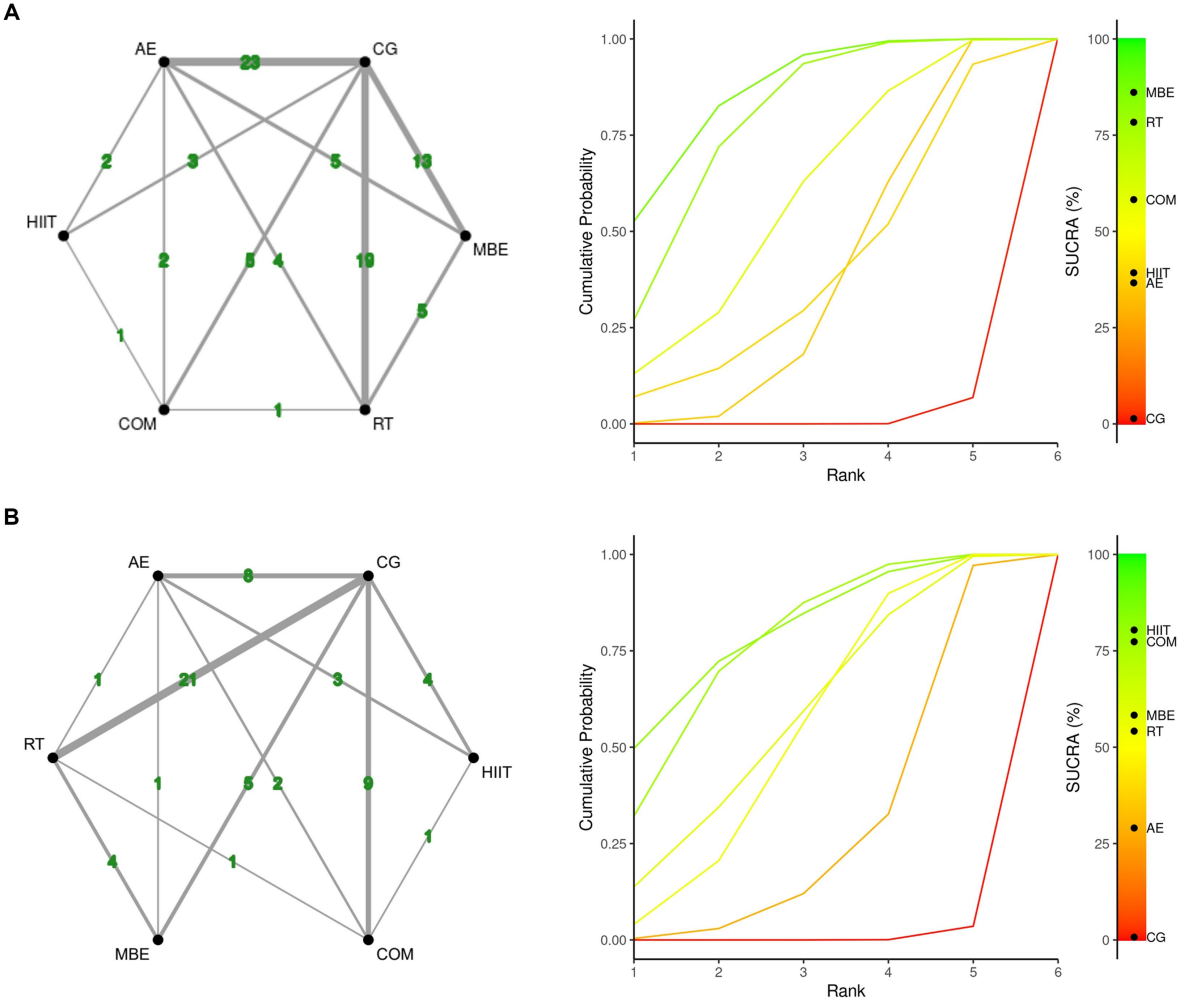
Network plot and the Bayesian ranking panel plot. **(A)** Fatigue. **(B)** Muscular fitness. The network plot (on the left) illustrates the direct and indirect comparisons in the network meta-analysis. The size of each node represents the number of participants in each intervention. The connections between the nodes indicate direct comparisons between different exercise interventions, with the thickness of the lines reflecting the amount of direct evidence. The Bayesian ranking panel plot (on the right), which uses the Surface Under the Cumulative Ranking Curve (SUCRA) values to assess the relative effectiveness of different exercise interventions. Higher SUCRA values and cumulative ranking curves closer to the upper left corner indicate better performance. HIIT, High-intensity interval training; COM, Combined exercise; RT, Resistance training; AE, Aerobic exercise; MBE, Mind–body exercises.

Figure 2 presents the core findings for the five distinct exercise interventions assessed in this research. When examining interventions aimed at reducing fatigue, the highest SUCRA value was observed for mind–body exercise (MBE) at 86.13%. This was followed in rank by resistance training (RT) at 78.52%, the combination of aerobic and resistance exercises (COM) at 58.27%, high-intensity interval training (HIIT) at 39.04%, and lastly, aerobic exercise (AE) at 36.65% (Figure 2A, Table 2; ESM Table S4a). For outcomes related to muscular fitness, HIIT exhibited the greatest improvement, with a SUCRA of 80.52%. This was closely followed by COM at 76.87%, MBE at 58.2%, RT at 54.36%, and AE at 29.28% (Figure 2B, Table 2; ESM Table S4b).

**Table 2:** League table of network comparisons of the effects of different exercise interventions on fatigue and muscular fitness.

(A) Fatigue					
MBE					
−0.07 (−0.47, 0.32)	RT				
−0.24 (−0.84, 0.33)	−0.17 (−0.71, 0.37)	COM			
−0.45 (−1.19, 0.27)	−0.38 (−1.09, 0.32)	−0.2 (−0.97, 0.55)	HIIT		
−0.42 (−0.83, −0.01)	−0.34 (−0.72, 0.02)	−0.17 (−0.7, 0.36)	0.03 (−0.64, 0.72)	AE	
−0.94 (−1.29, −0.6)	−0.87 (−1.17, −0.58)	−0.7 (−1.18, −0.21)	−0.49 (−1.14, 0.16)	−0.52 (−0.8, −0.25)	CG

(B) Muscular fitness					
HIIT					
−0.06 (−0.78, 0.63)	COM				
−0.23 (−1.03, 0.54)	−0.17 (−0.81, 0.45)	MBE			
−0.25 (−0.95, 0.42)	−0.19 (−0.67, 0.29)	−0.02 (−0.52, 0.49)	RT		
−0.48 (−1.17, 0.15)	−0.42 (−0.96, 0.09)	−0.26 (−0.87, 0.35)	−0.23 (−0.72, 0.24)	AE	
−0.87 (−1.53, −0.27)	−0.81 (−1.24, −0.41)	−0.64 (−1.14, −0.15)	−0.62 (−0.92, −0.35)	−0.39 (−0.8, 0.02)	CG

The data shown in the table are mean differences and 95% credible intervals. Exercises are reported in order of surface under the curve cumulative ranking.

HIIT, High-intensity interval training; COM, Combined exercise; RT, Resistance training; AE, Aerobic exercise; MBE, Mind–body exercises.

In addition, compared to the control group, most exercise interventions were effective in significantly reducing fatigue. MBE (SMD = −0.94; 95% CrI: −1.3 to −0.6), RT (SMD = −0.86; 95% CrI: −1.2 to −0.58), COM (SMD = −0.70; 95% CrI: −1.2 to −0.22), and AE (SMD = −0.52; 95% CrI: −0.8 to −0.25) all yielded notable reductions. The only exception was HIIT (SMD = −0.50; 95% CrI: −1.1 to 0.14), which did not show a statistically significant reduction in fatigue levels. Similarly, for muscular fitness improvements, HIIT (SMD = −0.86; 95% CrI: −1.5 to −0.27), COM (SMD = −0.81; 95% CrI: −1.2 to −0.41), MBE (SMD = −0.64; 95% CrI: −1.1 to −0.16), and RT (SMD = −0.62; 95% CrI: −0.89 to −0.16) were all found to significantly enhance muscular fitness, with AE (SMD = −0.39; 95% CrI: −0.79 to 0.0076) as the only intervention failing to reach statistical significance when compared to controls.

The ESM contains detailed outcomes from both the model fit assessment and the node-split method. No significant differences in fatigue outcomes were observed between the random-effects NMAs model (DIC = 250.3) and the uncorrelated mean effects model (DIC = 251.51). Similarly, for muscular fitness, the results remained consistent between the random-effects NMAs model (DIC = 204.1) and the uncorrelated mean effects model (DIC = 206.8). Across all exercise interventions, the node-split analysis failed to identify any statistically significant distinctions in both fatigue and muscular fitness outcomes.

Supplementary Figure S4 outlines the residual deviance per arm across the included studies, indicating that seven studies exceeded a residual deviance threshold of 2 for fatigue-related outcomes. Additionally, seven studies also showed residual deviance values greater than 2 in the context of muscular fitness outcomes.

### 3.5 Dose–response NMAs

**Figure 3:**
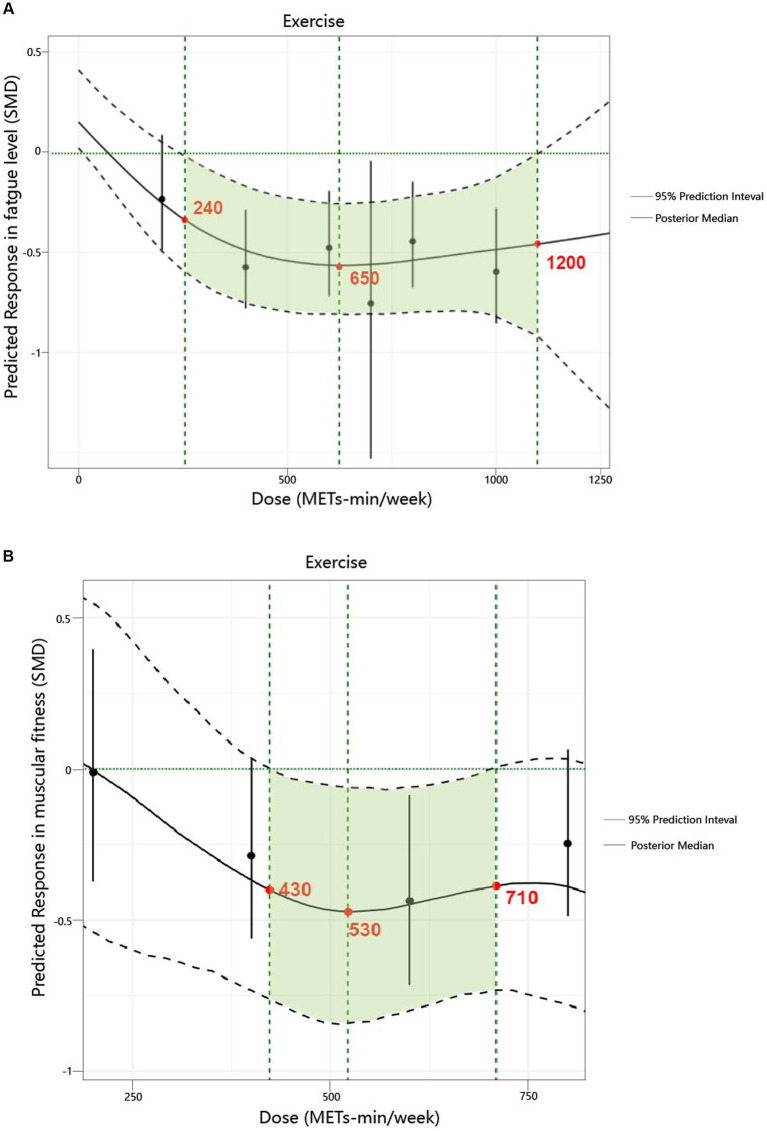
Dose–response relationship between total weekly total exercise volume and both fatigue and muscular fitness in patients with multiple sclerosis. **(A)** Fatigue. **(B)** Muscular fitness.

We found a non-linear dose–response relationship between the total exercise dose and fatigue levels as well as muscular fitness (Figure 3). Specifically, for fatigue levels, a significant response was observed starting at 240 METs-min/week (with the upper limit of the 95% CrI less than 0). At 600 METs-min/week, [which is the lower limit of the World Health Organization (WHO) recommended energy expenditure for physical activity (7)], the predicted response was SMD = −0.55; 95% CrI: −0.81 to −0.25; SD = 0.14. When the exercise dose reached 650 METs-min/week, the increase in the effect of its influence became very slow, with a predicted response of SMD = −0.56; 95% CrI: −0.81 to −0.26; SD = 0.14. At 1200 METs-min/week [which also represents the upper limit of the WHO recommended energy expenditure for physical activity (7)], the predicted response was SMD = −0.44; 95% CrI: −1.13 to −0.14; SD = 0.1.

Regarding muscular fitness levels, a significant response was observed starting at 430 METs-min/week (with the upper limit of the 95% CrI less than 0). When the exercise dose reached 530 METs-min/week, the increase in the effect of its influence became very slow, with a predicted response of SMD = −0.46; 95% CrI: −0.83 to −0.07; SD = 0.19. At 600 METs-min/week (7), the predicted response was SMD = −0.44; 95% CrI: −0.78 to −0.05; SD = 0.18. At 710 METs-min/week, the increase in the effect of its influence approached zero, with a predicted response of SMD = −0.38; 95% CrI: −0.73 to −0.01; SD = 0.19.

Supplementary Figure S16 provides a detailed illustration of the dose–response relationship for the five exercise modalities. The ranking analysis shows that RT (670 METs-min/week) has the highest probability of yielding the greatest outcome for fatigue level (ESM Table S8a), while HIIT (1,000 METs-min/week) has the highest probability of yielding the greatest outcome for muscular fitness (ESM Table S8b). Additionally, Table 3 provides practical recommendations based on the best predicted efficacy for different exercises and objectives.

**Table 3:** Exercise recommendations in patients with multiple sclerosis based on optimal dosage.

A. Exercise recommendations for reducing fatigue in patients with multiple sclerosis based on optimal dosage (650 METs-min/week).				
Type of exercise	Intensity	Energy expenditure^a^ (METs-min)	Optimal recommended accumulation^b^ (min/week)	Minimum recommendations for exercise prescription^c^ (sessions × min/per week)
MBE	Moderate	4 (code 02160)	~165	4 × ~40
				3 × ~55
RT	Moderate	3.5 (code 02054)	~190	4 × ~50
				3 × ~65
	Vigorous	6.5 (code 02057)	~100	4 × ~30
				3 × ~40
COM	Moderate	4.5 (mean of codes 01214, 02052)	~145	3 × ~50
	Vigorous	8 (mean of codes 01236, 02055)	~90	3 × ~30

B. Exercise recommendations for patients with multiple sclerosis to improve muscular fitness based on optimal dosage (530 METs-min/week).				
Type of exercise	Intensity	Energy expenditure^a^ (METs-min)	Optimal recommended accumulation (min/week)	Minimum recommendations for exercise prescription^b^ (sessions × min/per week)
HIIT	Moderate	7 (code 02210)	~75	4 × ~20
				3 × ~25
	Vigorous	11 (code 02214)	~50	3 × ~20
				2 × ~25
COM	Moderate	4.5 (mean of codes 01214, 02052)	~120	3 × ~40
				2 × ~60
	Vigorous	8 (mean of codes 01236, 02055)	~70	3 × ~25
				2 × ~35
MBE	Moderate	4 (code 02160)	~135	3 × ~50
				2 × ~70

HIIT, High-intensity interval training; COM, Combined exercise; RT, Resistance training; AE, Aerobic exercise; MBE, Mind–body exercises.

a Intensity coding was extracted from the *Compendium of Physical Activity* (35): Code 02160: Yoga, Power; Code 02054: Resistance (weight) training, multiple exercises, 8–15 reps at varied resistance; Code 02057: Body weight resistance exercises (e.g., squat, lunge, push-up, crunch), high intensity; Code 01214: Bicycling, stationary, 50 watts, light effort; Code 02052: Resistance (weight) training, squats, deadlift, slow or explosive effort; Code 01236: Bicycling, stationary, 200–229 watts, vigorous; Code 02055: Resistance Training, circuit, reciprocol supersets, peripheral hear action training; Code 02210: High intensity interval exercise, moderate effort; Code 02214: High intensity interval exercise, burpees, mountain climbers, squat jumps, Tabata, vigorous effort.

b Frequency and duration of each exercise, not counting warm-up and cool-down.

### 3.6 Network meta-regression

The results of the network meta-regression are presented in the Table 4. For fatigue outcomes, compared to the control group, EDSS and intervention duration did not significantly modulate the relative effect of exercise level. However, there was a significant correlation with the patient’s age (*β* = 0.70; 95% CrI: 0.32 to 1.08, DIC = 237.22) and disease duration (*β* = 0.64; 95% CrI: 0.21 to 1.07, DIC = 179.67). For the muscular fitness outcomes, compared to the control group, disease duration, EDSS, and intervention duration did not significantly modulate the relative effect of exercise level. However, there was a significant correlation with the patient’s age (*β* = 0.57; 95% CrI: 0.16 to 0.96, DIC = 204.19).

**Table 4:** Model fit summaries for univariate network meta-regression.

A				
Covariate	Fatigue			
	DIC	pD	Shared beta (Median and 95% CrI)	SD
Unadjusted	250.10	105.61	–	0.58 (0.41, 0.77)
Age	237.22	96.36	0.69 (0.32, 1.08)	0.49 (0.33, 0.69)
DESS	170.27	72.15	0.35 (−0.13, 0.87)	0.53 (0.34, 0.77)
Intervention duration (weeks)	249.96	105.47	0.13 (−0.22, 0.49)	0.58 (0.42, 0.78)
Disease duration	179.67	77.85	0.64 (0.21, 1.07)	0.53 (0.36, 0.75)

B				
Covariate	Muscular fitness			
	DIC	pD	Shared beta (Median and 95% CrI)	SD
Unadjusted	204.16	85.06	–	0.54 (0.35, 0.78)
Age	204.1	81.74	0.56 (0.16, 0.96)	0.45 (0.24, 0.69)
DESS	172.92	75.18	0.44 (−0.09, 0.97)	0.62 (0.39, 0.89)
Intervention duration (weeks)	205.54	86.04	0.05 (−0.43, 0.51)	0.55 (0.35, 0.79)
disease duration	140.11	61.55	0.46 (−0.09, 1.02)	0.55 (0.31, 0.83)

CrI, credible interval; DIC, deviance information criterion; SD, standard deviation; EDSS, Expanded Disability Status Scale.

## 4 Discussion

### 4.1 Principal findings

In this meta-analysis of randomized controlled trials, we investigated the effects of various exercise interventions on fatigue and muscular fitness in patients with MS, and we ranked their efficacy. Our findings indicate that MBE, COM, RT, and AE significantly reduce fatigue levels, while HIIT, RT, MBE, and COM can significantly improve muscular fitness. There were no significant differences in efficacy among the different exercise mobilities for either fatigue or muscular fitness. We also identified a non-linear dose–response relationship between exercise and levels of fatigue as well as muscular fitness. The optimal effective dose for reducing fatigue was estimated at 650 METs-min/week, which corresponds to 165 min of yoga (4 METs-min), 100 min of body weight resistance exercises (6.5 METs-min) per week (16). When the dose reached the range of 1,200 MET-min/week, the effects of exercise on fatigue approached zero (7). The optimal effective dose for improving muscular fitness was estimated at 530 METs-min/week, which corresponds to 75 min of HIIT (7 METs-min), 135 min of yoga (4 METs-min) per week (35). When the dose reached the range of 710 MET-min/week, the effects of exercise on muscular fitness approached zero. Additionally, we observed different dose–response patterns different exercise modalities. For fatigue, AE, COM, and MBE exhibited a U-shaped relationship, while other exercise modalities showed a nonlinear negative correlation. For muscular fitness, all exercise types demonstrated a nonlinear negative correlation. Finally, through meta-regression analysis, we found that patient’s age influences the effects of exercise interventions on both fatigue and muscular fitness, while disease duration also has an impact on the effects of exercise on fatigue.

### 4.2 Comparison with other studies

Previous reviews have primarily focused on whether exercise impacts patients with MS (1, 36). However, our study focused more on the effects of different exercise modalities on fatigue and muscular fitness. Fatigue has an incidence rate as high as 83% in MS, making it one of the most common symptoms and the one that most significantly affects patients’ quality of life (37). Several meta-analyses and a Cochrane review have investigated the overall impact of exercise on fatigue symptoms in patients with MS (13, 38, 39). These quantitative syntheses reported a moderate overall reduction in fatigue following exercise training (Cohen’s *d* = 0.45–0.57). Additionally, two reviews demonstrated that exercise can improve muscular fitness (Cohen’s *d* = 0.27) and that RT can enhance lower limb muscle strength. Based on the results of the network comparison, we conclude that mind–body exercise (MBE) and combined exercise (COM) rank relatively higher compared to other exercise modalities. A previous meta-analysis also highlighted that RT (SUCRA: 83.9%), COM (SUCRA: 77.9%), and MBE (SUCRA: 83.9%) are among the top-ranked exercise modalities affecting total fatigue in MS patients. Another study confirmed the impact of RT and COM on muscular fitness. This could be attributed to the fact that HIIT, RT, and COM may increase the available energy reserves (40) and induce neuroprotective mechanisms (41, 42), particularly as RT has been shown to improve efferent motor drive in this population (43). Moreover, MBE may normalize the deregulation of the hypothalamo-pituitary–adrenal (HPA) axis (44), as evidenced by a previous randomized controlled trial on yoga and pilates for MS patients, which demonstrated similar findings in Biochemical Parameters (45). Additionally, the differences in modality ranking observed in the aforementioned studies might be due to the classification of exercises like yoga into different categories in earlier research (13), and the inconsistent definitions of ‘combined exercise’ modalities.

We also explored, for the first time, the dose–response relationship in patients with MS and identified a non-linear dose–response relationship between exercise and both fatigue levels and muscular fitness. The results revealed that the minimum effective dose, optimal dose, and maximum safe dose for reducing fatigue were 240, 650, and 1,200 METs-min/week, respectively. For improving muscular fitness, the minimum effective dose, optimal dose, and maximum safe dose were 430, 530, and 710 METs-min/week, respectively. Unlike the WHO’s recommended 600 METs-min/week (7), our findings indicate that a dose as low as 240 METs-min/week can reduce fatigue, while 430 METs-min/week is sufficient to enhance muscular fitness. This reinforces the WHO’s “Some physical activity is better than none” initiative (7). Given that this population typically engages in low levels of health-promoting physical activity, these findings provide valuable evidence to support participation in exercise (1). Furthermore, the 650 METs-min/week (optimal dose for reducing fatigue) and 530 METs-min/week (optimal dose for improving muscular fitness) offer different options and references for patients with varying intervention goals.

### 4.3 Clinical implications

This meta-analysis has several important clinical implications. First, we found that MBE, COM, RT, and AE significantly reduce fatigue levels, while HIIT, RT, MBE, and COM can significantly improve muscular fitness. At the same time, there were no significant differences in efficacy among the various exercise modalities for either fatigue or muscular fitness. Second, in contrast to the WHO’s recommended 600 METs-min/week (7), our findings indicate that a dose as low as 240 METs-min/week is sufficient to improve fatigue in patients, while 430 METs-min/week can enhance muscular fitness. The optimal dose for reducing fatigue was found to be 650 METs-min/week, and 530 METs-min/week was identified as the optimal dose for improving muscular fitness. Third, we recommend incorporating a suitable weekly regimen of yoga for ≈165 min or bodyweight resistance exercises (moderate) for ≈190 min to optimally reduce fatigue. To achieve the best improvements in muscular fitness, a weekly regimen should include approximately 75 min of high-intensity interval training (HIIT) or 135 min of yoga. Moreover, although the regression analysis showed that EDSS did not significantly impact levels of fatigue or muscular fitness, more moderate exercise options should be considered for individuals who are obese, older, or have lower physical capabilities. Given the positive effects of various exercise modalities, healthcare providers should engage in discussions with patients to tailor exercise interventions according to their specific needs, physical abilities, and goals.

### 4.4 Strengths and limitations

This study has several key strengths. First, our research combines network analysis with a novel dose–response network analysis to investigate the effects of exercise on fatigue and muscular fitness in patients with multiple sclerosis. The integration of these methods allowed us to identify the optimal exercise modalities and doses. Second, our study included a large sample of MS patients and incorporated a wider range of exercise modalities, with some being reclassified, thereby providing sufficient statistical power and more refined comparative options. Finally, we conducted regression analyses on several key variables to examine the impact of potential moderating factors on the outcomes of exercise interventions, further enhancing the generalizability of our findings.

However, several limitations of our study must be acknowledged. First, the multidimensional nature of MS symptoms complicates its management, as MS is often associated with physical disability but also involves cognitive and psychosocial dimensions. These dimensions could act as confounding variables or mediators in the associations we studied. However, it is important to note that we could not account for these confounding variables in our analysis. Second, the dose–response analysis for some exercises did not show significant effects. This may be due to the limitations of the exercises themselves or an insufficient range of doses covered in the studies, which may not have been enough to detect relevant and significant dose effects. Therefore, we should approach the results of dose predictions with caution and emphasize the need for future research to focus more on the impact of different exercise doses on MS patients. Third, although we included 84 studies, not all studies provided comprehensive data, which impacted the sample size in the analysis. Furthermore, the network meta-analysis results were partially non-significant, all of which affected the power of the network meta-regression, which is an important limitation. Finally, the quality of evidence in this study was rated as very low to moderate, which may further affect the certainty of the evidence.

## 5 Conclusion

Exercise has been demonstrated to be a nonpharmacologic effective intervention for improving fatigue and muscular fitness, both of which are critical factors affecting the quality of life in patients with multiple sclerosis. This study is the first to explore the dose–response relationship between exercise and fatigue as well as muscular fitness in patients with multiple sclerosis. In this comprehensive meta-analysis, we confirmed the effectiveness of various exercise interventions in reducing fatigue levels and improving muscular fitness among patients with multiple sclerosis. Our findings revealed a nonlinear dose–response relationship between exercise and both outcomes. There is low-quality evidence suggesting that mind–body exercise (MBE), combined exercise (COM), resistance training (RT), and aerobic exercise (AE) significantly reduce fatigue levels, while high-intensity interval training (HIIT), RT, MBE, and COM can significantly enhance muscular fitness. However, there were no significant differences in efficacy among the different exercise modalities for either fatigue reduction or muscular fitness improvement. The optimal dose for reducing fatigue was identified as 650 METs-min/week, while 530 METs-min/week was found to be the optimal dose for improving muscular fitness. These studies provide valuable insights to help patients with multiple sclerosis make more informed and personalized decisions regarding exercise regimens tailored to their specific needs. Future large-scale randomized controlled trials are needed to investigate the effects of different exercise doses on patients with MS.

## Data Availability

The original contributions presented in the study are included in the article/Supplementary material, further inquiries can be directed to the corresponding author.

## Footnotes

^1^https://crsu.shinyapps.io/MetaInsight/
