# Supplementary material for: Comparison of different exercise modalities on fatigue and muscular fitness in patients with multiple sclerosis: a systematic review with network, and dose–response meta-analyses
Source: Front Neurol. 2024 Nov 26;15:1494368. doi: 10.3389/fneur.2024.1494368 (PMC11628374; doi:10.3389/fneur.2024.1494368)
Supplement: Supplementary file 1 [file Supplementary_file_1.docx]

**Comparison of Different Exercise Modalities on Fatigue and Muscular Fitness in Patients with Multiple Sclerosis: A Systematic Review with Network, and Dose–Response Meta‑Analyses**

Supplementary Files

# Search strategy

Cochrane Central Register of Controlled Trials (CENTRAL)

CENTRAL was searched using the following terms:

| 1. MeSH descriptor: [Multiple Sclerosis] explode all trees |
| --- |
| 1. (Sclerosis, Multiple):ti,ab,kw or (Sclerosis, Disseminated):ti,ab,kw or (MS (Multiple Sclerosis)):ti,ab,kw or (Disseminated Sclerosis):ti,ab,kw or (Multiple Sclerosis, Acute Fulminating):ti,ab,kw |
| 1. #1 or #2 |
| 1. MeSH descriptor: [Exercise] explode all trees |
| 1. MeSH descriptor: [Exercise Therapy] explode all trees |
| 1. MeSH descriptor: [Physical Education and Training] explode all trees |
| 1. MeSH descriptor: [Physical Fitness] this term only |
| 1. MeSH descriptor: [Physical Exertion] this term only |
| 1. MeSH descriptor: [Walking] explode all trees |
| 1. MeSH descriptor: [Running] explode all trees |
| 1. MeSH descriptor: [Swimming] this term only |
| 1. (physical NEAR/5 (education or training)) |
| 1. (cycling or bicycling or yoga or “tai-chi” or “tai chi” or “tai ji” or qigong or “qi gong” or "yoga" or "pilates" or "treadmill") |
| 1. (exercise* or exercising) |
| 1. #4 or #5 or #6 or #7 or #8 or #9 or #10 or #11 or #12 or #13 or #14 |
| 1. #3 and #15 |

MEDLINE

PubMed was searched using the following terms:

1. Multiple Sclerosis"[Mesh]
2. ((((Sclerosis, Multiple) OR (MS (Multiple Sclerosis))) OR (Sclerosis, Disseminated)) OR (Disseminated Sclerosis)) OR (Multiple Sclerosis, Acute Fulminating)
3. #1 OR #2
4. "Exercise"[Mesh]
5. ((((((((((((((((((((((((((((((Exercises[Title/Abstract]) OR (Physical Activity[Title/Abstract])) OR (Activities, Physical[Title/Abstract])) OR (Activity, Physical[Title/Abstract])) OR (Physical Activities[Title/Abstract])) OR (Exercise*, Physical[Title/Abstract])) OR (Physical Exercise*[Title/Abstract])) OR (Exercise, Aerobic[Title/Abstract])) OR (Aerobic Exercise*[Title/Abstract])) OR (Exercises, Aerobic[Title/Abstract])) OR (Exercise Training*[Title/Abstract])) OR (Training*, Exercise[Title/Abstract])) OR (moderate intensity continuous training[Title/Abstract])) OR (resistance training[Title/Abstract])) OR (strength training[Title/Abstract])) OR (combined training[Title/Abstract])) OR (sprint interval training[Title/Abstract])) OR (high intensity interval training[Title/Abstract])) OR (bicycling[Title/Abstract])) OR (cycling[Title/Abstract])) OR (Swimming[Title/Abstract])) OR (yoga[Title/Abstract])) OR (Pilates[Title/Abstract])) OR (tai-chi[Title/Abstract])) OR (tai chi[Title/Abstract])) OR (tai ji[Title/Abstract])) OR (qigong[Title/Abstract])) OR (qi gong[Title/Abstract])) OR (walking[Title/Abstract])) OR (treadmill training[Title/Abstract]))))
6. #4 OR #5
7. ((randomized controlled trial) OR (randomized)) OR (placebo)
8. #3 AND #6 AND #7

EMBASE

| 1. multiple sclerosis'/exp |
| --- |
| 1. sclerosis, multiple':ab,ti OR 'sclerosis, disseminated':ab,ti OR 'ms (multiple sclerosis)':ab,ti OR 'disseminated sclerosis':ab,ti OR 'multiple sclerosis, acute fulminating':ab,ti |
| 1. #1 OR #2 |
| 1. 'exercise'/exp |
| 1. 'physical activity'/exp |
| 1. 'sport'/exp |
| 1. 'exercise$':ab,ti OR 'exercising':ab,ti OR 'yoga':ab,ti OR 'tai-chi':ab,ti OR 'tai chi':ab,ti OR 'tai ji':ab,ti OR 'qigong':ab,ti OR 'qi gong':ab,ti OR 'walking':ab,ti OR 'treadmill':ab,ti OR 'swimming':ab,ti OR 'pilates':ab,ti |
| 1. #4 OR #5 OR #6 OR #7 |
| 1. 'randomized controlled trial':ab,ti OR 'randomized':ab,ti OR 'placebo':ab,ti |
| 1. #3 AND #8 AND #9 |

SPORTdiscus via EBSCO

| 1. AB Sclerosis, Multiple OR AB Multiple Sclerosis OR AB MS (Multiple Sclerosis) OR AB Sclerosis, Disseminated OR AB Disseminated Sclerosis OR AB Multiple Sclerosis, Acute Fulminating |
| --- |
| 1. AB Exercise OR AB Exercises OR AB Exercise Training OR AB Exercise Trainings OR AB Training, Exercise OR AB Trainings, Exercise OR AB moderate intensity continuous training OR AB strength training OR AB resistance training OR AB combined training OR AB sprint interval training OR AB yoga OR AB tai-chi OR AB tai chi OR AB tai ji OR AB qigong OR AB qi gong OR AB walking OR AB treadmill OR AB swimming OR AB pilates |
| 1. AB randomized controlled trial OR AB randomized OR AB placebo |
| 1. (S1 AND S2 AND S3)) AND (S1 AND S2 AND S3) |

Web of Science

1. (((((TS=(Multiple Sclerosis)) OR TS=(Sclerosis, Multiple)) OR TS=(Sclerosis, Disseminated)) OR TS=(MS (Multiple Sclerosis))) OR TS=(Disseminated Sclerosis)) OR TS=(Multiple Sclerosis, Acute Fulminating)
2. (((((((((((((((((((((((((((((TS=(Exercises)) OR TS=(Physical Activity)) OR TS=(Activities, Physical)) OR TS=(Activity, Physical)) OR TS=(Physical Activities)) OR TS=(Exercise*, Physical)) OR TS=(Physical Exercise*)) OR TS=(Exercise, Aerobic)) OR TS=(Aerobic Exercise*)) OR TS=(Exercises, Aerobic)) OR TS=(Exercise Training*)) OR TS=(Training*, Exercise)) OR TS=(moderate intensity continuous training )) OR TS=(resistance training )) OR TS=(strength training)) OR TS=(combined training)) OR TS=(sprint interval training)) OR TS=(high intensity interval training)) OR TS=(bicycling)) OR TS=(cycling)) OR TS=(Swimming)) OR TS=(yoga)) OR TS=(Pilates)) OR TS=(tai-chi)) OR TS=(tai chi)) OR TS=(tai ji)) OR TS=(qigong)) OR TS=(qi gong)) OR TS=(walking)) OR TS=(treadmill training)
3. ((TS=(randomized controlled trial)) OR TS=(randomized)) OR TS=(placebo)
4. #3 AND #2 AND #1

# Demographic characteristics of included studies

This supplementary file shows the Demographic characteristics of included studies. The study indicates the name of the author and the year of publication. The exact dose parameter indicates the exact estimated METs per week that participants accumulated in the study. The frequency is the number of days those participants were involved in physical activity. HIIT High-intensity interval training, COM Combined exercise, RT Resistance training, AE Aerobic exercise, MBE Mind–body exercises. UK, the United Kingdom. USA, the United States of America. NA, Not Available. EDSS Expanded Disability Status Scale.

| **Study** | **sample size (female)** | **Age**  **(mean±sd)** | **Intervention**  **duration**  **(weeks)** | **Frequency** | **Time**  **/ session** | **Exact dose** | **duration (years)** | **Type of MS**  **(RR/PP/SP/UN)** | **EDSS (mean±sd)** | **Region** |
| --- | --- | --- | --- | --- | --- | --- | --- | --- | --- | --- |
| Azra Ahmadi 2010 (1) | AE=10(10) | 36.8±9.17 | 8 | 3 | 30 | 432 | 5.6±3.3 | NA | 2.4±1.24 | Iran |
|  | CG=10(10) | 36.7±9.32 |  |  |  | 0 | 5±3.05 | NA | 2.25±1.25 |  |
| Azra Ahmadi 2010 (2) | MBE=11(11) | 32.27±8.68 | 8 | 3 | 65 | 487.5 | 4.72±5.62 | NA | 2±1.09 | Iran |
|  | CG=10(10) | 36.7±9.32 |  |  |  | 0 | 5±3.05 | NA | 2.25±1.25 |  |
| Azra Ahmadi 2013 | AE=10(10) | 36.8±9.17 | 8 | 3 | 30 | 432 | 5.6±3.3 | NA | 2.4±1.24 | Iran |
|  | MBE=11(11) | 32.27±8.68 | 8 | 3 | 65 | 487.5 | 4.72±5.62 | NA | 2±1.09 |  |
|  | CG=10(10) | 36.7±9.32 |  |  |  | 0 | 5±3.05 | NA | 2.25±1.25 |  |
| Zuhal Abasıyanık 2019 | RT=21（14） | 48.24±11.79 | 8 | 1 | 60 | 180 | 9.83±8.7 | 14/0/3 | 3.24±1.77 | Turkey |
|  | MBE=21（16） | 42.5±6.76 | 8 | 1 | 60 | 108 | 12.59±6.23 | 14/0/2 | 3.06±1.65 |  |
| Felipe Aidar 2018 (1) | COM=14（10） | 41.3±7.3 | 12 | 3 | 60 | 954 | NA | NA | 4.85±1.65 | Brazil |
|  | CG=14（9） | 43.6±7.6 |  |  |  | 0 | NA | NA | 4.15±2.17 |  |
| Felipe Aidar 2018 (2) | RT=13（8） | 42.8±8 | 12 | 3 | 50 | 525 | NA | NA | 4.27±1.29 | Brazil |
|  | CG=13（9） | 43.6±7.7 |  |  |  | 0 | NA | NA | 4.29±1.59 |  |
| Lisa Baquet 2018 | AE=34(21) | 38.2±9.6 | 12 | 3 | 60 | 774 | 6.8±5.5 | 34/0/0 | 1.7±0.9 | Germany |
|  | CG=34(25) | 39.6±9.7 |  |  |  | 0 | 5.7±6.3 | 34/0/0 | 1.8±1 |  |
| Laura Bonzano 2019 | RT=15（11） | 48.3±10 | 8 | 3 | 60 | 450 | 1.03±0.79 | 12/0/3 | 4.2±1.5 | Italy |
|  | CG=15（10） | 51.1±11 |  |  |  | 0 | 0.92±0.77 | 12/0/3 | 4.5±1.3 |  |
| I. Bulguroglu 2017 | MBE=13(0) | 37±2.63 | 8 | 2 | 75 | 420 | 5±2 | NA | 2±0.5 | Turkey |
|  | CG=13(0) | 40±4.25 |  |  |  | 0 | 3±1.88 | NA | 1±0.38 |  |
| Jacob Callesen 2019 | RT=23(22) | 52±6.67 | 10 | 2 | 60 | 420 | 15±6.67 | 12/1/4 | 4±1.11 | Denmark |
|  | CG=20(18) | 56±6.67 |  |  |  | 0 | 11±10.37 | 12/3/3 | 3.5±0.74 |  |
| Johnny Collett 2010 | AE=20(16) | 52±8 | 12 | 2 | 20 | 160 | 15±8 | 8/2/10/0 | NA | UK |
|  | COM=20(11) | 55±10 | 12 | 2 | 20 | 232 | 12±11 | 7/3/7/0 | NA |  |
|  | HIIT=21(16) | 50±10 | 12 | 2 | 20 | 272 | 11±7 | 7/2/8/1 | NA |  |
| U. Dalgas 2009 | RT=19（13） | 47.7±10.38 | 12 | 2 | 60 | 696 | 6.6±5.8 | 15/0/0 | 3.7±0.9 | Denmark |
|  | CG=19（12） | 49.1±8.44 |  |  |  | 0 | 8.1±6 | 16/0/0 | 3.9±0.9 |  |
| U. Dalgas 2010 (1) | RT=15（10） | 47.7±10.38 | 12 | 2 | 60 | 696 | 6.6±5.8 | 15/0/0 | 3.7±0.9 | Denmark |
|  | CG=16（10） | 49.1±8.44 |  |  |  | 0 | 8.1±6 | 16/0/0 | 3.9±0.9 |  |
| Louisa S. DeBolt 2004 | RT=19（15） | 45.8±7.9 | 8 | 3 | 60 | 540 | 8±5.9 | NA | 2.6±1.2 | USA |
|  | CG=18（13） | 39.7±9.1 |  |  |  | 0 | 6.1±4.2 | NA | 2.8±0.7 |  |
| C. Dettmers 2009 | COM=15（10） | 45.8±7.9 | 3 | 3 | 45 | 472.5 | 8±5.9 | 13/0/2 | 2.6±1.2 | Germany |
|  | RT=15（11） | 39.7±9.1 | 3 | 3 | 45 | 270 | 6.1±4.2 | 10/3/2 | 2.8±0.7 |  |
| Peter Feys 2015 | AE=10（3） | 61±8.8 | 8 | 3 | 30 | 271 | 21.1±9.9 | 1/1/7 | NA | Belgium |
|  | CG=10（5） | 53.1±6 |  |  |  | 0 | 13.2±6.8 | 0/1/7 | NA |  |
| Peter Feys 2019 | AE=21(20) | 36.6±8.5 | 12 | 3 | 40 | 420 | 8.1±6.1 | NA | NA | Belgium |
|  | CG=21(18) | 44.4±8.5 |  |  |  | 0 | 9.2±5.3 | NA | NA |  |
| Marius S. Fimland 2010 | RT=7（3） | 53±4 | 3 | 5 | 40 | 1200 | 8±1 | NA | 4.6±0.4 | Norway |
|  | CG=7（3） | 54±2 |  |  |  | 0 | 8±1 | NA | 3.5±0.5 |  |
| Zahra Golzari 2010 | COM=10（10） | 32.15±7.57 | 8 | 3 | 55 | 660 | NA | 10/0/0 | 2.14±1.06 | Iran |
|  | CG=10（10） | 33.75±8.18 |  |  |  | 0 | NA | 10/0/0 | 1.95±1.06 |  |
| Tanja Grubić Kezele 2019 (1) | RT=10（4） | 53.9±10.7 | 4 | 5 | 70 | 700 | NA | 6/0/3 | 6.5±1.75 | Croatia |
|  | CG=9（3） | 48.2±9.3 |  |  |  | 0 | NA | 4/2/4 | 7±1.63 |  |
| Arzu Guclu-Gunduza 2014 | MBE=18（0） | 36±8.15 | 8 | 2 | 60 | 336 | 2±4.64 | NA | 2±2.04 | Turkey |
|  | RT=8（0） | 36±12.96 | 8 | 2 | 60 | 320 | 1.75±2.27 | NA | 1.75±1.58 |  |
| Lisa Harvey 1999 | RT=6（5） | 38±2 | 8 | 14 | 20 | 980 | 5±1.63 | NA | NA | UK |
|  | AE=6（5） | 49±4.5 | 8 | 7 | 40 | 980 | 5±3.5 | NA | NA |  |
|  | CG=5（4） | 43±4.5 |  |  |  | 0 | 10±4.38 | NA | NA |  |
| Seyedeh Shelir Hosseini 2018 | RT=9（5） | 32.9±8.13 | 8 | 3 | 35 | 367.5 | NA | NA | NA | Iran |
|  | MBE=9（5） | 31.3±7.09 | 8 | 3 | 65 | 487.5 | NA | NA | NA |  |
|  | CG=8（4） | 33±9.74 |  |  |  | 0 | NA | NA | NA |  |
| Mehdi Kargarfard 2017 | RT=17(17) | 36.5±9 | 8 | 2.5 | 35 | 463.75 | 6.4±2.3 | 17/0/0 | 3.4±1.1 | Iran |
|  | CG=15(15) | 36.2±7.4 |  |  |  | 0 | 6.1±2 | 15/0/0 | 3.7±1 |  |
| Arno Kerling 2015 | AE=30(24) | 42.3±9 | 12 | 2 | 40 | 400 | NA | NA | 2.6±1.1 | Germany |
|  | COM=30(20) | 45.6±11.4 | 12 | 2 | 40 | 347.2 | NA | NA | 3.1±1.3 |  |
| Tue Kjølhede 2015 | RT=18（0） | 43.2±8.1 | 24 | 2 | 60 | 600 | 5±6.88 | 18/0/0 | 3±0.5 | Denmark |
|  | CG=17（0） | 43.2±8.1 |  |  |  | 0 | 5±6.88 | 17/0/0 | 3±0.5 |  |
| YC Learmonth 2011 | COM=17(13) | 51.4±8.06 | 12 | 2 | 60 | 738 | 13.4±6.4 | NA | 6.14±0.36 | UK |
|  | CG=11(7) | 51.8±8 |  |  |  | 0 | 12.6±8.1 | NA | 5.82±0.51 |  |
| Carlos Medina-Perez 2014 | RT=20（0） | 49.6±11 | 12 | 2 | 30 | 300 | 11.3±6.1 | 30/0/0 | 4.5±2.1 | Spain |
|  | CG=20（0） | 46.2±7.5 |  |  |  | 0 | 12.2±4.5 | 12/0/0 | 46.2±0.5 |  |
| Carlos Medina-Perez 2016 | RT=39（20） | 45.6±8.65 | 12 | 2 | 30 | 300 | 10.9±5.51 | 20/0/0 | 3.9±0.85 | Spain |
|  | CG=39（20） | 41.3±10.04 |  |  |  | 0 | 4.2±1.18 | 20/0/0 | 4.2±1.18 |  |
| Mahbubeh Moradi 2015 | RT=10（0） | 34.38±11.07 | 8 | 3 | 40 | 600 | 8.12±4.79 | 5/0/5 | 3±1.25 | Iran |
|  | CG=10（0） | 33.13±7.08 |  |  |  | 0 | 6.5±5.78 | 6/0/4 | 3±1 |  |
| Asiye Tuba Ozdogar 2020 | RT=19(12) | 43.6±10.5 | 8 | 1 | 45 | 135 | 6.43±5.9 | 18/0/0 | 2.11±0.9 | Turkey |
|  | CG=20(15) | 37.9±12.4 |  |  |  | 0 | 5.93±4.2 | 18/0/2 | 2.25±1.2 |  |
| Jack H. Petajan 1996 | AE=21（15） | 41.1±2 | 15 | 3 | 50 | 870 | 9.3±1.6 | NA | 3.8±0.3 | USA |
|  | CG=25（16） | 39±1.7 |  |  |  | 0 | 6.2±1.1 | NA | 2.9±0.3 |  |
| Bahram Sangelaji 2016 | COM=10（6） | 35.8±8.42 | 8 | 4 | 40 | 800 | NA | 10/0/0 | 1.33±0.66 | Iran |
|  | CG=10（6） | 33.63±6.92 |  |  |  | 0 | NA | 10/0/0 | 1.81±0.53 |  |
| Karl-Heinz Schulz 2004 | AE=15(11) | 39±9 | 8 | 2 | 30 | 360 | 11.4±1.6 | 19/2/5 | 2±1.4 | Germany |
|  | CG=13(8) | 40±11 |  |  |  | 0 | 11.4±1.6 | 19/2/5 | 2.5±0.8 |  |
| Jacob J Sosnoff 2014 | RT=14（11） | 60.1±6.3 | 12 | 3 | 60 | 666 | 13.9±6.7 | 10/2/1 | 5.5±5.19 | USA |
|  | CG=13（10） | 60.1±6 |  |  |  | 0 | 17.7±11.3 | 10/3/1 | 5.5±3.7 |  |
| Alexander Tallner 2016 | COM=59(44) | 40.9±10.4 | 12 | 3 | 60 | 720 | 9.8±9.2 | 52/0/7 | 2.8±0.8 | Germany |
|  | CG=67(50) | 40.7±9.5 |  |  |  | 0 | 9.2±7.2 | 57/0/10 | 2.7±0.8 |  |
| Viviane Regina Leite Moreno Ultramari 2020 | MBE=11（8） | 31.81±8.61 | 8 | 2 | 60 | 360 | 4.9±3.01 | 9/2/0 | 2.4±1.13 | Brazil |
|  | CG=10（7） | 36.36±12.13 |  |  |  | 0 | 8.72±6.21 | 9/0/2 | 2.36±1.12 |  |
| Inez Wens 2015 | COM=11（9） | 47±3 | 12 | 2.5 | 60 | 1200 | NA | NA | 2.5±0.3 | Belgium |
|  | CG=11（6） | 43±3 |  |  |  | 0 | NA | NA | 2.3±0.3 |  |
| Inez Wens 2016 | COM=15（9） | 42±3 | 24 | 2.5 | 30 | 337.5 | NA | NA | 2.7±0.3 | Belgium |
|  | CG=7（5） | 44±2 |  |  |  | 0 | NA | NA | 2.6±0.3 |  |
| Hui-Ju Young 2018 | AE=27(22) | 49.67±9.4 | 12 | 3 | 60 | 630 | 13.56±8.26 | NA | 2.37±2.13 | USA |
|  | MBE=26(20) | 48.35±9.95 | 12 | 3 | 60 | 720 | 10.98±5.57 | NA | 1.58±1.9 |  |
|  | CG=24(21) | 47.29±10.33 |  |  |  | 0 | 13.38±8.5 | NA | 2.57±2.01 |  |
| I.M. Alguacil Diego 2012 | RT=18(10) | 43±17 | 1 | 5 | 50 |  | NA | NA | 3.99±0.8 | Spain |
|  | CG=16(8) | 44±20 |  |  |  | 0 | NA | NA | 4.58±0.36 |  |
| Deborah Backus 2020 | RT=12(7) | 56.17±10.01 | 12 | 2.5 | 30 | 450 | NA | NA | 7.2±0.42 | USA |
|  | CG=9(6) | 54.67±11.55 |  |  |  | 0 | NA | NA | 7.58±0.9 |  |
| Dena Sadeghi Bahmani 2019 | AE=28(28) | 37.96±8.69 | 8 | 3 | 35 | 577.5 | 6.92±6.81 | NA | 2.46±1.5 | Switzerland |
|  | RT=27(27) | 39.17±8.66 | 8 | 3 | 45 | 499.5 | 8.13±6.37 | NA | 3.38±1.87 |  |
|  | CG=26(26) | 37.9±9.91 |  |  |  | 0 | 7.21±6.57 | NA | 7.21±6.57 |  |
| Burcu Duyur Çakıt 2010 | AE=15(10) | 36.4±10.5 | 8 | 2 | 45 | 450 | 9.2±5 | NA | NA | Turkey |
|  | RT=15(12) | 43±10.2 | 8 | 2 | 45 | 270 | 6.2±2.2 | NA | NA |  |
|  | CG=15(10) | 35.5±10.9 |  |  |  | 0 | 6.6±2.4 | NA | NA |  |
| Anna Carling 2017 | RT=25(19) | 61.64±11.25 | 7 | 2 | 60 | 360 | NA | 0/8/17 | 6.16±0.45 | Sweden |
|  | CG=26(16) | 54.73±8.16 |  |  |  | 0 | NA | 6/5/15 | 6.06±0.54 |  |
| A Carter 2014 | COM=60(43) | 45.7±9.1 | 12 | 3 | 60 | 900 | 9.2±7.9 | 51/2/7 | 3.8±1.5 | UK |
|  | CG=60(43) | 46±8.4 |  |  |  | 0 | 8.4±7.4 | 47/2/11 | 3.8±1.5 |  |
| U Dalgas 2010 (2) | RT=19(12) | 47.7±10.4 | 12 | 2 | 60 | 696 | 6.6±5.9 | NA | 3.7±0.9 | Denmark |
|  | CG=19(13) | 49.1±8.4 |  |  |  | 0 | 8.1±6 | NA | 3.9±0.9 |  |
| Elham Eftekhari 2018 | MBE=15(15) | 34.46±7.29 | 8 | 3 | 60 | 324 | NA | NA | NA | Iran |
|  | CG=15(15) | 31.41±8.89 |  |  |  | 0 | NA | NA | NA |  |
| Ramon Gomez-Illan 2020 | RT=13(9) | 45.31±11.06 | 8 | 3 | 60 | 1080 | NA | NA | 2.38±0.98 | Spain |
|  | CG=13(12) | 41.31±9.58 |  |  |  | 0 | NA | NA | 2.81±1.33 |  |
| Ali Hasanpour Dehkordi 2016 | MBE=30(30) | NA | 12 | 3 | 65 | 448.5 | NA | NA | NA | Iran |
|  | AE=30(30) | NA | 12 | 3 | 45 | 472.5 | NA | NA | NA |  |
|  | CG=30(1) | NA |  |  |  | 0 | NA | NA | NA |  |
| Jeffrey R. Hebert 2011 | RT=12(9) | 46.8±10.5 | 6 | 2 | 40 | 400 | 6.5±5.6 | NA | NA | USA |
|  | AE=13(11) | 42.6±10.4 | 6 | 2 | 40 | 400 | 5.1±3.2 | NA | NA |  |
|  | CG=13(11) | 50.2±9.2 |  |  |  | 0 | 9.1±7.3 | NA | NA |  |
| Martin Heine 2017 | HIIT=43(32) | 43.1±9.8 | 16 | 3 | 30 | 792 | 7±5.93 | 31/9/3 | 2.5±1.11 | Netherlands |
|  | CG=46(33) | 48.2±9.2 |  |  |  | 0 | 12±12.59 | 34/7/5 | 3±1.48 |  |
| M Garrett 2012 | RT=80(63) | 51.7±10 | 10 | 1 | 60 | 180 | 9.8±7 | 33/9/13/9 | NA | Ireland |
|  | MBE=77(54) | 49.6±10 | 10 | 1 | 60 | 138 | 11.6±8 | 38/8/7/9 | NA |  |
|  | CG=72(63) | 48.8±11 |  |  |  | 0 | 10.6±8.2 | 27/3/10/8 | NA |  |
| Neasa Hogan 2014 | RT=45(28) | 57±14.81 | 10 | 1 | 60 | 180 | 18±11.85 | 13/8/20/7 | NA | Ireland |
|  | MBE=16(10) | 58±11.85 | 10 | 1 | 60 | 138 | 13±11.85 | 4/2/5/2 | NA |  |
|  | CG=19(17) | 49±8.89 |  |  |  | 0 | 10±4.44 | 5/5/5/0 | NA |  |
| Alon Kalron 2016 | MBE=25(16) | 42.9±7.2 | 12 | 7 | 20 | 420 | 11.3±6.9 | NA | 4.1±1.1 | Israel |
|  | RT=25(16) | 44.3±6.6 | 12 | 7 | 20 | 392 | 12.4±5.7 | NA | 4.6±1.3 |  |
| Fatemeh Karami 2018 | RT=25(19) | 32.7±7.4 | 12 | 3 | 60 | 540 | 5.04±3.12 | NA | NA | Iran |
|  | CG=25(19) | 32.7±7.4 |  |  |  | 0 | 5.04±3.12 | NA | NA |  |
| Mehdi Kargarfard 2012 | AE=16(16) | 33.7±8.6 | 8 | 3 | 60 | 954 | 4.9±2.3 | NA | 2.9±0.9 | Iran |
|  | CG=16(16) | 31.6±7.7 |  |  |  | 0 | 4.6±1.9 | NA | 3±0.7 |  |
| Tanja Grubić Kezele 2019 (2) | RT=10(6) | 53.9±10.7 | 4 | 5 | 60 | 900 | NA | 4/2/4 | 6.5±1.75 | Croatia |
|  | CG=9(6) | 48.2±9.3 |  |  |  | 0 | NA | 6/0/3 | 7±1.63 |  |
| H. Kooshiar 2015 | AE=20(20) | 29.24±7.98 | 8 | 3 | 45 | 715.5 | 1.55±0.8 | NA | 2.5±1.1 | Iran |
|  | CG=20(20) | 29.24±7.98 |  |  |  | 0 | 1.55±0.8 | NA | 2.5±1.1 |  |
| Fadime Küçük 2016 | MBE=11(7) | 47.2±9.5 | 8 | 2 | 50 | 280 | 14.8±7.4 | NA | 3.2±2.2 | Turkey |
|  | RT=9(6) | 49.7±8.9 | 8 | 2 | 50 | 300 | 14.2±9.5 | NA | 2.8±1.4 |  |
| Ruth McCullagh 2008 | AE=17(14) | 33.58±6.1 | 12 | 3 | 60 | 900 | 5±3.52 | 8/0/4 | NA | Ireland |
|  | CG=13(10) | 40.5±12.68 |  |  |  | 0 | 5.4±4.35 | 9/0/3 | NA |  |
| Motahare Mokhtarzade 2017 | HIIT=25(25) | 32.04±2.81 | 8 | 3 | 50 | 1050 | 2.69±1.84 | NA | 1.84±0.35 | Iran |
|  | CG=20(20) | 31.27±3.28 |  |  |  | 0 | 3.47±1.26 | NA | 1.57±0.64 |  |
| S Mostert 2002 | AE=13(10) | 45.23±8.66 | 4 | 5 | 30 | 1050 | 11.2±8.5 | NA | 4.6±1.2 | Switzerland |
|  | CG=13(11) | 43.92±13.9 |  |  |  | 0 | 12.6±8.1 | NA | 4.5±1.9 |  |
| Hossein Negahban 2013 | AE=12(0) | 36.33±7.62 | 5 | 3 | 40 | 480 | 8.5±6.76 | NA | 3.5±1.13 | Iran |
|  | CG=12(0) | 36.83±8.74 |  |  |  | 0 | 7.22±2.86 | NA | 3.83±1.39 |  |
| B.S. Oken 2004 | AE=21(18) | 48.8±10.4 | 24 | 1 | 40 | 92 | NA | NA | 2.9±1.7 | USA |
|  | MBE=26(24) | 49.8±7.4 | 24 | 1 | 90 | 387 | NA | NA | 3.2±1.7 |  |
|  | CG=22(22) | 48.4±9.8 |  |  |  | 0 | NA | NA | 3.1±2.1 |  |
| Nazanin Razazian 2016 | MBE=18(18) | 33.11±6.6 | 8 | 3 | 60 | 414 | 6.78±0.65 | NA | 3.25±1.24 | Iran |
|  | AE=18(18) | 35.39±6.89 | 8 | 3 | 60 | 954 | 7.11±0.9 | NA | 3.44±0.95 |  |
|  | CG=18(18) | 33.33±7.4 |  |  |  | 0 | 6.9±0.9 | NA | 3.89±1.02 |  |
| Sofia Straudi 2014 | AE=12(7) | 49.922±7.51 | 2 | 5 | 120 | 2316 | 12.16±6.91 | 4/5/3 | 4.95±0.61 | Italy |
|  | CG=12(10) | 55.25±13.82 |  |  |  | 0 | 18.25±9.46 | 2/5/5 | 4.83±0.49 |  |
| Ela Tarakci 2013 | RT=55(37) | 41.49±9.37 | 12 | 3 | 60 | 720 | 9±4.71 | 32/10/9 | 4.38±1.37 | Turkey |
|  | CG=55(35) | 39.65±11.18 |  |  |  | 0 | 8.42±5.38 | 33/8/7 | 4.21±1.44 |  |
| M van den Berg 2006 | AE=10(0) | NA | 4 | 3 | 30 | 522 | NA | NA | NA | UK |
|  | CG=9(0) | NA |  |  |  | 0 | NA | NA | NA |  |
| Orjana Velikonja 2010 | RT=10(0) | NA | 10 | 1 | 60 | 348 | NA | NA | NA | Slovenia |
|  | MBE=10(0) | NA | 10 | 1 | 60 | 258 | NA | NA | NA |  |
| S Briken 2014 | RT=11(6) | 49.1±8.5 | 9 | 2.5 | 45 | 337.5 | 17.1±7.2 | NA | 5.2±0.9 | Germany |
|  | AE=12(7) | 48.8±6.8 | 9 | 2.5 | 45 | 562.5 | 14.1±5.4 | NA | 5±0.8 |  |
|  | CG=11(7) | 50.4±7.6 |  |  |  | 0 | 18.9±9.8 | NA | 4.9±0.9 |  |
| Janina M Burschka 2014 | MBE=15(10) | 42.6±9.4 | 24 | 2 | 90 | 1080 | 6±4.7 | NA | NA | Germany |
|  | CG=17(12) | 43.6±8 |  |  |  | 0 | 7.8±6.8 | NA | NA |  |
| KJ Dodd 2011 | RT=39(28) | 47.7±10.8 | 10 | 2 | 45 | 540 | NA | NA | NA | Australia |
|  | CG=37(27) | 50.4±9.6 |  |  |  | 0 | NA | NA | NA |  |
| Georgina Sutherland 2001 | AE=11(6) | 47.18±4.75 | 10 | 3 | 45 | 715.5 | 7±5.59 | NA | NA | Australia |
|  | CG=11(6) | 45.45±5.05 |  |  |  | 0 | 6.18±3.63 | NA | NA |  |
| Furkan Bilek 2022 | COM=16(15) | 28.31±5.89 | 6 | 3 | 60 | 630 | 5.44±3.92 | 16/0/0 | 1.69±0.85 | Turkey |
|  | RT=16(12) | 32.5±8.75 | 6 | 3 | 30 | 270 | 7.66±5.05 | 16/0/0 | 1.97±0.89 |  |
| Kader Eldemir 2023 | MBE=15(14) | 41±7.82 | 6 | 3 | 60 | 504 | 10±5.19 | NA | 1.5±1.11 | Turkey |
|  | CG=15(14) | 38.4±10.86 |  |  |  | 0 | 8±5.19 | NA | 1.5±1.48 |  |
| Karl M Fleming 2021 | MBE=39(36) | 46.7±10 | 8 | 2 | 40 | 224 | NA | NA | NA | Ireland |
|  | CG=41(33) | 47.4±10.2 |  |  |  | 0 | NA | NA | NA |  |
| Luca Correale 2021 | COM=14(14) | 45.4±7.2 | 12 | 2 | 60 | 600 | NA | NA | NA | Italy |
|  | CG=13(13) | 48.3±6.1 |  |  |  | 0 | NA | NA | NA |  |
| Luis Andreu-Caravaca 2022 | RT=18（9） | 44.89±10.62 | 10 | 3 | 50 | 750 | NA | NA | 3.17±1.65 | Spain |
|  | CG=12（5） | 48.36±10.23 |  |  |  | 0 | NA | NA | 3.27±1.3 |  |
| Martin Langeskov-Christensen 2022 | AE=43(26) | 44±9.5 | 3 | 2.5 | 45 | 675 | 10.9±7.9 | 41/2/0 | 2.7±1.4 | Denmark |
|  | CG=43(26) | 45.6±9.3 |  |  |  | 0 | 8.6±6 | 34/4/5 | 2.8±1.6 |  |
| Maryam K. Sokhangu 2021 | RT=10（10） | 38.7±7.24 | 8 | 3 | 60 | 1044 | 4.2±2.1 | NA | 1.75±0.71 | Iran |
|  | CG=10（10） | 40.1±5.64 |  |  |  | 0 | 4.4±2 | NA | 1.85±0.66 |  |
| Pelin Vural 2023 | COM=10(8) | 16.3±1.15 | 8 | 2 | 60 | 600 | 1.36±1.21 | 10/0/0 | 1.2±0.78 | Turkey |
|  | CG=10(9) | 17.4±1.57 |  |  |  | 0 | 2.26±1.69 | 10/0/0 | 1.65±0.81 |  |
| Peyman Pasha 2023 | HIIT=10(10) | 30.4±4.9 | 6 | 3 | 60 | 1260 | NA | 10/0/0 | 2.35±0.9 | Iran |
|  | AE=10(10) | 31.4±7.23 | 6 | 3 | 60 | 864 | NA | 10/0/0 | 2.7±0.6 |  |
|  | CG=10(10) | 32.2±4.39 |  |  |  | 0 | NA | 10/0/0 | 2.85±0.6 |  |
| S. Englund 2022 | RT=34(27) | 40.5±9.7 | 12 | 2 | 60 | 720 | 7.7±5.1 | 35/0/0 | 2±0.74 | Sweden |
|  | CG=64(49) | 43.6±10.3 |  |  |  | 0 | 8.6±5.6 | 64/0/5 | 2.5±1.48 |  |

#
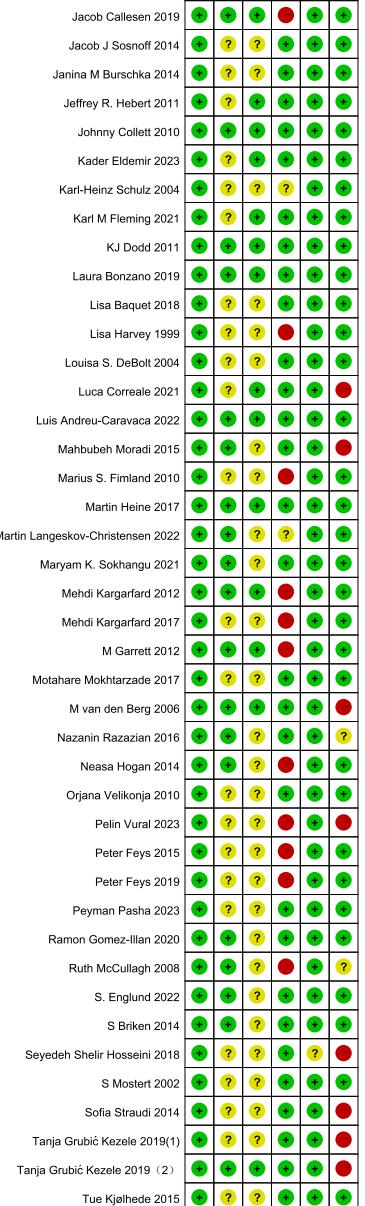
Risk of bias


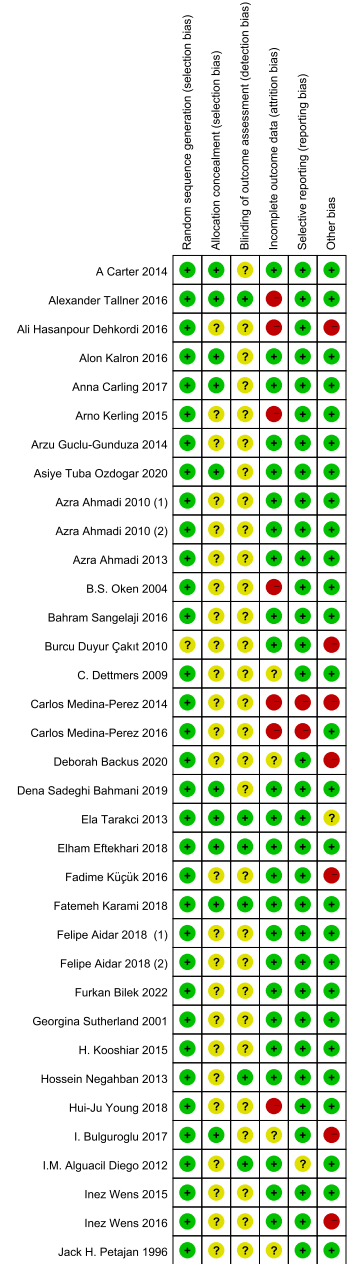


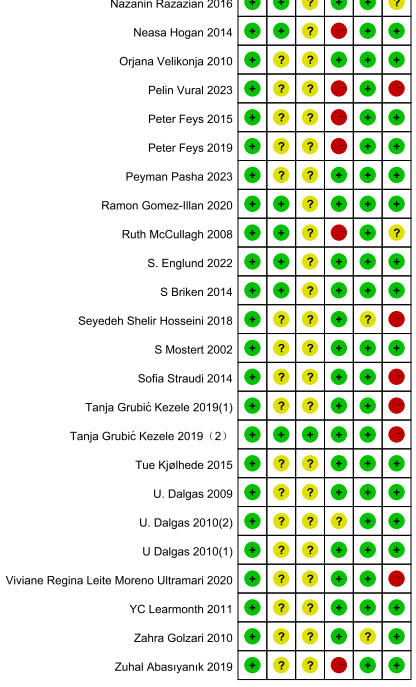


**Figure S1.** The detail of risk of bias


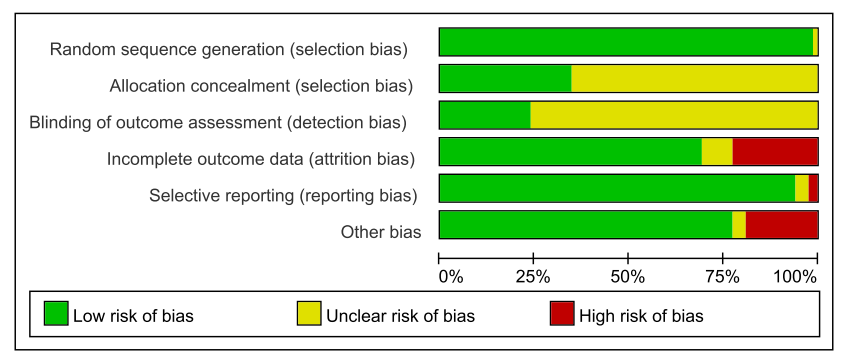


**Figure S2.** The summary of risk of bias

# Bayesian network meta-analysis

## 4.1 Model fit summaries for included studies

| **Model** | **Fatigue (127 data points)** | | | |
| --- | --- | --- | --- | --- |
|  | **DIC** | **pD** | **Residual**  **Deviance** | **SD (95%CrI)** |
| RE Model | 250.10 | 105.61 | 144.54 | 0.58  (0.42, 0.78) |
| RE UME | 251.71 | 109.31 | 142.39 | 0.62  (0.45, 0.84) |

**Table S2a.** Model fit summaries for included studies. (fatigue)

**Table S2b.** Model fit summaries for included studies. (muscle)

| **Model** | **Muscular fitness (105 data points)** | | | |
| --- | --- | --- | --- | --- |
|  | **DIC** | **pD** | **Residual**  **Deviance** | **SD (95%CrI)** |
| RE Model | 204.16 | 85.06 | 119.07 | 0.54  (0.34, 0.78) |
| RE UME | 206.79 | 88.39 | 118.40 | 0.57  (0.35, 0.83) |

***Note:*** CrI, credible interval. Abbreviations: DIC, deviance information criterion; pD, number of effective parameters; RE, random effects; UME, unrelated mean effects.

4.2 Deviance report for all studies

4.2.1 Residual deviance from NMA model and UME inconsistency model for all studies

This plot represents each data points' contribution to the residual deviance for the NMA with consistency (horizontal axis) and the unrelated mean effect (ume) inconsistency models (vertical axis) along with the line of equality. The points on the equality line means there is no improvement in model fit when using the inconsistency model, suggesting that there is no evidence of inconsistency. Points above the equality line means they have a smaller residual deviance for the consistency model indicating a better fit in the NMA consistency model and points below the equality line means they have a better fit in the ume inconsistency model. Please note that the unrelated mean effects model may not handle multi-arm trials correctly.


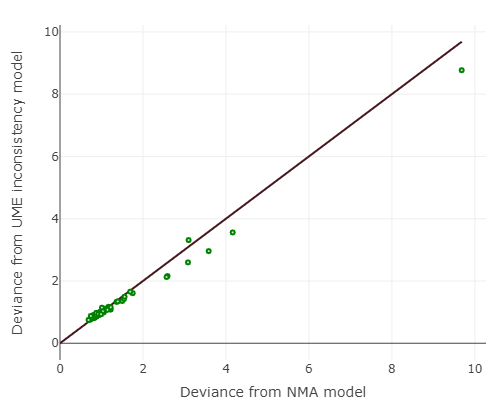


**Figure S3a**. Residual deviance from NMA model and UME inconsistency model for all studies. (fatigue)


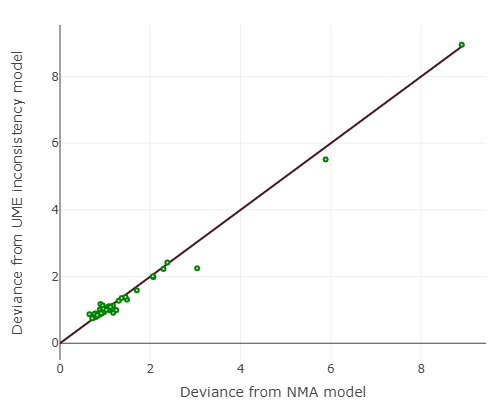


**Figure S3b**. Residual deviance from NMA model and UME inconsistency model for all studies. (muscle)

4.2.2 Per-arm residual deviance for all studies

This stem plot represents the posterior residual deviance per study arm. The total number of stems equals the total number of data points in the network meta-analysis. Going from left to right, the alternating symbols on the stems indicate the different studies. Each stem corresponds to the residual deviance associated with each arm in each study. The smaller residual deviance (the shorter stem), the better model fit for each data point.


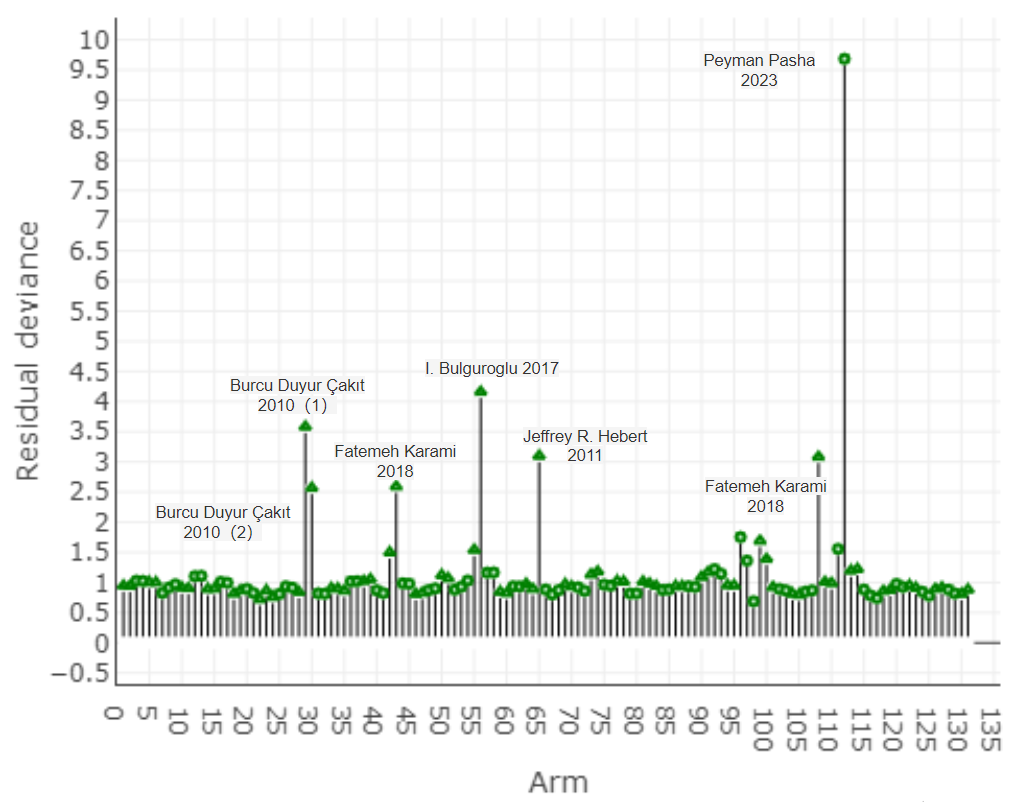


**Figure S4a.** Per-arm residual deviance for all studies. (fatigue)


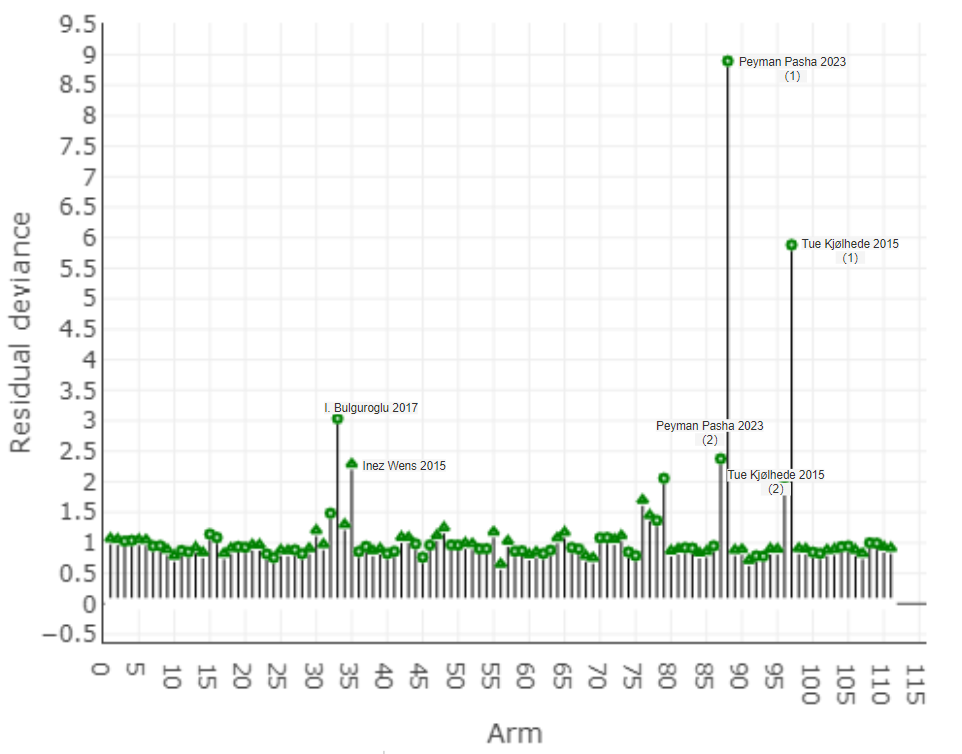


**Figure S4b.** Per-arm residual deviance for all studies. (muscle)

## 4.3 Node-split for all studies

**Table S3a.** Node-split for all studies (fatigue)

| **comparison** | **p.value** | **CrI** |
| --- | --- | --- |
| d.AE.CG | 0.974725 | NA |
| -> direct | NA | 0.49 (0.19, 0.79) |
| -> indirect | NA | 0.51 (-0.49, 1.5) |
| -> network | NA | 0.52 (0.25, 0.80) |
| d.AE.COM | 0.87305 | NA |
| -> direct | NA | -0.25 (-1.2, 0.75) |
| -> indirect | NA | -0.15 (-0.80, 0.49) |
| -> network | NA | -0.17 (-0.69, 0.34) |
| d.AE.HIIT | 0.286325 | NA |
| -> direct | NA | 0.54 (-0.53, 1.7) |
| -> indirect | NA | -0.25 (-1.2, 0.74) |
| -> network | NA | 0.020 (-0.65, 0.71) |
| d.AE.MBE | 0.5892 | NA |
| -> direct | NA | -0.27 (-0.93, 0.39) |
| -> indirect | NA | -0.50 (-1.0, 0.030) |
| -> network | NA | -0.41 (-0.82, -0.013) |
| d.AE.RT | 0.786 | NA |
| -> direct | NA | -0.44 (-1.2, 0.30) |
| -> indirect | NA | -0.33 (-0.76, 0.11) |
| -> network | NA | -0.34 (-0.72, 0.025) |
| d.CG.COM | 0.459275 | NA |
| -> direct | NA | -0.56 (-1.2, 0.053) |
| -> indirect | NA | -0.93 (-1.7, -0.14) |
| -> network | NA | -0.70 (-1.2, -0.22) |
| d.CG.HIIT | 0.81975 | NA |
| -> direct | NA | -0.68 (-1.4, 0.095) |
| -> indirect | NA | -0.52 (-1.7, 0.68) |
| -> network | NA | -0.50 (-1.1, 0.16) |
| d.CG.MBE | 0.810625 | NA |
| -> direct | NA | -0.99 (-1.4, -0.57) |
| -> indirect | NA | -0.87 (-1.8, 0.074) |
| -> network | NA | -0.93 (-1.3, -0.60) |
| d.CG.RT | 0.817025 | NA |
| -> direct | NA | -0.81 (-1.2, -0.48) |
| -> indirect | NA | -0.91 (-1.7, -0.11) |
| -> network | NA | -0.87 (-1.2, -0.58) |
| d.COM.HIIT | 0.87725 | NA |
| -> direct | NA | 0.32 (-1.1, 1.8) |
| -> indirect | NA | 0.19 (-0.74, 1.1) |
| -> network | NA | 0.19 (-0.56, 0.97) |
| d.COM.RT | 0.346925 | NA |
| -> direct | NA | 0.42 (-0.94, 1.8) |
| -> indirect | NA | -0.28 (-0.87, 0.31) |
| -> network | NA | -0.17 (-0.71, 0.36) |
| d.MBE.RT | 0.318425 | NA |
| -> direct | NA | -0.13 (-0.72, 0.46) |
| -> indirect | NA | 0.26 (-0.24, 0.76) |
| -> network | NA | 0.069 (-0.33, 0.47) |

**Table S3b.** Node-split for all studies (muscle)

| **Comparison** | **p.value** | **CrI** |
| --- | --- | --- |
| d.AE.CG | 0.17345 | NA |
| -> direct | NA | 0.24 (-0.21, 0.70) |
| -> indirect | NA | 0.86 (0.067, 1.7) |
| -> network | NA | 0.38 (-0.014, 0.80) |
| d.AE.COM | 0.314325 | NA |
| -> direct | NA | -0.024 (-0.97, 0.92) |
| -> indirect | NA | -0.60 (-1.3, 0.044) |
| -> network | NA | -0.42 (-0.96, 0.092) |
| d.AE.HIIT | 0.646225 | NA |
| -> direct | NA | -0.36 (-1.3, 0.48) |
| -> indirect | NA | -0.65 (-1.6, 0.30) |
| -> network | NA | -0.49 (-1.2, 0.14) |
| d.AE.MBE | 0.8278 | NA |
| -> direct | NA | -0.17 (-1.4, 1.1) |
| -> indirect | NA | -0.33 (-1.0, 0.37) |
| -> network | NA | -0.26 (-0.87, 0.34) |
| d.AE.RT | 0.199375 | NA |
| -> direct | NA | -1.3 (-3.0, 0.40) |
| -> indirect | NA | -0.14 (-0.65, 0.37) |
| -> network | NA | -0.23 (-0.71, 0.25) |
| d.CG.COM | 0.8832 | NA |
| -> direct | NA | -0.80 (-1.3, -0.32) |
| -> indirect | NA | -0.86 (-1.7, -0.068) |
| -> network | NA | -0.81 (-1.2, -0.41) |
| d.CG.HIIT | 0.116225 | NA |
| -> direct | NA | -1.3 (-2.2, -0.50) |
| -> indirect | NA | -0.32 (-1.3, 0.60) |
| -> network | NA | -0.87 (-1.5, -0.27) |
| d.CG.MBE | 0.74165 | NA |
| -> direct | NA | -0.68 (-1.4, -0.025) |
| -> indirect | NA | -0.50 (-1.4, 0.40) |
| -> network | NA | -0.65 (-1.2, -0.16) |
| d.CG.RT | 0.896625 | NA |
| -> direct | NA | -0.61 (-0.93, -0.31) |
| -> indirect | NA | -0.55 (-1.5, 0.31) |
| -> network | NA | -0.62 (-0.91, -0.35) |
| d.COM.HIIT | 0.69745 | NA |
| -> direct | NA | 0.11 (-1.3, 1.5) |
| -> indirect | NA | -0.20 (-1.1, 0.63) |
| -> network | NA | -0.066 (-0.78, 0.62) |
| d.COM.RT | 0.224775 | NA |
| -> direct | NA | 0.95 (-0.38, 2.3) |
| -> indirect | NA | 0.078 (-0.43, 0.60) |
| -> network | NA | 0.19 (-0.29, 0.67) |
| d.MBE.RT | 0.449175 | NA |
| -> direct | NA | -0.14 (-0.87, 0.59) |
| -> indirect | NA | 0.26 (-0.49, 1.0) |
| -> network | NA | 0.029 (-0.48, 0.54) |

## 4.4 Forest plot of all studies


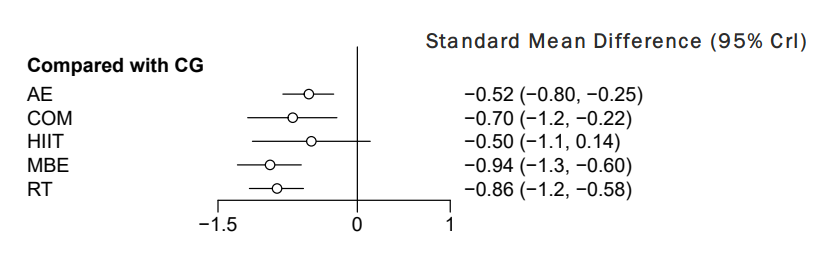


**Figure S5a.** Forest plot for all studies. (fatigue) HIIT High-intensity interval training, COM Combined exercise, RT Resistance training, AE Aerobic exercise, MBE Mind–body exercises. CG, control group.


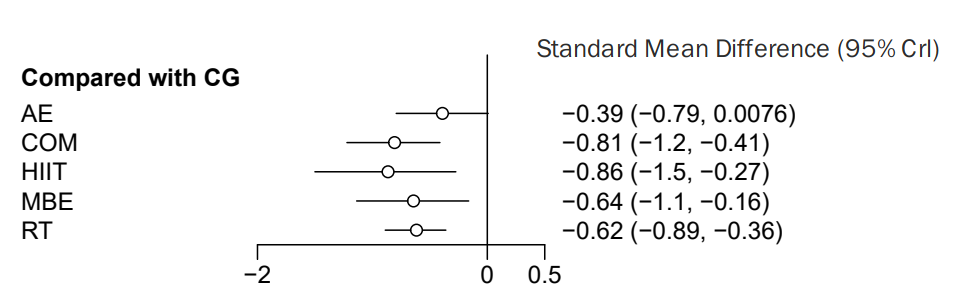


**Figure S5b.** Forest plot for all studies. (muscle) HIIT High-intensity interval training, COM Combined exercise, RT Resistance training, AE Aerobic exercise, MBE Mind–body exercises. CG, control group.

## 4.5 SUCRA table for all studies

**Table S4a.** SUCRA table for all studies (fatigue)

| Treatment | Rank 1 | Rank 2 | Rank 3 | Rank 4 | Rank 5 | Rank 6 | SUCRA |
| --- | --- | --- | --- | --- | --- | --- | --- |
| AE | 0.002188 | 0.017538 | 0.1608 | 0.447825 | 0.371563 | 8.75E-05 | 36.614 |
| CG | 0 | 0 | 0 | 0.00055 | 0.066813 | 0.932638 | 1.35825 |
| COM | 0.134538 | 0.158888 | 0.339288 | 0.231238 | 0.13385 | 0.0022 | 58.4485 |
| HIIT | 0.070563 | 0.074813 | 0.150375 | 0.225288 | 0.413888 | 0.065075 | 39.353 |
| MBE | 0.52545 | 0.297688 | 0.133688 | 0.036975 | 0.0062 | 0 | 85.98425 |
| RT | 0.267263 | 0.451075 | 0.21585 | 0.058125 | 0.007688 | 0 | 78.242 |

**Table S4b.** SUCRA table for all studies (muscle)

| Treatment | Rank 1 | Rank 2 | Rank 3 | Rank 4 | Rank 5 | Rank 6 | SUCRA |
| --- | --- | --- | --- | --- | --- | --- | --- |
| AE | 0.0039 | 0.024725 | 0.089975 | 0.209325 | 0.643088 | 0.028988 | 29.00125 |
| CG | 0 | 0 | 0 | 7.00E-04 | 0.036013 | 0.963288 | 0.74825 |
| COM | 0.314025 | 0.374788 | 0.181688 | 0.103 | 0.026463 | 3.75E-05 | 76.936 |
| HIIT | 0.494338 | 0.225963 | 0.125013 | 0.110163 | 0.042113 | 0.002413 | 80.26025 |
| MBE | 0.1404 | 0.203313 | 0.249825 | 0.249738 | 0.151475 | 0.00525 | 58.3135 |
| RT | 0.047338 | 0.171213 | 0.3535 | 0.327075 | 0.10085 | 2.50E-05 | 54.74075 |

***Note:*** HIIT High-intensity interval training, COM Combined exercise, RT Resistance training, AE Aerobic exercise, MBE Mind–body exercises. CG, control group.

## 4.6 GRADE Summary of Findings (SoFs) tables

We determined the quality of evidence using the Grading of Recommendations Assessment, Development, and Evaluation (GRADE) method(1). The GRADE classification is downgraded one level from high certainty when the following occurs (Figure 1):

1: Inconsistency: in pairwise analyses, point estimates varied considerably across trials, or there was a high degree of heterogeneity between trials. In the NMA, there was a significant difference between the estimates for direct and indirect comparisons (incoherence).

2: Indirectness: was assessed by determining whether the study population, type of intervention, and results were directly relevant to the purpose of this meta-analysis.

3: Risk of bias: in any comparison, more than 50% of the included trials had “some concerns” or “high risk of bias”.

4.Intransitivity: in processing the quality grading of indirect evidence, there were significant differences between groups in terms of baseline population characteristics, common controls, and outcome measures.

5.Imprecision: based on an inspection of the 95% CI of the pooled estimates to determine if values with different clinical impacts were included; the sample size was insufficient. If both were present, the imprecision was downgraded by two levels.

6.Publication bias: based on evidence of the presence or absence of publication bias, such as small trials with mostly positive results, industry sponsorship, or reported conflicts of interest. We did not use funnel plots to determine publication bias because visual assessment of funnel plots is prone to error.

**
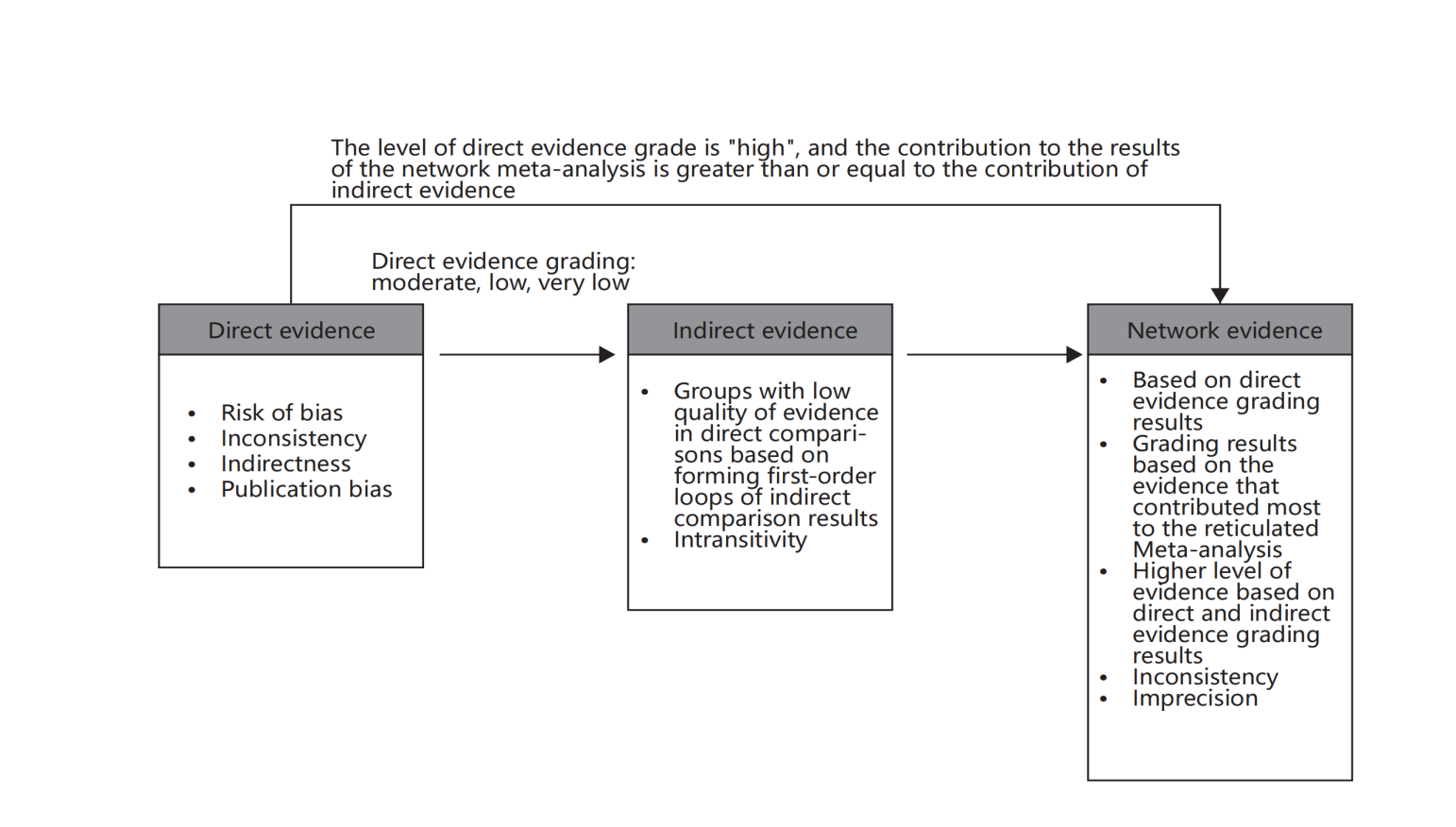
**

| Effect estimates, credible intervals, and certainty of evidence for comparisons of different Exercise interventions in patients with Multiple sclerosis. | |
| --- | --- |
| **Populations:** Patients with Multiple sclerosis  **Interventions:** Any modality of exercise  **Comparator (reference):** Any modality of non-exercise intervention, such as usual care, keep daily, educational counseling, waiting list; or placebo-therapy.  **Outcome:** FSS (Fatigue Severity Scale); FSMC (Fatigue Scale for Motor and Cognitive Functions); MFIS (Modified Fatigue Impact Scale); FIS (Fatigue Impact Scale); Weimus (Würzburg Fatigue Scale for Multiple Sclerosis); PROMIS (Patient-Reported Outcomes Measurement Information System); POMS (Profile of Mood States)  **Study design:** Randomized controlled trials | 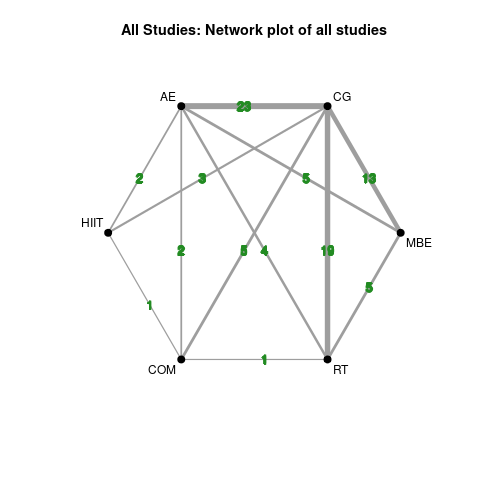  *Geometry of the Network (fatigue) |
| **P Populations:** Patients with Multiple sclerosis  **Interventions:** Any modality of exercise  **Comparator (reference):** Any modality of non-exercise intervention, such as usual care, keep daily, educational counseling, waiting list; or placebo-therapy.  **Outcome:** STS (Sit-to-Stand Test); CAR (Central Activation Ratio); CUT (Curl Up Test); Muscle fiber CSA (Muscle Fiber Cross-sectional Area); Strength MVC (Maximal Voluntary Contraction); MIT (Maximal Isometric Torque); Power MIVC (Maximum Voluntary Isometric Contraction); MPT (Modified Push-ups Test); MSL (Maximum Squat Load); Standing Test WST (Wall Squat Test); SCT (Stair-Climbing Test)  ; MWD (Maximal Walking Distance); PHT (Plank-Hold Test)  **Study design:** Randomized controlled trials | 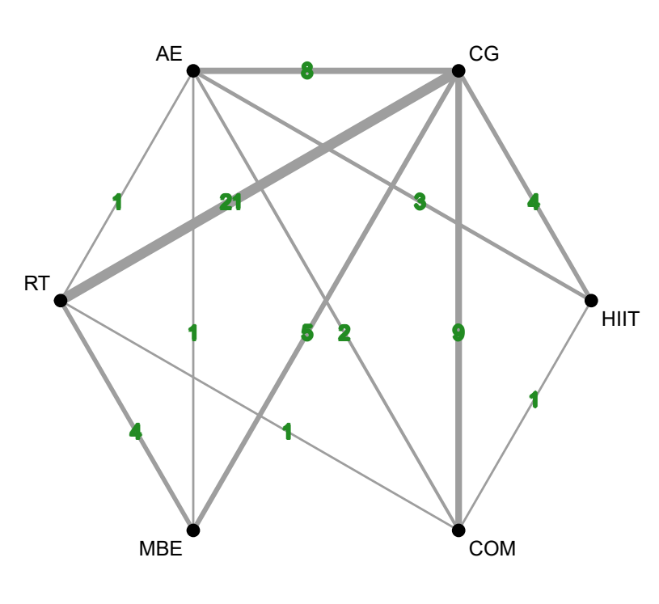  *Geometry of the Network (muscle) |

Presentation and interpretation of findings of our Network Meta-Analysis (NMA) through the GRADE

system for NMA: Fatigue and muscle.

**Table S8.** Grade for all studies.

| **Fatigue** | | | | | | | **P** |
| --- | --- | --- | --- | --- | --- | --- | --- |
| **Comparison** | **Direct Evidence** | | **Indirect Evidence** | | **Network Meta-Analysis** | |  |
|  | **SMD (95% CrI)** | **Certainty of Evidence** | **SMD (95% CrI)** | **Certainty of Evidence** | **SMD (95% CrI)** | **Certainty of Evidence** |  |
| AE vs. CG | 0.49 (0.20, 0.79) | Moderate^1^ | 0.50(-0.50, 1.5) | Low^1,3^ | 0.52 (0.25, 0.80) | Very Low^1,3,5^ | 0.978 |
| AE vs. COM | -0.25 (-1.2, 0.74) | Low^1,3^ | -0.15 (-0.80, 0.49) | Low^1,3^ | -0.17 (-0.70, 0.34) | Very Low^1,3,5^ | 0.870 |
| AE vs. HIIT | 0.54 (-0.53, 1.6) | Low*^1^ | -0.25 (-1.2, 0.75) | Low^1,3^ | 0.023 (-0.64, 0.71) | Very Low^1,3,5^ | 0.282 |
| AE vs. MBE | -0.27 (-0.93, 0.3) | Moderate^1^ | -0.50(-1.0, 0.034) | Moderate^1^ | -0.42 (-0.83, 0.016) | Moderate^5^ | 0.593 |
| AE vs. RT | -0.44 (-1.2, 0.29) | Moderate^1^ | -0.32 (-0.76, 0.11) | Low^1,3^ | -0.35 (-0.73, 0.023) | Very Low^1,3,5^ | 0.777 |
| CG vs. COM | -0.56 (-1.2, 0.051) | Low^1,3^ | -0.93 (-1.7, -0.14) | Low^1,3^ | -0.69 (-1.2, -0.22) | Low^1,3^ | 0.461 |
| CG vs. HIIT | -0.67 (-1.4, 0.089) | Moderate^1^ | -0.51 (-1.7, 0.68) | Moderate^1^ | -0.50 (-1.1, 0.16) | Moderate^5^ | 0.821 |
| CG vs. MBE | -0.99 (-1.4, -0.58) | Low^1,3^ | -0.87 (-1.8, 0.061) | Low^1*^ | -0.94 (-1.3, -0.60) | Low^1,3^ | 0.821 |
| CG vs. RT | -0.81 (-1.2, -0.48) | Moderate^1^ | -0.91 (-1.7, -0.11) | Low^1,3^ | -0.87 (-1.2, -0.58) | Low^1,5^ | 0.817 |
| COM vs. HIIT | 0.34 (-1.1, 1.8) | Low*^1^ | 0.19 (-0.73, 1.1) | Low^1,3^ | 0.2 (-0.55, 0.98) | Very Low^1,3,5^ | 0.875 |
| COM vs. RT | 0.41 (-0.95, 1.8) | Moderate^1^ | -0.28 (-0.88, 0.30) | Low^1,3^ | -0.17 (-0.72, 0.37) | Very Low^1,3,5^ | 0.353 |
| COM vs. MBE |  |  |  |  | -0.24 (-0.82, 0.34) | Very Low^1,3,5^ |  |
| MBE vs. RT | -0.13 (-0.72, 0.46) | Low^1,3^ | 0.26 (-0.26, 0.77) | Low^1,3^ | 0.069 (-0.33, 0.47) | Low^1,3^ | 0.327 |
| MBE vs. HIIT |  |  |  |  | 0.44 (-0.27, 1.2) | Very Low^1,3,5^ |  |
| RT vs. HIIT |  |  |  |  | 0.37 (-0.32, 1.1) | Very Low^1,3,5^ |  |

| **Muscle** | | | | | | | **P** |
| --- | --- | --- | --- | --- | --- | --- | --- |
| **Comparison** | **Direct Evidence** | | **Indirect Evidence** | | **Network Meta-Analysis** | |  |
|  | **SMD (95% CrI)** | **Certainty of Evidence** | **SMD (95% CrI)** | **Certainty of Evidence** | **SMD (95% CrI)** | **Certainty of Evidence** |  |
| AE vs. CG | 0.24 (0.21, 0.70) | Moderate^1^ | 0.87(0.075, 1.7) | Low^1,3^ | 0.39 (-0.02, 0.80) | Low^1,3^ | 0.173 |
| AE vs. COM | -0.028 (-0.97, 0.92) | Low^1,3^ | -0.6 (-1.3, 0.036) | Low^1,3^ | -0.42 (-0.95, 0.095) | Low^1,3^ | 0.317 |
| AE vs. HIIT | -0.36 (-1.3, 0.47) | Low^1,3^ | -0.65 (-1.6, 0.31) | Low^1,3^ | -0.48 (-1.2, 0.14) | Very Low^1,3,5^ | 0.651 |
| AE vs. MBE | -0.16 (-1.4, 1.1) | Moderate^1^ | -0.33 (-1.0, 0.37) | Low^1,3^ | -0.26 (-0.89, 0.53) | Low^1,3^ | 0.812 |
| AE vs. RT | -1.3 (-3.0, 0.41) | Moderate^1^ | -0.13 (-0.64, 0.37) | Moderate^1^ | -0.23 (-0.73, 0.24) | Low^1, 5^ | 0.201 |
| CG vs. COM | -0.79 (-1.3, -0.31) | Moderate^1^ | -0.86 (-1.7, -0.059) | Low^1,3^ | -0.81 (-1.2, -0.40) | Low^1,3^ | 0.879 |
| CG vs. HIIT | -1.3 (-2.2, -0.50) | Low^1,3^ | -0.32(-1.3, 0.61) | Low^1,3^ | -0.87 (-1.5, -0.27) | Low^1,5^ | 0.111 |
| CG vs. MBE | -0.68 (-1.4, -0.025) | Moderate^1^ | -0.50 (-1.4, 0.40) | Low^1*^ | -0.65 (-1.2, -0.16) | Low^1,5^ | 0.751 |
| CG vs. RT | -0.61 (-0.93, -0.31) | Moderate^1^ | -0.56 (-1.5, 0.30) | Low^1,3^ | -0.62 (-0.91, -0.34) | Low^1,3^ | 0.922 |
| COM vs. HIIT | 0.11 (-1.3, 1.5) | Low*^1^ | -0.20 (-1.1, 0.63) | Low^1,3^ | -0.067 (-0.79, 0.62) | Very Low^1,3,5^ | 0.697 |
| COM vs. RT | 0.95 (-0.39, 2.3) | Moderate^1^ | 0.078 (-0.43, 0.59) | Low^1,3^ | 0.19 (-0.29, 0.67) | Very Low^1,3,5^ | 0.226 |
| COM vs. MBE |  |  |  |  | 0.17 (-0.45, 0.81) | Very Low^1,3,5^ |  |
| MBE vs. RT | -0.14 (-0.87, 0.59) | Low^1,3^ | 0.26 (-0.49, 1.0) | Low^1,3^ | 0.025 (-0.48, 0.54) | Low^1,3^ | 0.449 |
| MBE vs. HIIT |  |  |  |  | -0.23 (-1.03, 0.54) | Very Low^1,3,5^ |  |
| RT vs. HIIT |  |  |  |  | -0.25 (-0.95, 0.42) | Very Low^1,3,5^ |  |

***Note:**** represents a two-level downgrade, such as 1* represents a severe inconsistency. HIIT High-intensity interval training, COM Combined exercise, RT Resistance training, AE Aerobic exercise, MBE Mind–body exercises.

# Dose-response network meta-analyses

## 5.1 Key assumptions for Network Meta-Analysis

5.1.1 Network Connectivity

Connectivity is a key assumption in NMA that, if considered insufficient (i.e., due to lack of direct comparisons), may lead to low statistical power and misleading results 2. Our study assessed network connectivity at the motor and dose levels and did not find any evidence of network unconnectedness (Figure S9 and Figure S10)

1. Fatigue


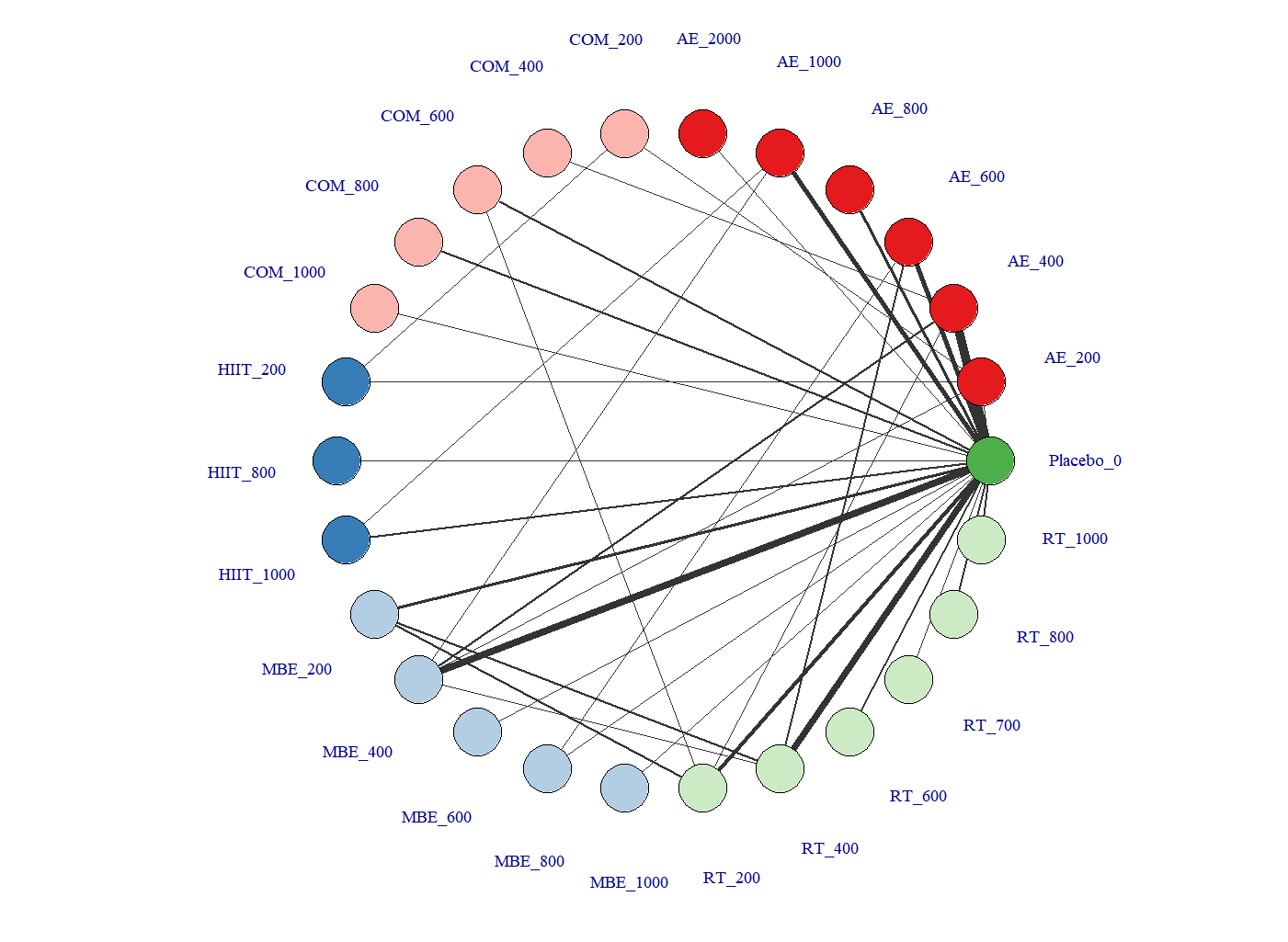


1. Muscular fitness


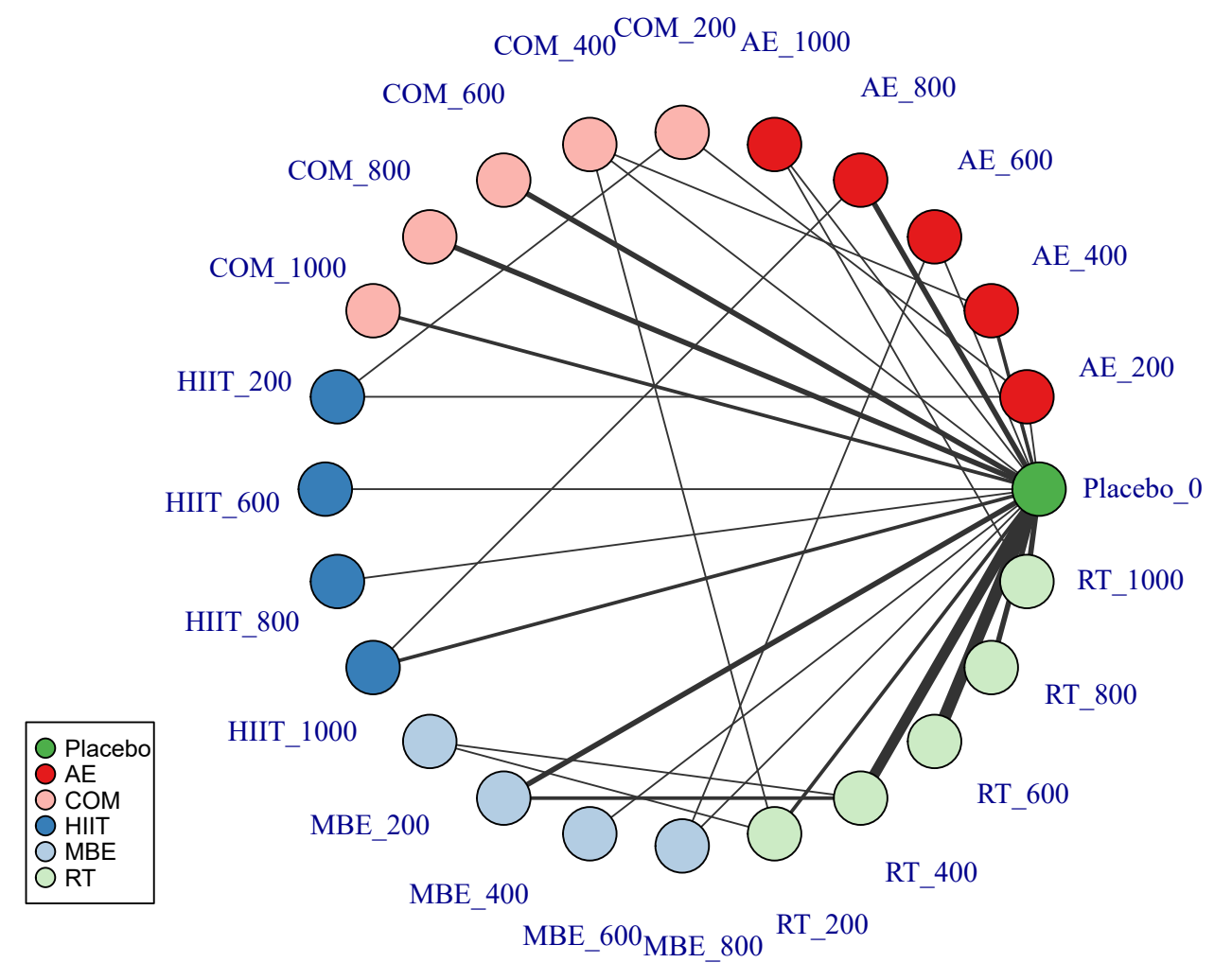


**Figure S6.** Treatment-level network. The first value indicates the specific intervention and the second one is the corresponding dose of that intervention. HIIT High-intensity interval training, COM Combined exercise, RT Resistance training, AE Aerobic exercise, MBE Mind–body exercises.

CG, control group.

1. fatigue


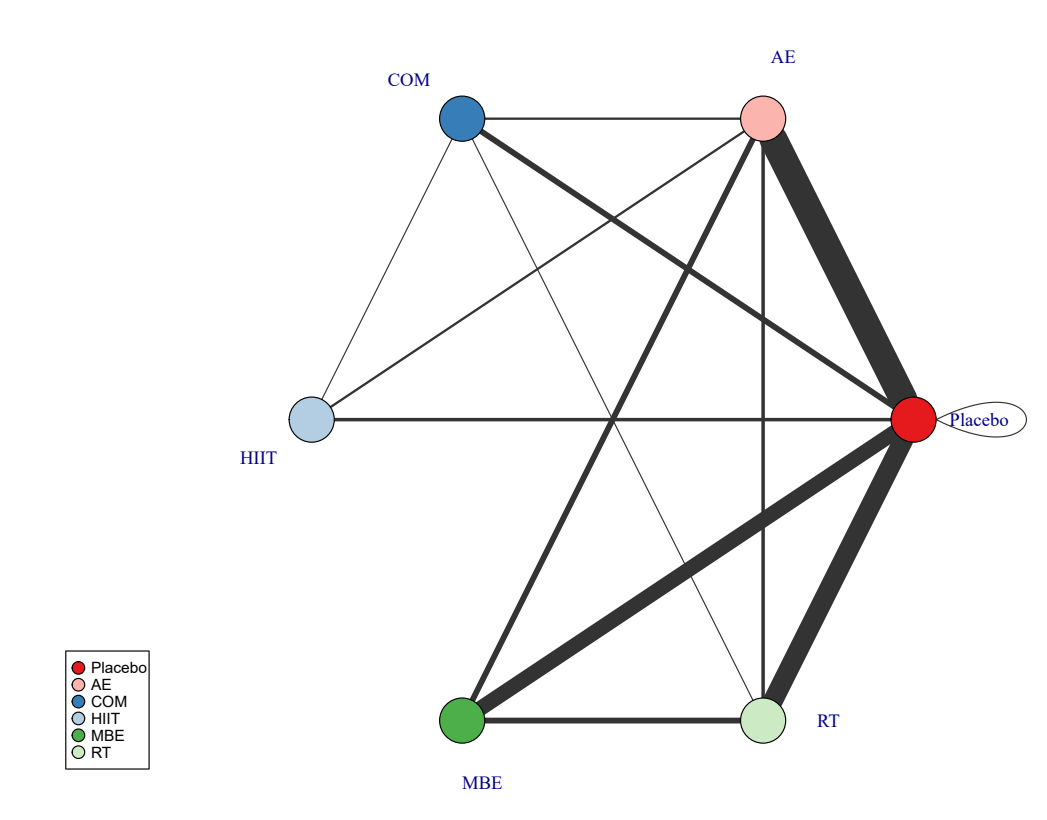


1. muscular fitness


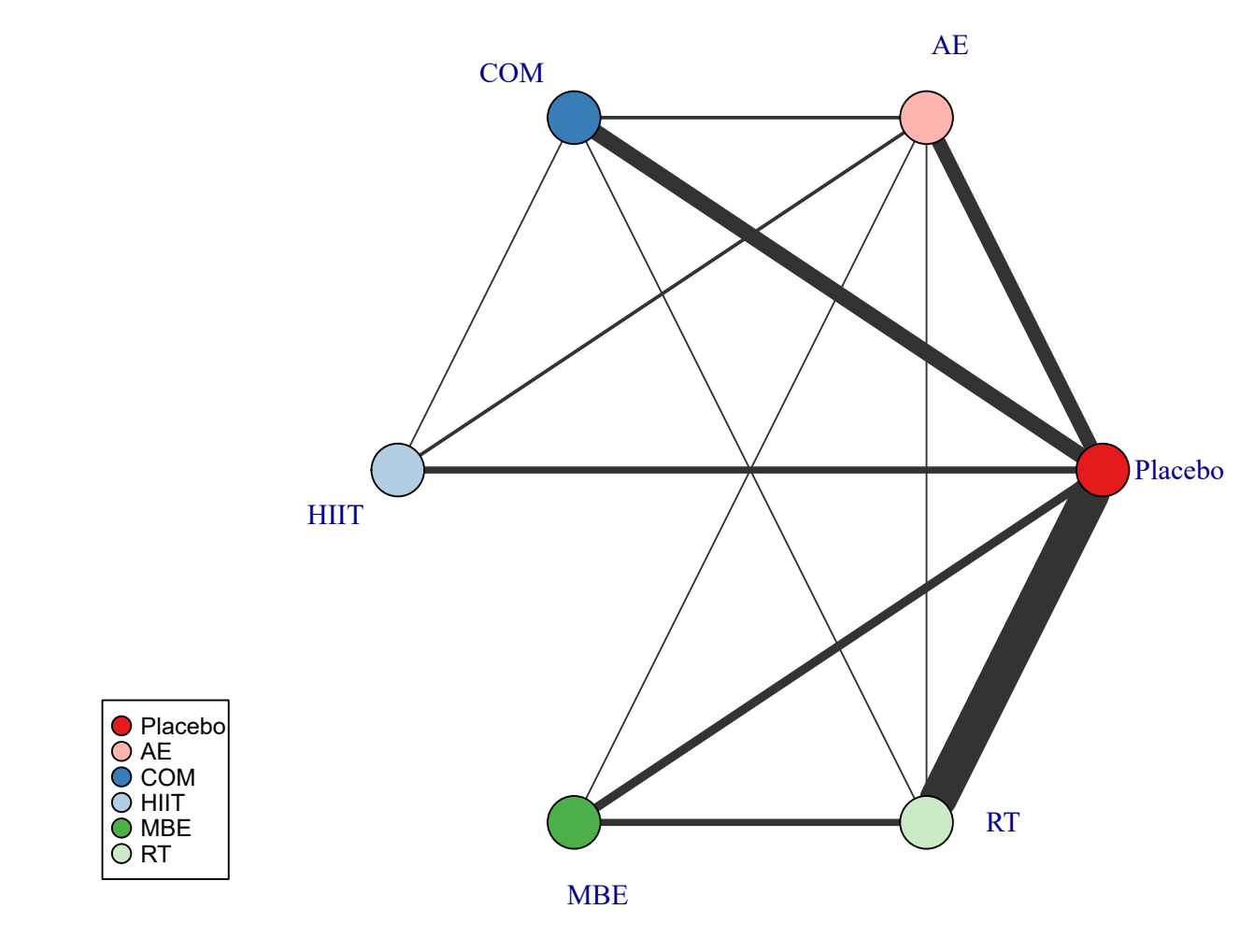


**Figure S7.** Agent-level network. The first value indicates the specific intervention and the second one is the corresponding dose of that intervention. HIIT High-intensity interval training, COM Combined exercise, RT Resistance training, AE Aerobic exercise, MBE Mind–body exercises.

CG, control group.

5.1.2 Data consistency

We performed a consistency analysis of the data by comparing the consistency of the network (i.e., network effect size) with the unrelated mean effects (UME) model (i.e., pairwise effect sizes). In practice, we checked whether the bias, the number of estimated parameters in the network, and the Deviance Information Criterion (DIC) metric were similar for both models, indicating a good fit3. The comparison of these parameters showed a good agreement between the different models (**Table** S9).

**Table S5a.** Consistent and UME models fit comparison. (fatigue)

| **Model** | **pD** | **Deviance** | **Residual deviance** | **DIC** | **SD** |
| --- | --- | --- | --- | --- | --- |
| Consistent | 114.6 | 19.943 | 115.099 | 133.5 | 6.279 |
| UME | 114.5 | 19.832 | 114.988 | 133.4 | 6.275 |

**Table S5b.** Consistent and UME models fit comparison (muscle)

| **Model** | **pD** | **Deviance** | **Residual deviance** | **DIC** | **SD** |
| --- | --- | --- | --- | --- | --- |
| Consistent | 77.5 | 58.413 | 106.266 | 135.4 | 0.584 |
| UME | 76.2 | 60.696 | 108.548 | 135.9 | 0.577 |

5.1.3 Network transitivity

NMAs are based on the assumption of indirect/mixed comparisons, which implies that estimates of treatment effects from direct and indirect evidence are consistent but with the usual variation of meta-analyses under a random effects model 4. This assumption is equivalent to heterogeneity in a "standard" meta-analysis 5. Following a previous proposal 6, anomalies were assessed at a deeper network level (i.e., at the treatment level). We assessed the span by the MBNMA node-splitting method. This method splits the contribution of a specific treatment contrast into direct and indirect evidence and compares them 7. Similar effects indicate good span. Table S10 and Figure S11 presents the results for transitivity in this meta-analysis.

**Table S6a.** Node-splitting analysis of consistency (fatigue)


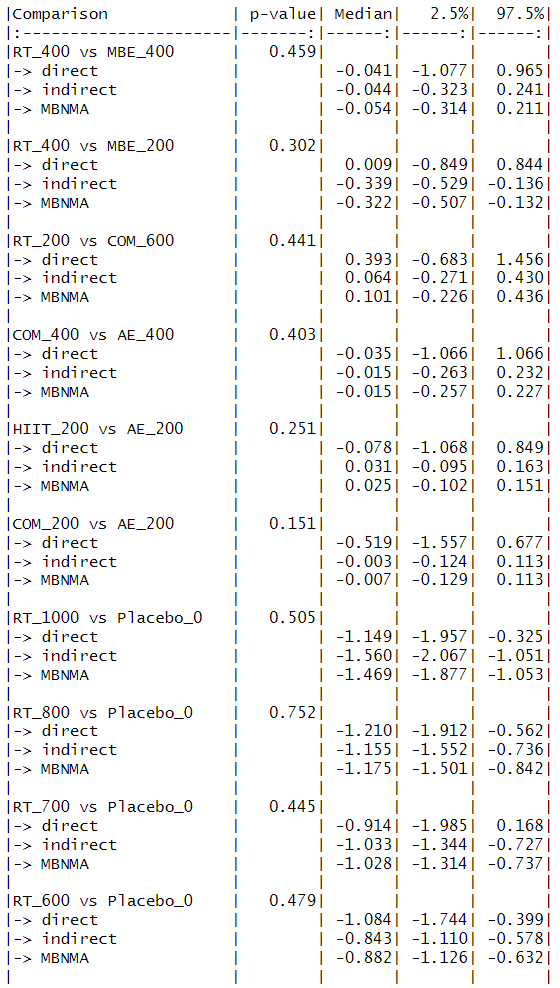


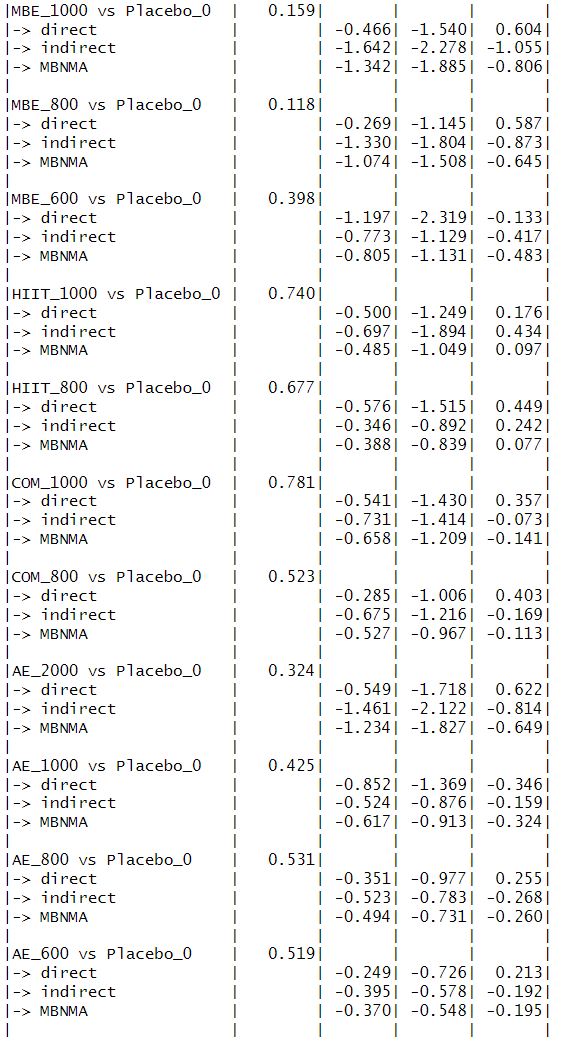


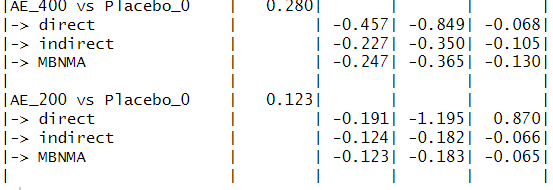


**Table S6b.** Node-splitting analysis of consistency (muscle)

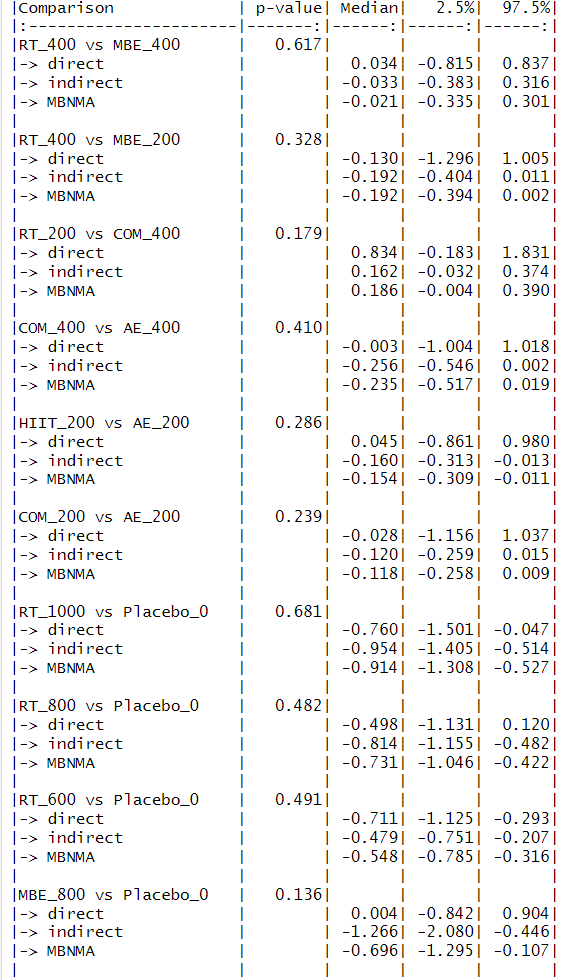


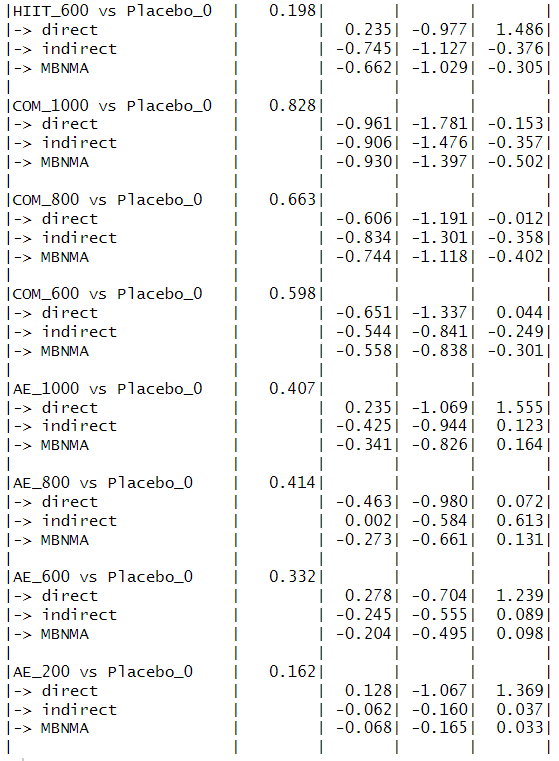


***Note:*** CrI, credible interval. HIIT High-intensity interval training, COM Combined exercise, RT Resistance training, AE Aerobic exercise, MBE Mind–body exercises. CG, control group.


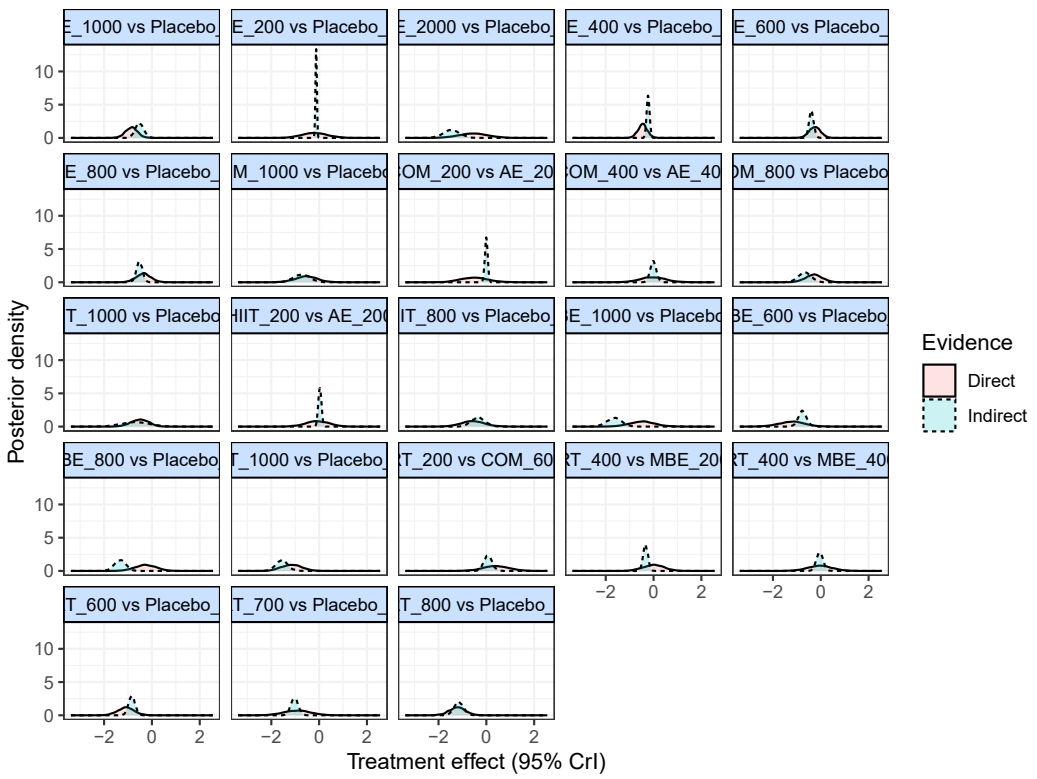


**Figure S8a.** Node-splitting analysis (density plot; fatigue). The value of title is the corresponding dose of that agent. CrI, credible interval. HIIT High-intensity interval training, COM Combined exercise, RT Resistance training, AE Aerobic exercise, MBE Mind–body exercises. CG, control group.


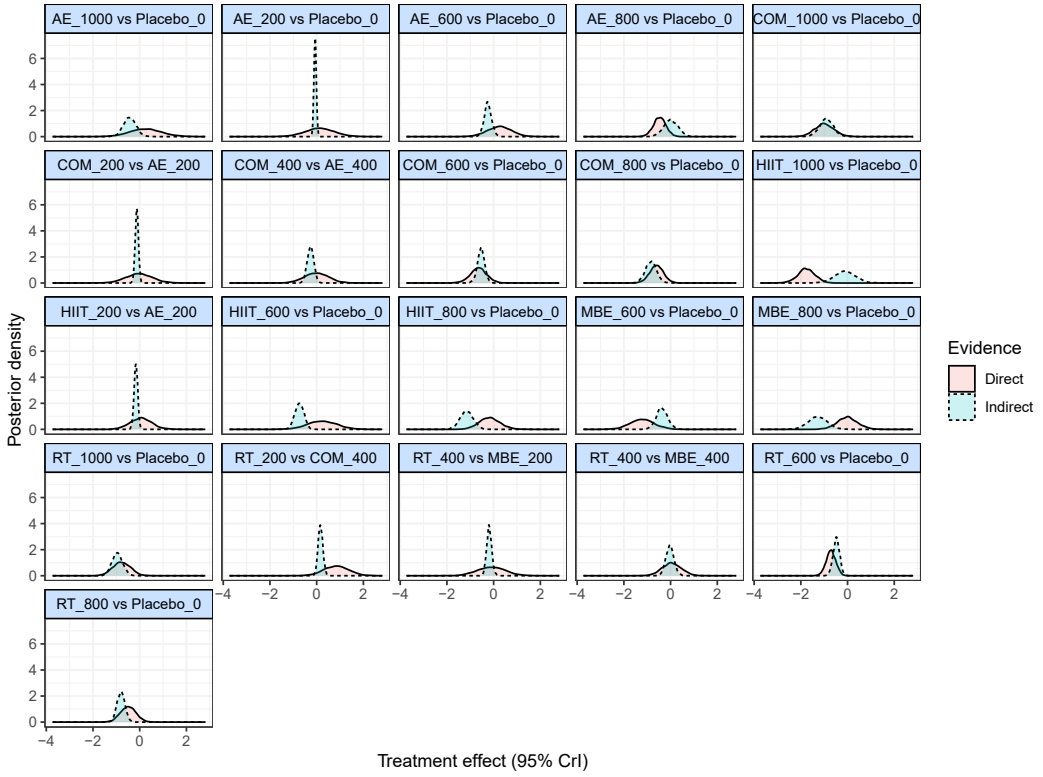


**Figure S8b.** Node-splitting analysis (density plot; muscle). The value of title is the corresponding dose of that agent. CrI, credible interval. HIIT High-intensity interval training, COM Combined exercise, RT Resistance training, AE Aerobic exercise, MBE Mind–body exercises. CG, control group.

## 5.2 Models’ selection

5.2.1 Non-linear functions and models fit comparison

A meta-analysis (i.e., a "split" NMA) of the different doses of physical activity as separate and unrelated treatments were performed. This step helps determine which function is more appropriate for the data and should be used in a model-based network meta-analysis (MBNMA) 9. **Figure S12** and **Figure S13** show the different responses of each dose to overall and different types of exercise, respectively (SMD).


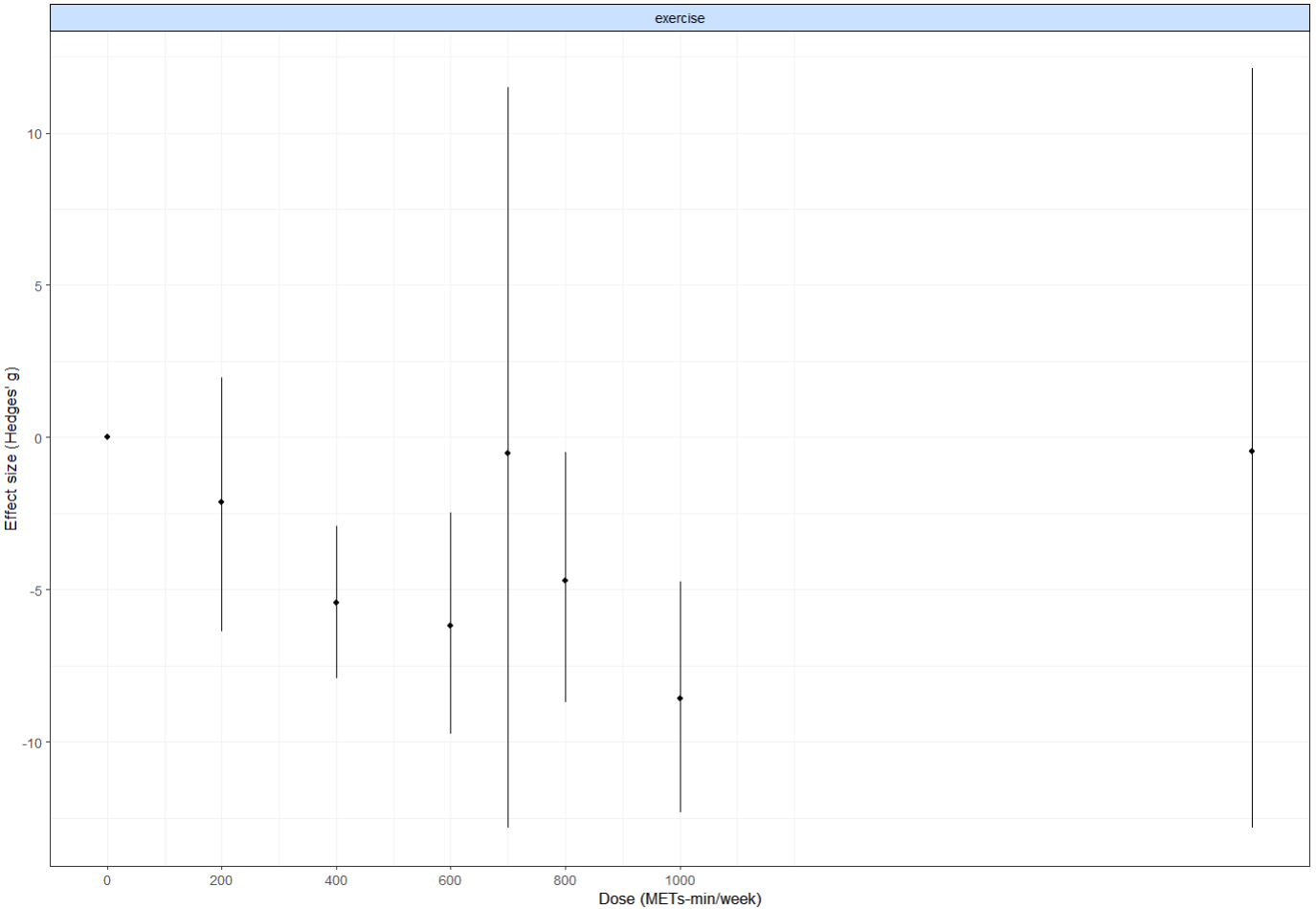


**Figure S9a.**“Split” NMA of overall exercise. (fatigue)


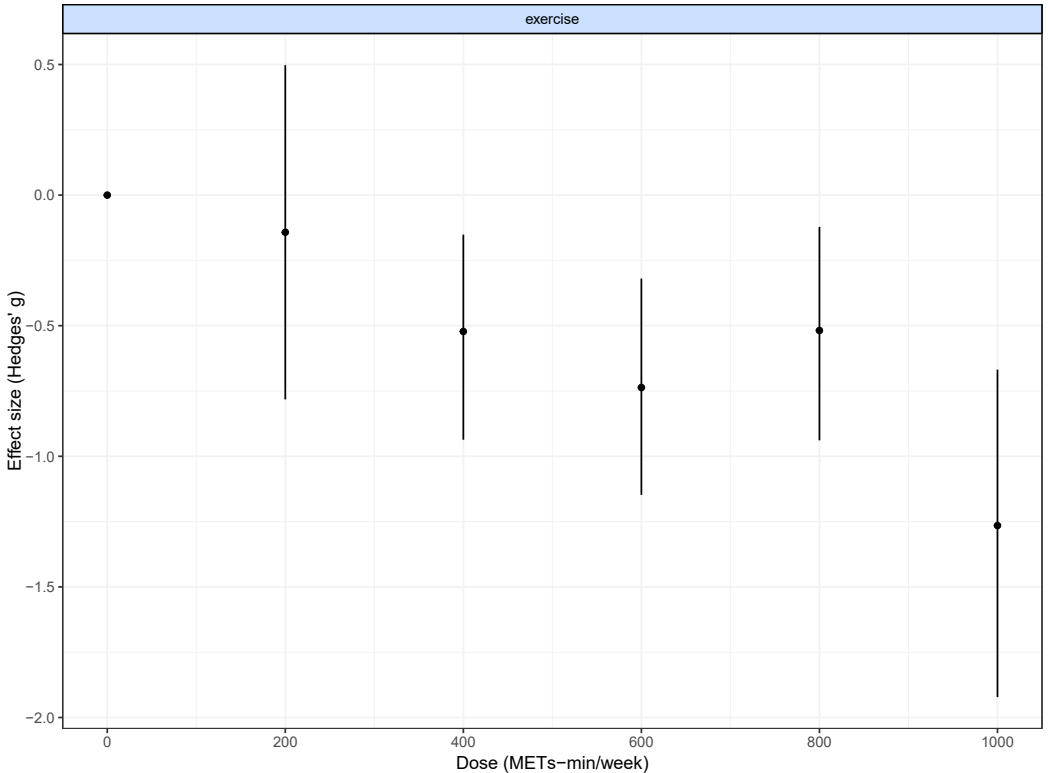


**Figure S9b.**“Split” NMA of overall exercise. (muscle)


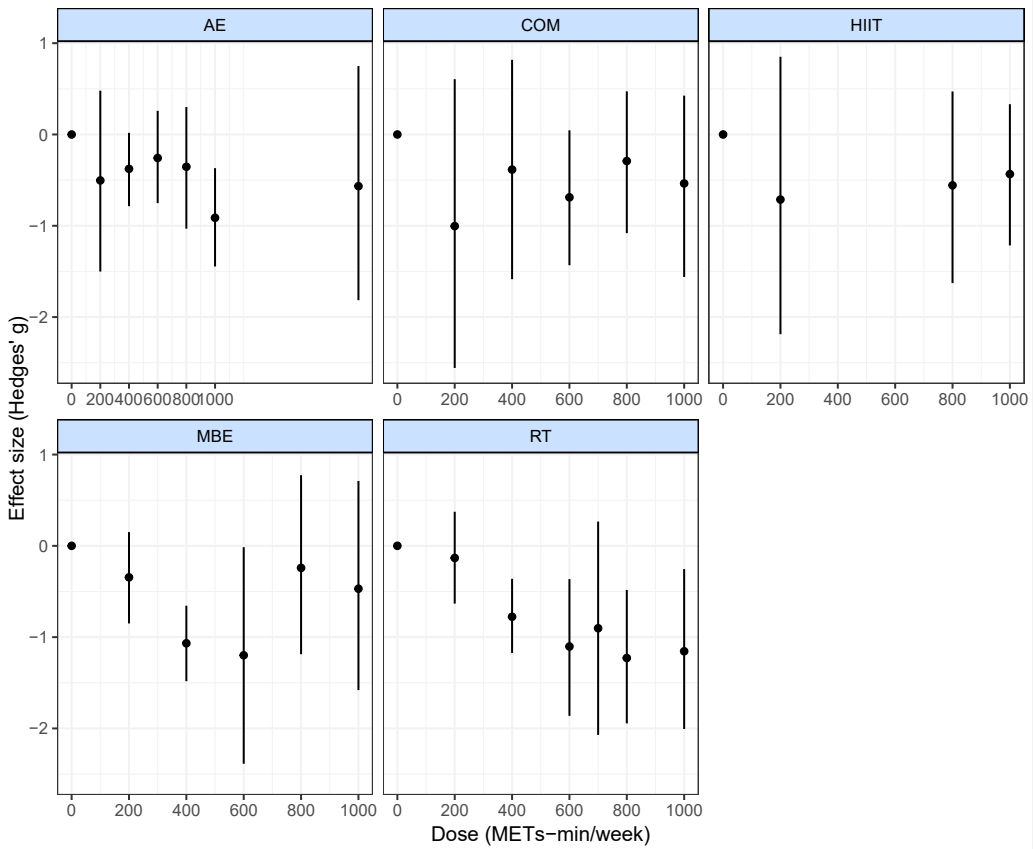


**Figure S10a.** “Split” NMA of different exercise agents. HIIT High-intensity interval training, COM Combined exercise, RT Resistance training, AE Aerobic exercise, MBE Mind–body exercises. CG, control group. (fatigue)


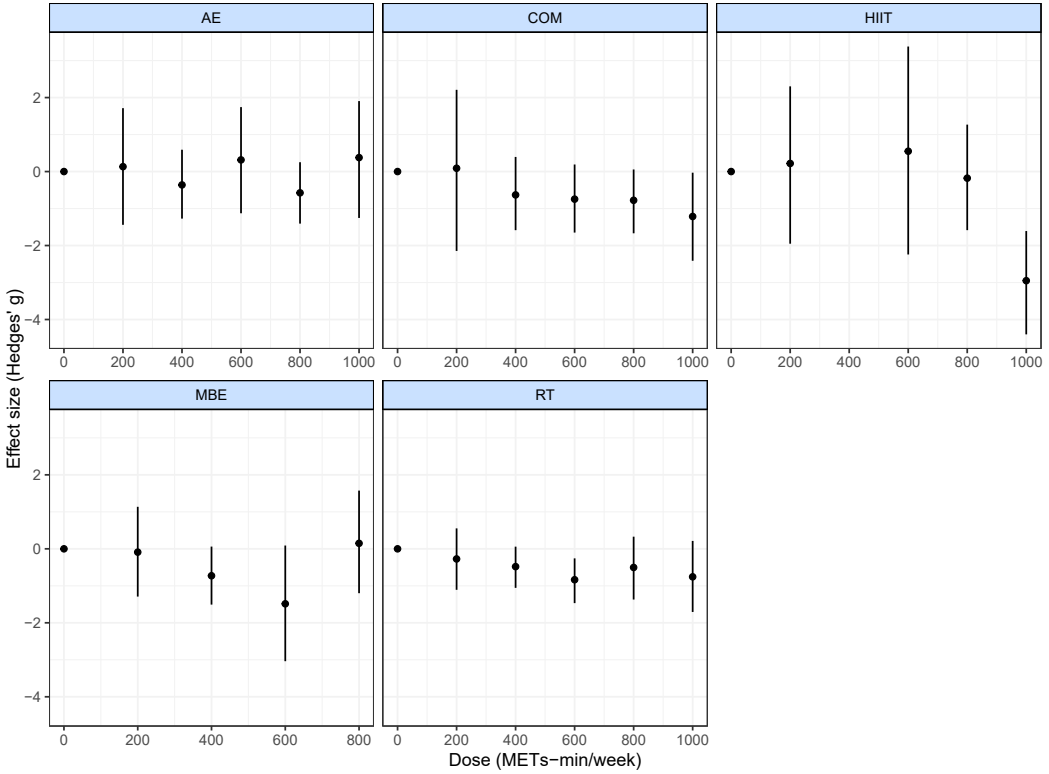


**Figure S10b.** “Split” NMA of different exercise agents. HIIT High-intensity interval training, COM Combined exercise, RT Resistance training, AE Aerobic exercise, MBE Mind–body exercises. CG, control group. (muscle)

5.2.2 Models selection

For our data, quadratic model shows the best fit and were therefore used in subsequent analyses.

**Table S7a.** Models fit comparison (fatigue)

| **Model** | **DIC** | **SD** | **Deviance** | **Residual deviance** | **pD** |
| --- | --- | --- | --- | --- | --- |
| Emax (common treatment effects) | 19350.4 | NA | 19295.91 | 19391.066 | 55.1 |
| Restricted cubic spline (common treatment effects; 3 knots) | 18715.4 | NA | 18659.01 | 18754.174 | 57.4 |
| Restricted cubic spline (random treatment effects; 3 knots) | 133.2 | 6.181  (5.167  ,7.477) | 19.682 | 114.838 | 114.2 |
| Non-parametric monotonically up (common treatment effects) | 36988.5 | NA | 36935.990 | 37031.146 | 53.0 |
| Quadratic (2^nd^ degree polynomial, common treatment effects) | 19302.7 | NA | 19248.141 | 19343.297 | 54.9 |
| Quadratic (2^nd^ degree polynomial, random treatment effects) | 134.0 | 6.158  (5.172  ,7.392) | 19.924 | 115.079 | 115.1 |

**Table S7b.** Models fit comparison (muscle)

| **Model** | **DIC** | **SD** | **Deviance** | **Residual deviance** | **pD** |
| --- | --- | --- | --- | --- | --- |
| Emax (common treatment effects) | 176.8 | NA | 131.353 | 179.205 | 46.2 |
| Restricted cubic spline (common treatment effects; 3 knots) | 169.2 | NA | 120.953 | 168.805 | 48.8 |
| Restricted cubic spline (random treatment effects; 3 knots) | 134.7 | 0.551  (0.331  , 0.813) | 60.499 | 108.351 | 75.4 |
| Non-parametric monotonically up (common treatment effects) | 254.7 | NA | 209.776 | 257.628 | 45.6 |
| Quadratic (2^nd^ degree polynomial, common treatment effects) | 175.6 | NA | 128.936 | 176.788 | 47.3 |
| Quadratic (2^nd^ degree polynomial, random treatment effects) | 135.6 | 0.576  (0.368  , 0.819) | 60.271 | 108.124 | 75.7 |

In addition to the model fit index, a deviation plot showing the contribution of each data point to the residuals can also help to confirm the robustness of the model selection. The contribution of each data point to the posterior mean bias should be around 1, which indicates a good model fit(2). Deviation plots for overall **(Figure S11)** treatment effects **(Figure S12)** confirm the robustness of our model selection (i.e., deviations <1.5 except for a few data points in the overall exercise and RT at 700 METs-min, which are all below a contribution of 1.5).


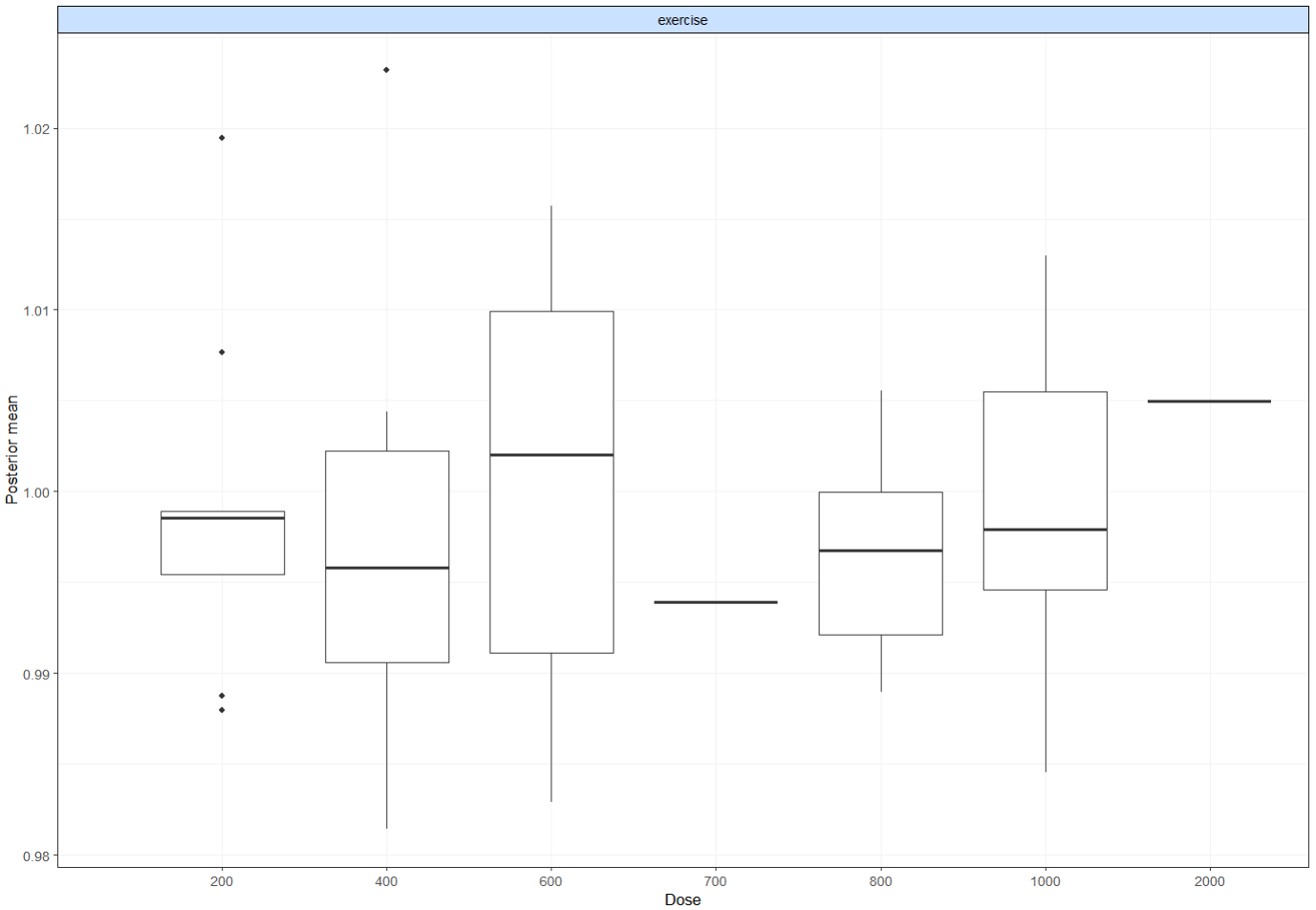


**Figure S11a**. Deviance plot at overall exercise (fatigue)


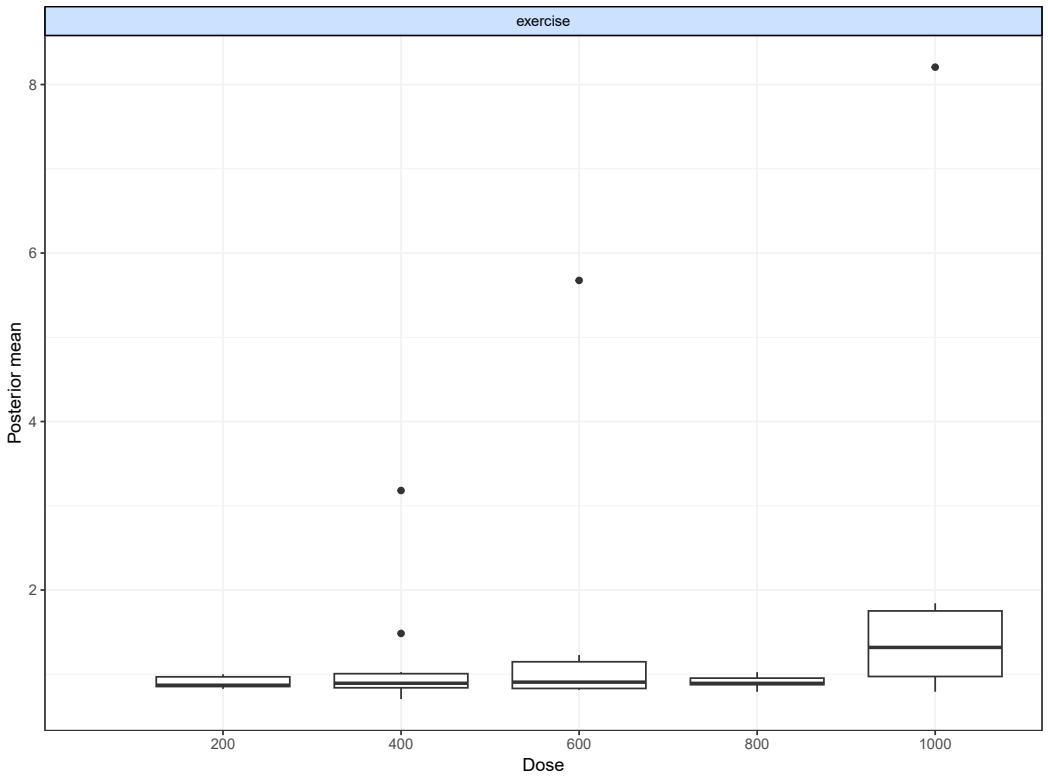


**Figure S11b**. Deviance plot at overall exercise (muscle); HIIT High-intensity interval training, COM Combined exercise, RT Resistance training, AE Aerobic exercise, MBE Mind–body exercises. CG, control group.


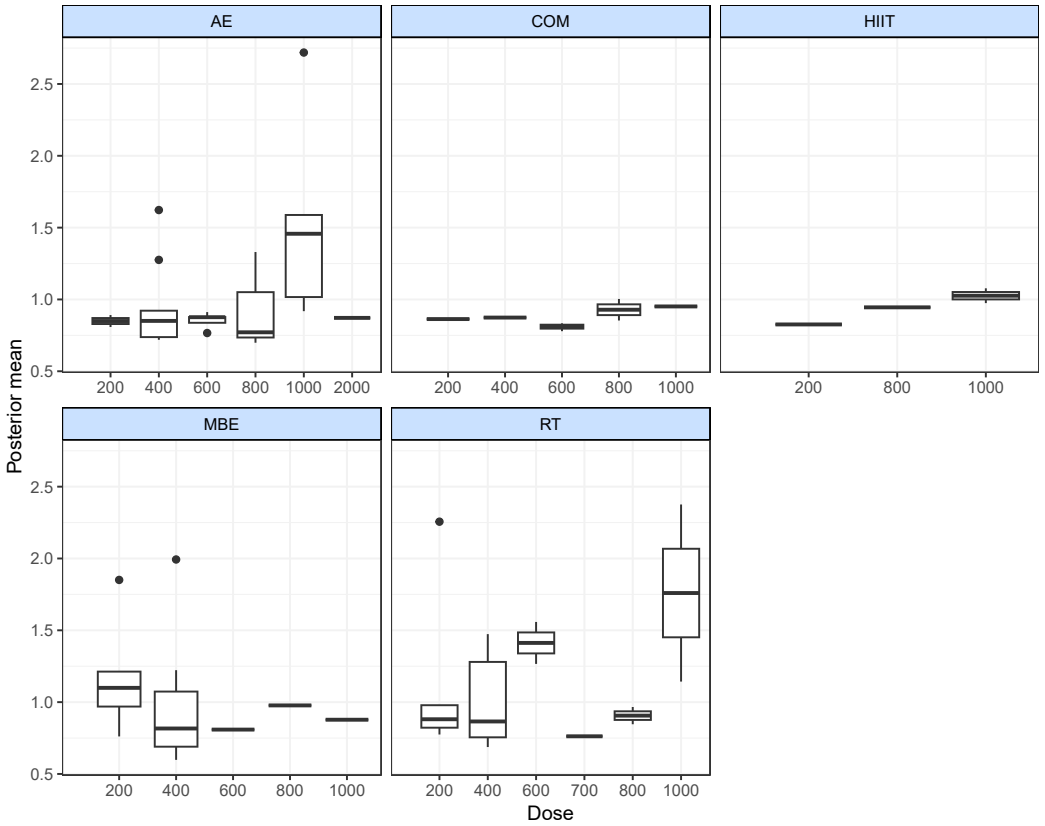


**Figure S12a.** Deviance plots at treatment-level. (fatigue); HIIT High-intensity interval training, COM Combined exercise, RT Resistance training, AE Aerobic exercise, MBE Mind–body exercises. CG, control group.


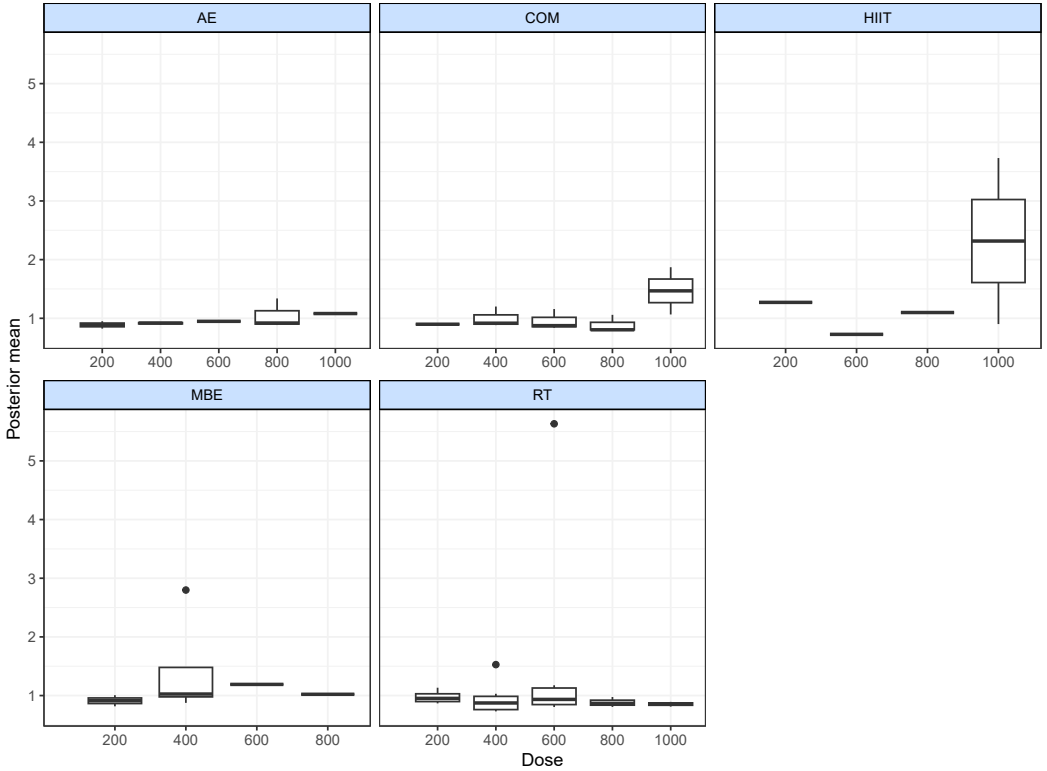


**Figure S12b.** Deviance plots at treatment-level. (muscle)

In addition, we also plotted the fit further to assess the degree of fit of the model. The fit values are plotted as connecting lines, and the observations in the original dataset are plotted as points. These plots can determine if the model fits the data well for different exercises and doses of the dose-response function.


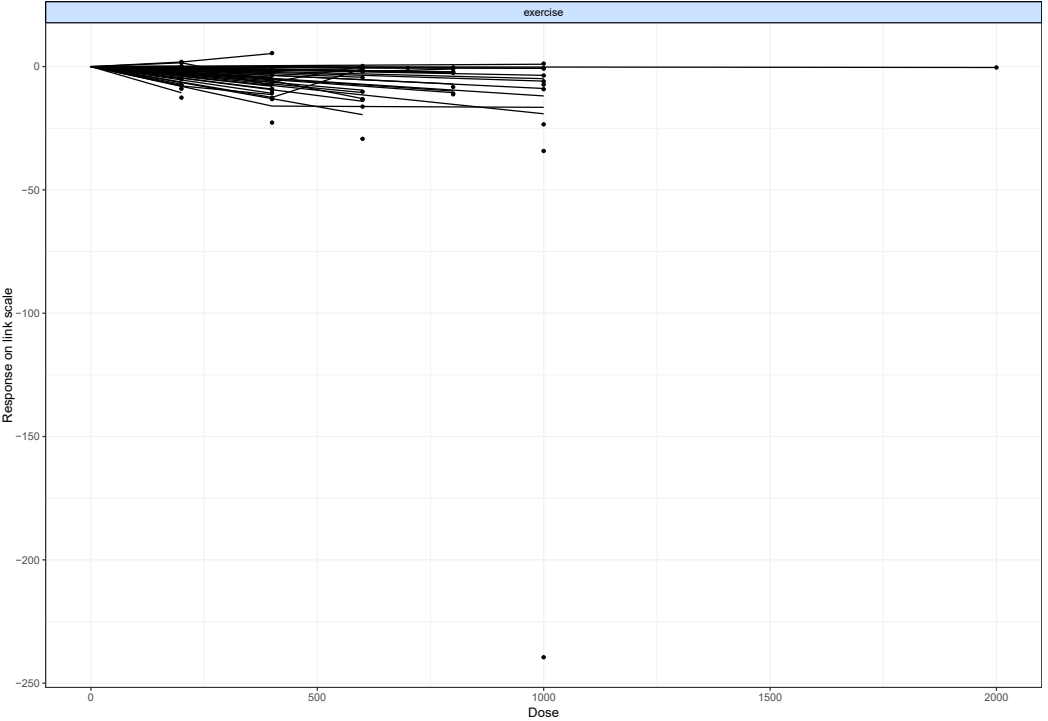


**Figure S13a.** Fit plots at overall exercise level. (fatigue)


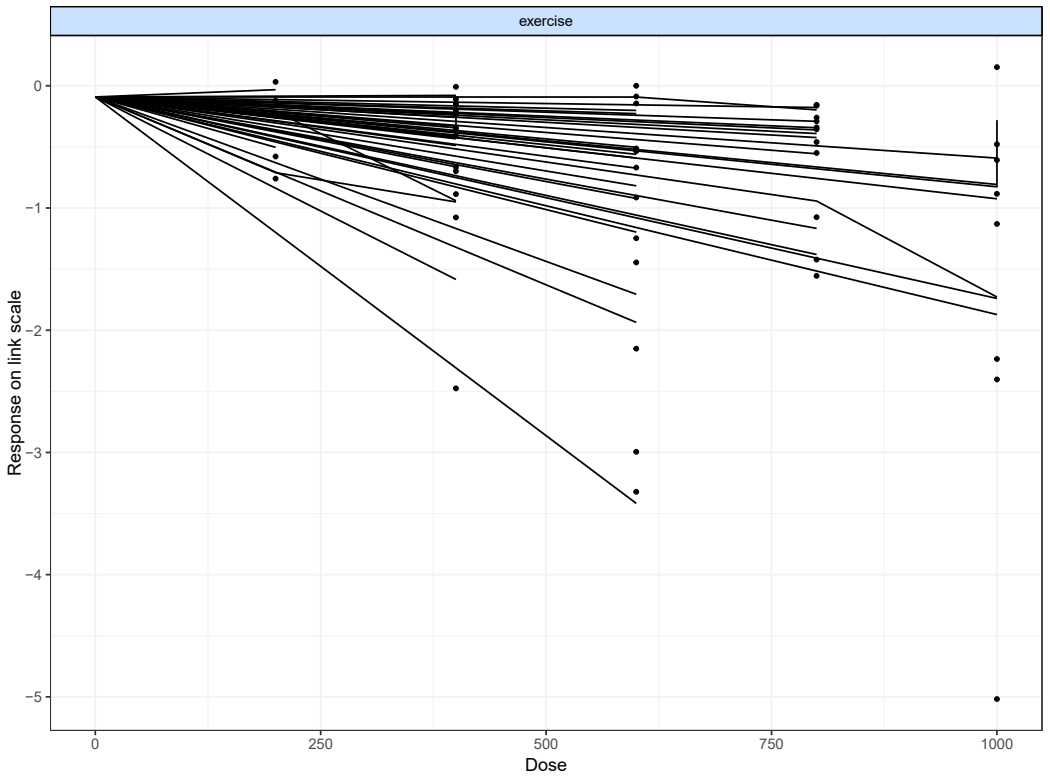


**Figure S13b.** Fit plots at overall exercise level. (muscle)


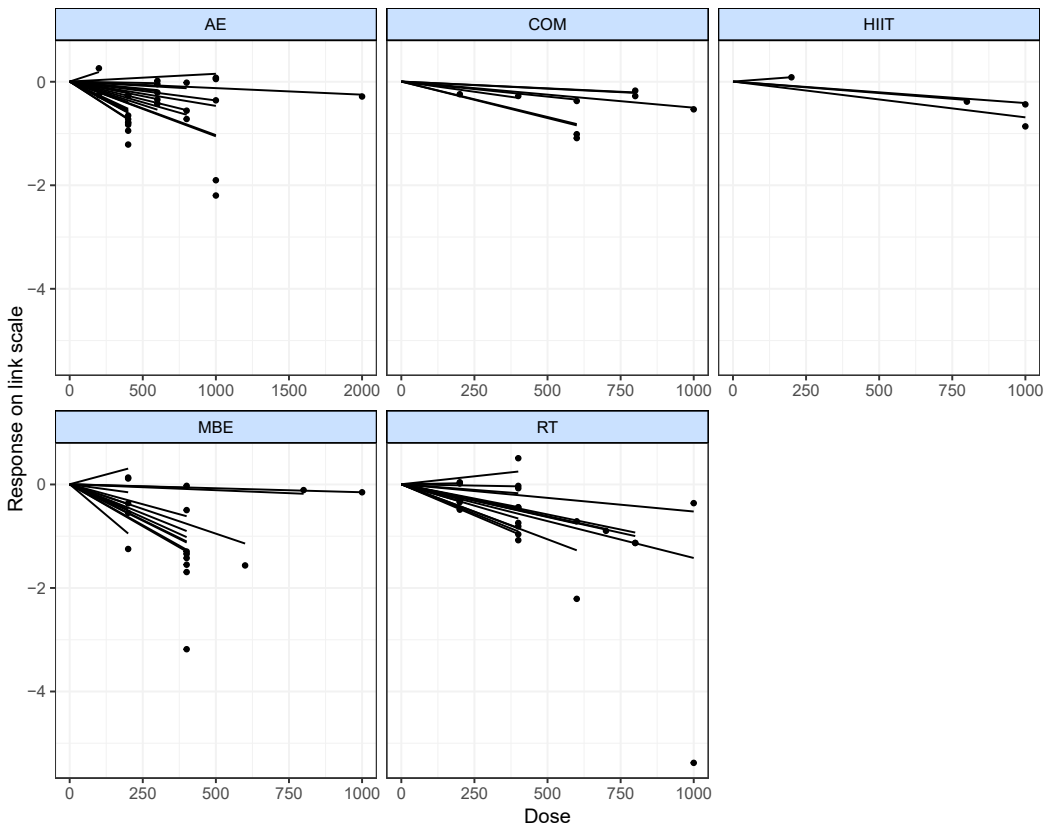


**Figure S14a.** Fit plots at agent-level. HIIT High-intensity interval training, COM Combined exercise, RT Resistance training, AE Aerobic exercise, MBE Mind–body exercises. CG, control group. (fatigue)


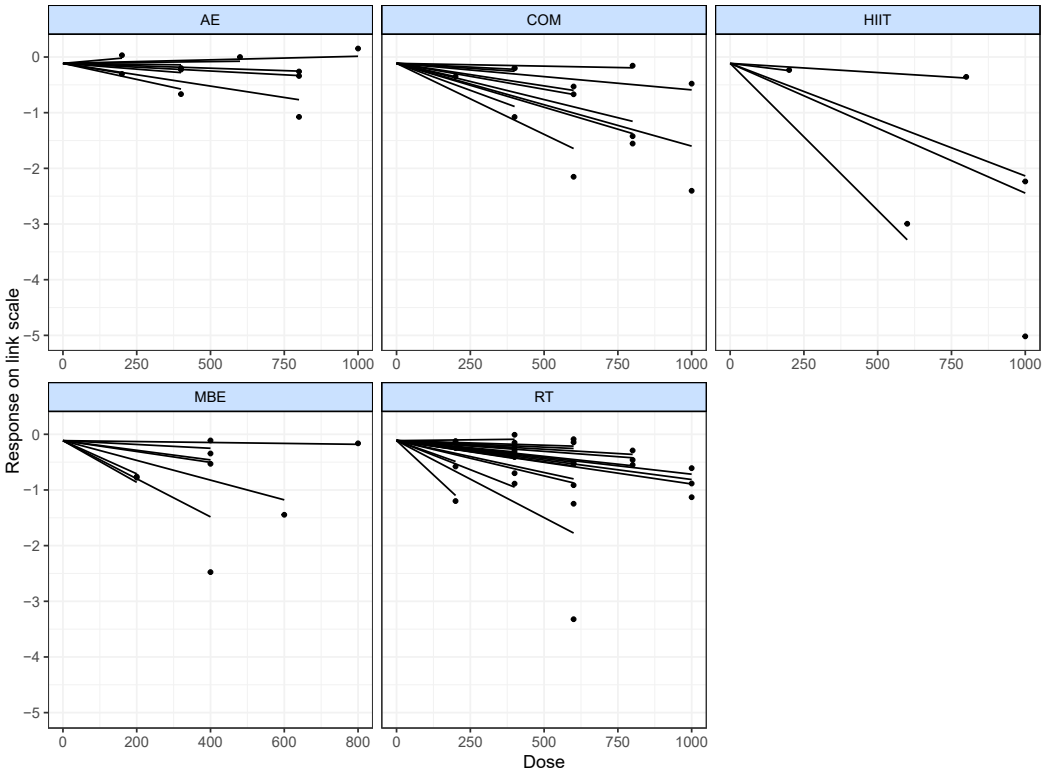


**Figure S14b.** Fit plots at agent-level. HIIT High-intensity interval training, COM Combined exercise, RT Resistance training, AE Aerobic exercise, MBE Mind–body exercises. CG, control group.. (muscle)

## 5.3 Dose-response relationships

1.
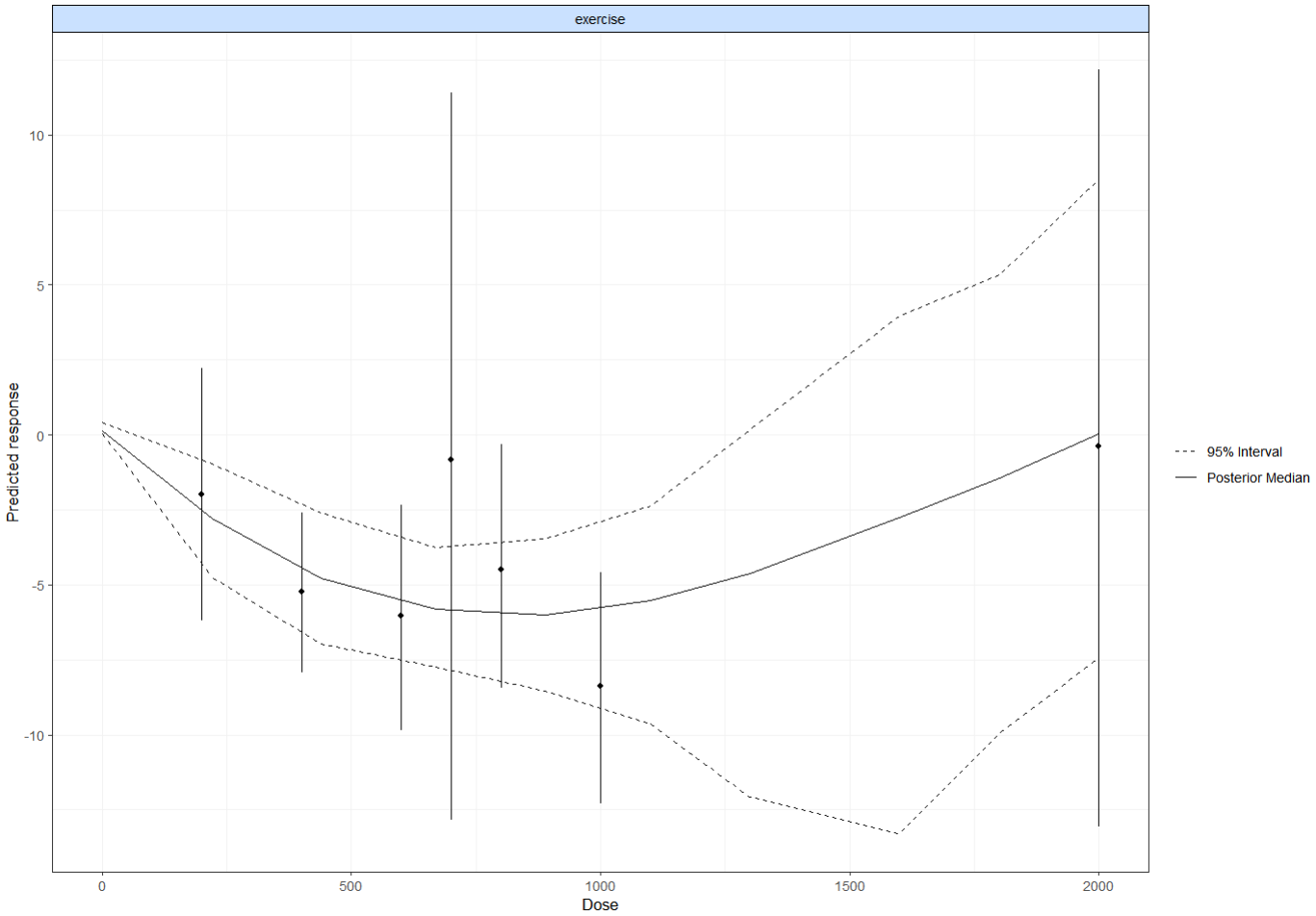


2.
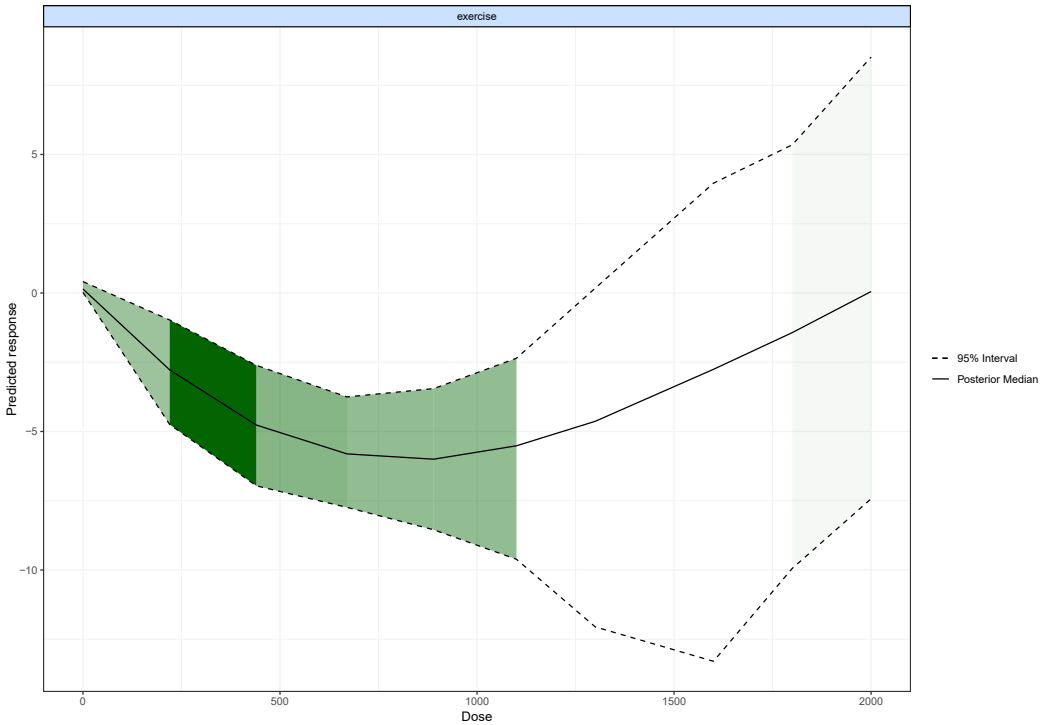


**Figure S15a.** Dose-response relationship between exercise dose and fatigue.

Figure **1** represents the dose-response + node-split, and the shaded area in Figure **2** represents the original study dataset; the darker the color, the larger the amount of data. (fatigue)

1.
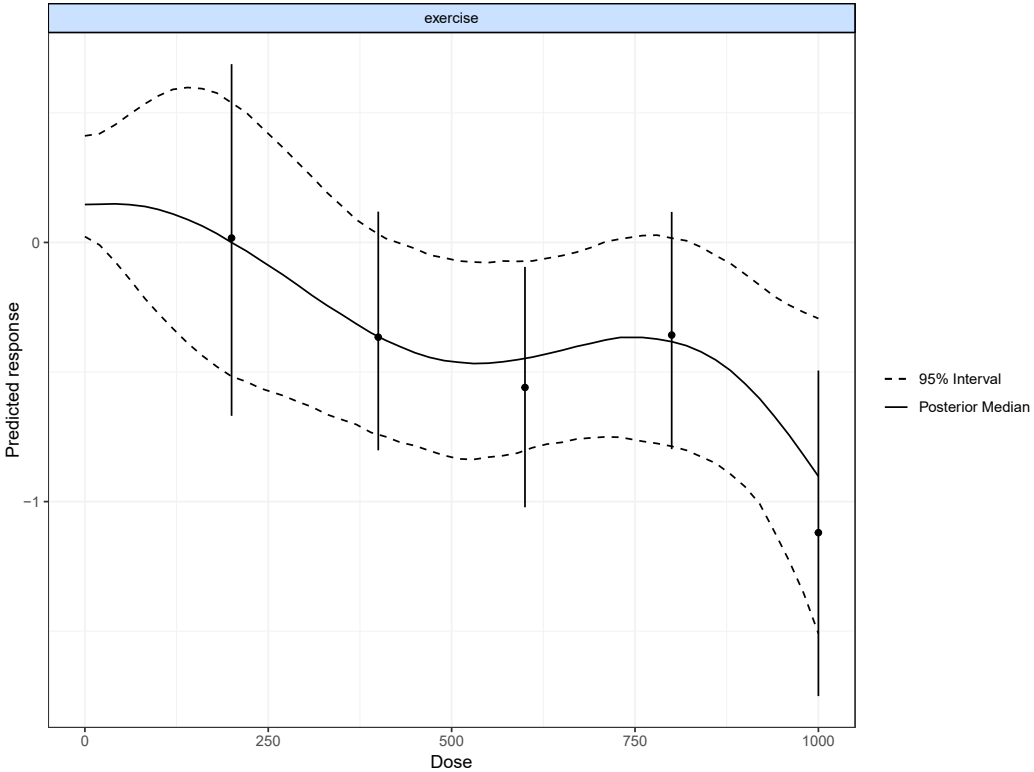


2.
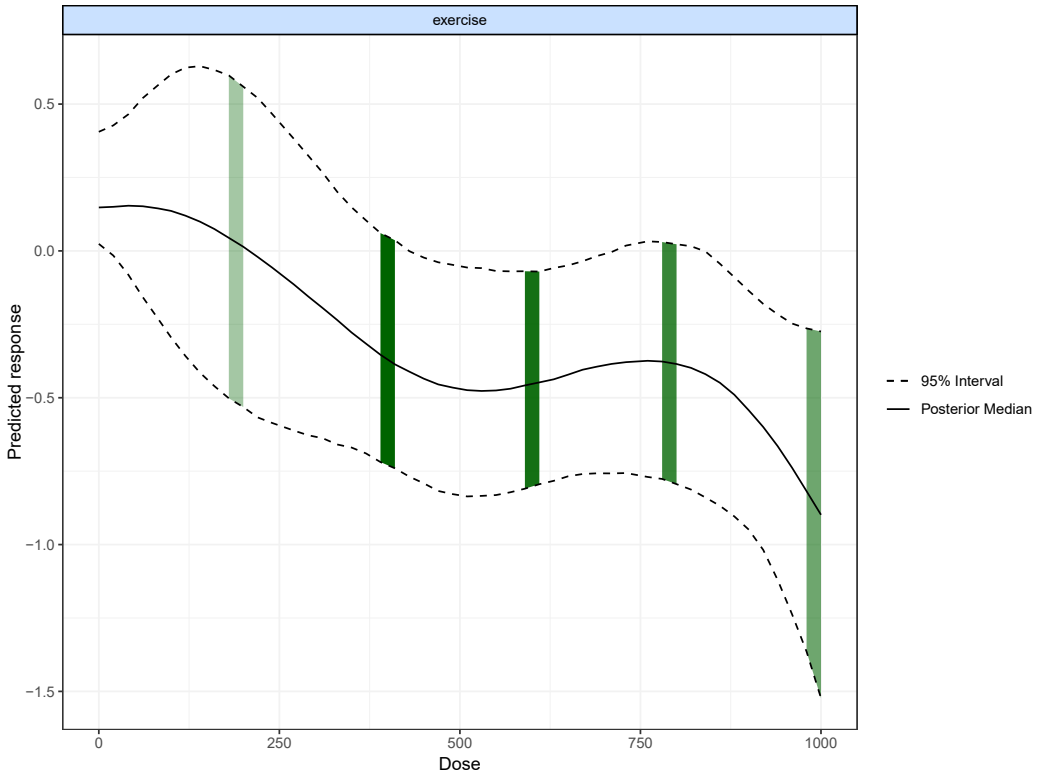


**Figure S15b.** Dose-response relationship between exercise dose and muscular fitness.

Figure **1** represents the dose-response + node-split, and the shaded area in Figure **2** represents the original study dataset; the darker the color, the larger the amount of data. (muscle)


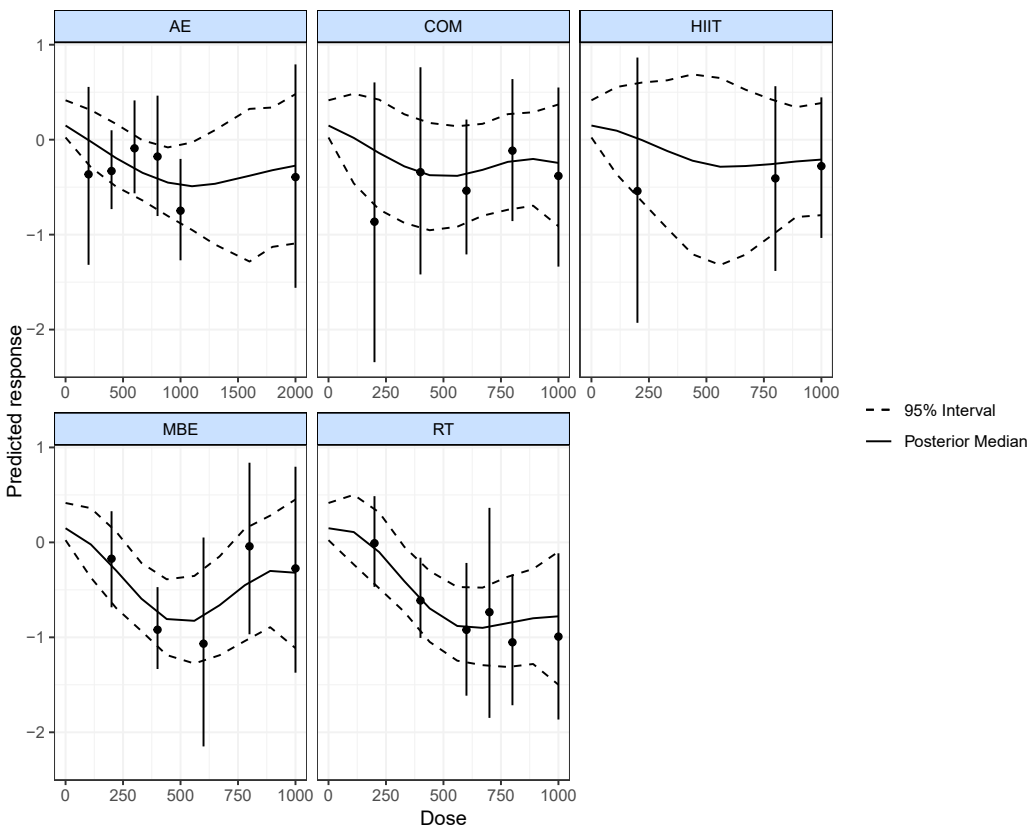


**Figure S16a.** Prediction dose-response relationship at agent-level. PI, prediction interval. (fatigue) HIIT High-intensity interval training, COM Combined exercise, RT Resistance training, AE Aerobic exercise, MBE Mind–body exercises. CG, control group.


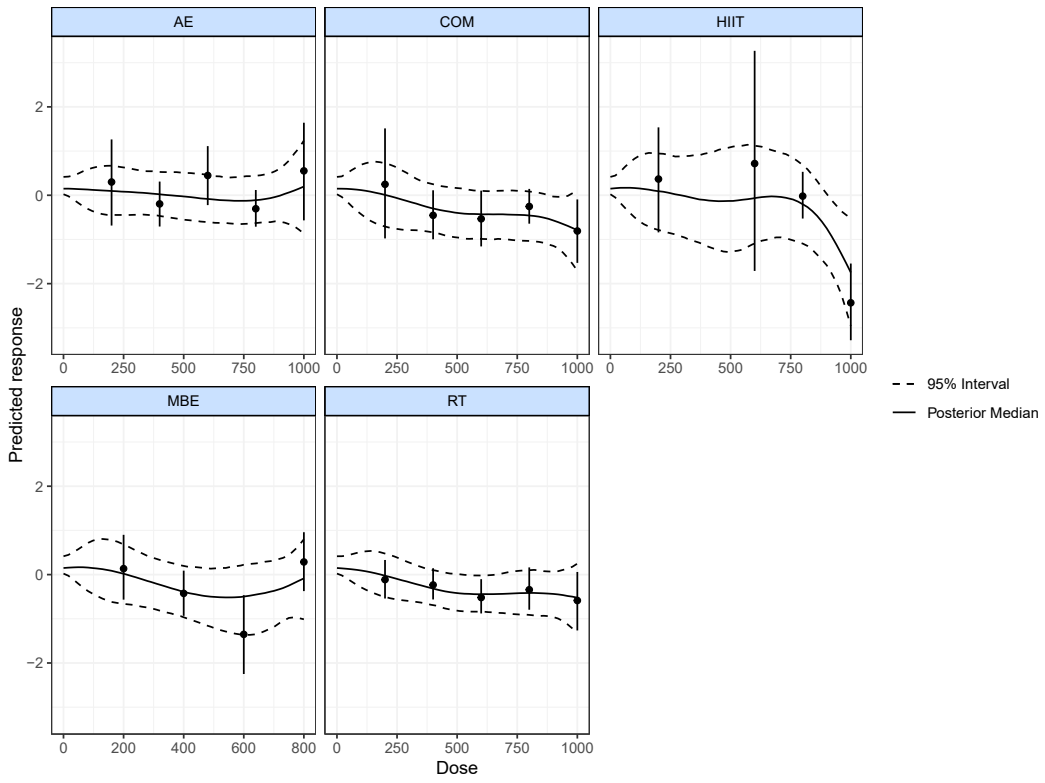


**Figure S16b.** Prediction dose-response relationship at agent-level. PI, prediction interval. (muscle) HIIT High-intensity interval training, COM Combined exercise, RT Resistance training, AE Aerobic exercise, MBE Mind–body exercises. CG, control group.

5.3.2 Ranking of effectiveness of different exercise


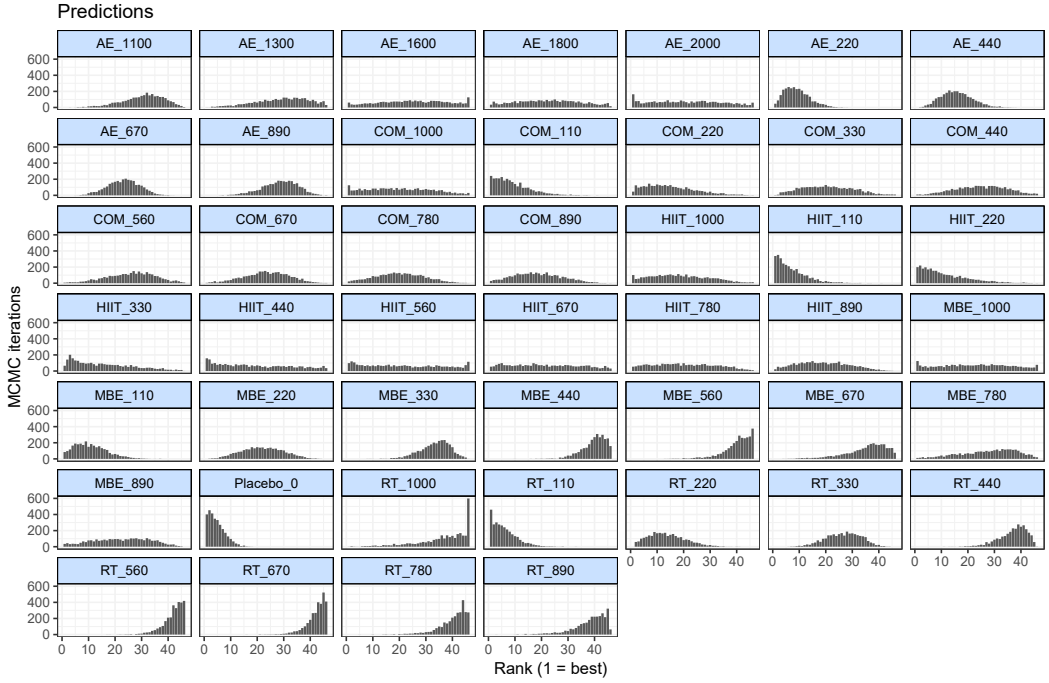


**Figure S17a** shows the probability of each intervention to be ranked from best to worst (estimated after up to 4000 iterations). (fatigue) ; HIIT High-intensity interval training, COM Combined exercise, RT Resistance training, AE Aerobic exercise, MBE Mind–body exercises. CG, control group.


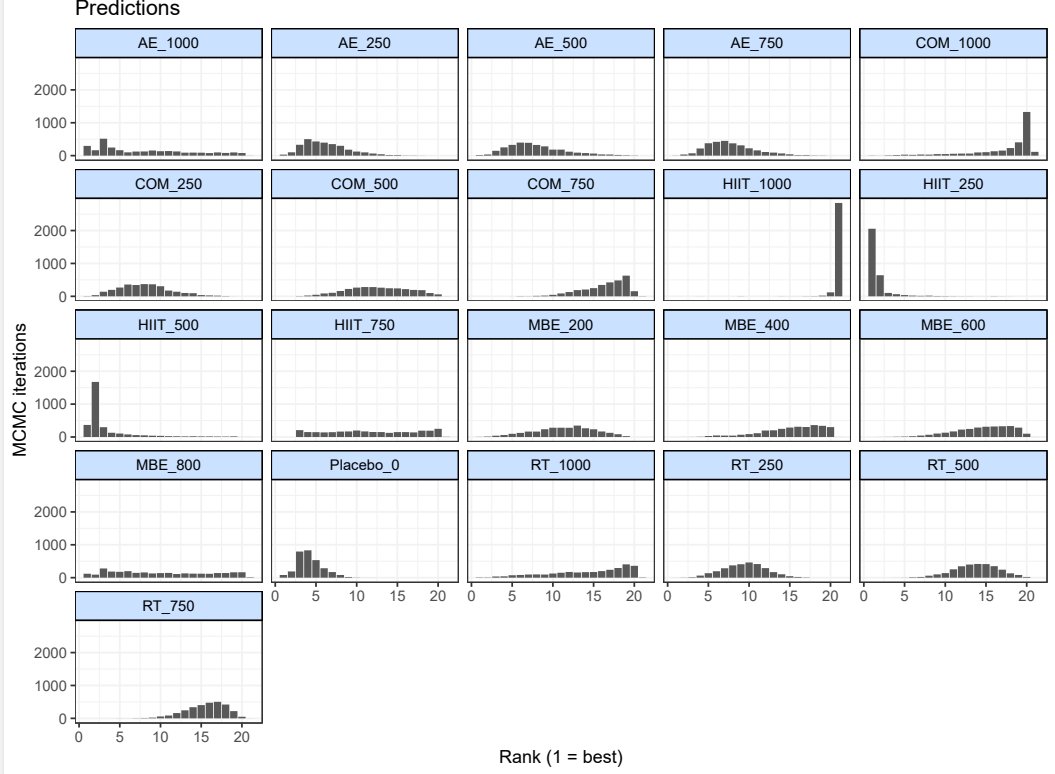


**Figure S17b** shows the probability of each intervention to be ranked from best to worst (estimated after up to 4000 iterations). (muscle) HIIT High-intensity interval training, COM Combined exercise, RT Resistance training, AE Aerobic exercise, MBE Mind–body exercises. CG, control group.

**Table S8a.** Predictions ranking (from worst to best; fatigue)


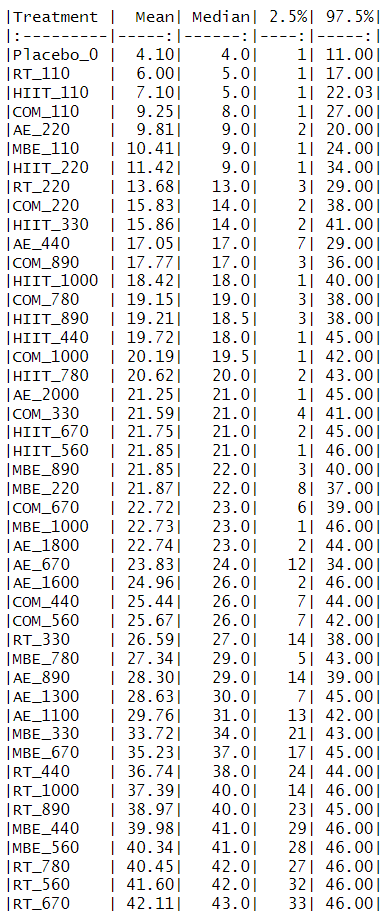


**Table S8b.** Predictions ranking (from worst to best; muscle)


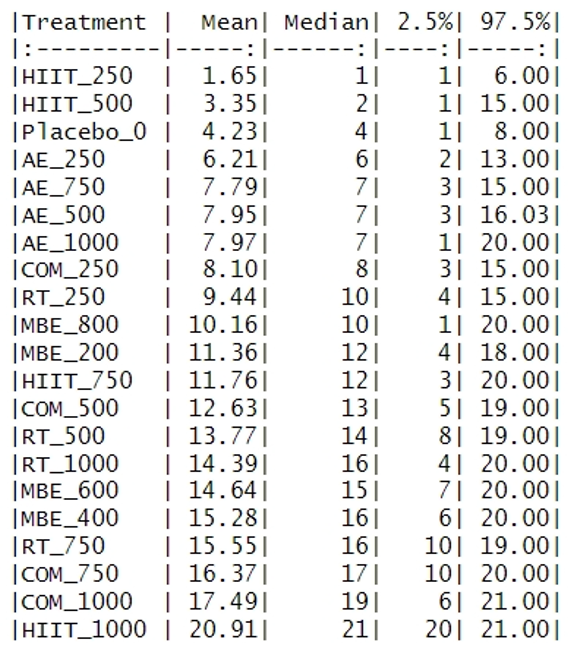


***Note:*** HIIT High-intensity interval training, COM Combined exercise, RT Resistance training, AE Aerobic exercise, MBE Mind–body exercises. CG, control group.

**6. Code in R analysis**

6.1 NMAs code

*# Load necessary packages*

packages <- c("tidyverse", "tidybayes", "brms", "readxl", "ggthemes", "ggsci",

"cowplot", "MBNMAdose", "kableExtra", "ggdist", "patchwork",

"modelr", "metR", "janitor", "marginaleffects", "metafor",

"esc", "dplyr", "openxlsx", "xlsx", "writexl", "ggplot2",

"ggridges", "glue", "stringr", "forcats")

new_packages <- packages[!(packages %in% installed.packages()[, "Package"])]

if (length(new_packages)) install.packages(new_packages)

install.packages("lme4")

.libPaths()

lapply(packages, library, character.only = TRUE)

*# Read input data*

data <- read_xlsx("R_template_data.xlsx", col_names = TRUE, na = c("NA", ""))

write_xlsx(data, "data ")

*# Load network meta-analysis package*

library(gemtc)

*# Load CSV data*

data <- read.csv("R_template_data.csv", sep = ",", header = TRUE)

*# Network data setup*

network <- mtc.network(data)

*# Network plot*

plot(network)

summary(network)

plot(network,

vertex.label.color = "blue4",

vertex.color = "green4",

dynamic.edge.width = TRUE,

edge.color = "grey")

plot(network, dynamic.edge.width = FALSE)

*# Model construction*

model <- mtc.model(network, n.chain = 4, likelihood = "normal", link = "identity", linearModel = "random")

cat(model$code)

*# Run iterations*

results <- mtc.run(model, n.adapt = 5000, n.iter = 20000, thin = 4)

summary(results)

*# UME model for inconsistency*

ume_model <- mtc.model(network, type = "ume", n.chain = 4, likelihood = "normal", link = "identity", linearModel = "random")

ume_results <- mtc.run(ume_model, n.adapt = 5000, n.iter = 20000, thin = 4)

summary(ume_results)

*# Forest plot for placebo comparison*

forest(relative.effect(results, "CG"), xlim = c(-1, 1), digits = 2)

*# Convergence diagnostics*

gelman.diag(results)

gelman.plot(results)

*# Trace and density plots*

plot(results)

*# Rank probabilities and SUCRA calculation*

rank_probs <- rank.probability(results, preferredDirection = -1)

print(rank_probs)

plot(rank_probs)

*# Node-splitting method for inconsistency check*

node_split_result <- mtc.nodesplit(network, n.adapt = 5000, n.iter = 20000, thin = 1, n.chain = 4, likelihood = "normal", link = "identity", linearModel = "random")

node_summary <- summary(node_split_result)

print(node_summary, digits = 2)

plot(node_summary)

*# Heterogeneity analysis*

heterogeneity_result <- mtc.anohe(network, n.adapt = 5000, n.iter = 20000, thin = 1, n.chain = 4, likelihood = "normal", link = "identity", linearModel = "random")

heterogeneity_summary <- summary(heterogeneity_result)

print(heterogeneity_summary)

plot(heterogeneity_summary)

*# Rank probability plot with ggplot*

rank_probs_df <- as.data.frame(melt(rank_probs))

p <- ggplot(rank_probs_df, aes(x = Var2, y = value, color = Var1)) +

geom_line(size = 1) +

geom_point(size = 2) +

labs(x = "Rank", y = "Probability") +

theme(axis.text = element_text(size = 12), axis.title = element_text(size = 15), panel.background = element_blank(), axis.line = element_line(color = "black"), legend.title = element_blank(), legend.position = "bottom")

ggsave("rank_probability_plot.pdf", p, width = 7.48, height = 6.48)

*# Cumulative rank plot*

cumulative_ranks <- t(apply(rank_probs, 1, cumsum))

cumulative_ranks_df <- as.data.frame(melt(cumulative_ranks))

p_cumulative <- ggplot(cumulative_ranks_df, aes(x = Var2, y = value, color = Var1)) +

geom_line(size = 1) +

geom_point(size = 2) +

labs(x = "Rank", y = "Cumulative Probability") +

theme(axis.text = element_text(size = 12), axis.title = element_text(size = 15), axis.line = element_line(color = "black"), legend.title = element_blank(), legend.position = "bottom")

ggsave("cumulative_rank_plot.pdf", p_cumulative, width = 7.48, height = 6.48)

*# Regression analysis*

data <- read.csv("total_age.csv", sep = ",", header = TRUE)

studies <- read.csv("age.csv", sep = ",", header = TRUE)

network <- mtc.network(data, studies = studies)

model_regression <- mtc.model(network, type = "regression", regressor = list(coefficient = 'shared', variable = 'age', control = 'CG'))

regression_results <- mtc.run(model_regression, n.adapt = 5000, n.iter = 20000, thin = 4)

summary(regression_results)

plotCovariateEffect(regression_results, t1 = 'CG', t2 = c("AE", "COM", "RT", "MBE", "HIIT"))

plotCovariateEffect(regression_results, t1 = 'CG', t2 = c('AE', 'COM', 'RT', 'MBE', 'HIIT'), ylim = c(-1.5, 1.5))

6.2 Dose-response analysis code

*# Load necessary packages*

install.packages("MBNMAdose")

install.packages("tidyverse")

install.packages("cowplot")

install.packages("janitor")

install.packages("patchwork")

install.packages("mcmcplots")

install.packages("readxl")

install.packages("rjags")

install.packages("multinma")

install.packages("metafor")

install.packages("overlapping")

install.packages("ggthemes")

install.packages("splines")

install.packages("Hmisc")

install.packages("rms")

install.packages("segmented")

install.packages("metacart")

install.packages("ggplot2")

install.packages("openxlsx")

install.packages("forestplot")

library(MBNMAdose)

library(tidyverse)

library(cowplot)

library(janitor)

library(patchwork)

library(readxl)

library(mcmcplots)

library(rjags)

library(multinma)

library(metafor)

library(overlapping)

library(ggthemes)

library(splines)

library(Hmisc)

library(rms)

library(segmented)

library(metacart)

library(ggplot2)

library(openxlsx)

library(forestplot)

options(max.print = 1000000)

*# Create network*

network <- mbnma.network(X1)

*# Treatment-level network plot*

plot(network, v.color = "agent", label.distance = 4, remove.loops = TRUE)

*# Agent-level network plot*

plot(network, v.color = "agent", level = "agent", label.distance = 3)

*# Network summary*

summary(network)

*# Model setup and UME consistency check*

nma_network <- nma.run(network, link = "smd", method = "random", likelihood = "normal")

print(nma_network)

plot(nma_network)

*# UME model*

ume_network <- mbnma.run(network, link = "smd", method = "random", UME = TRUE)

print(ume_network)

*# Dose-response plot*

plot(nma_network) +

xlab("Dose (METs-min/week)") +

ylab("Effect size (Hedges' g)") +

scale_x_continuous(breaks = c(0, 200, 400, 600, 800, 1000, 2000))

*# Emax model*

emax <- mbnma.run(network, link = "smd", fun = demax(emax = "rel", ed50 = "rel"), method = "common")

print(emax)

pred <- predict(emax, E0 = 0)

plot(pred)

*# Restricted cubic splines with knots*

knots = c(0.1, 0.5, 0.9)

rcs <- mbnma.run(network, link = "smd", fun = dspline(type = "ns", knots = knots), method = "random", likelihood = "normal")

print(rcs)

pred <- predict(rcs, E0 = "rbeta(n, shape1 = 2, shape2 = 10)", max.doses = doses, n.dose = 50, lim = "cred")

plot(pred, overlay.split = TRUE)

summary(pred)

*# Linear model (optional)*

linear <- mbnma.linear(network, slope = "rel")

print(linear)

pred <- predict(linear, E0 = 0)

plot(pred)

summary(pred)

*# Non-parametric dose-response*

non_param <- mbnma.run(network, link = "smd", fun = "nonparam.up")

print(non_param)

plot(non_param)

*# Quadratic model*

dpoly <- ~ (beta.1 * dose) + (beta.2 * (dose^2))

dpoly <- mbnma.run(network, link = "smd", fun = duser(fun = dpoly, beta.1 = "rel", beta.2 = "rel"), method = "random")

print(dpoly)

pred <- predict(dpoly, E0 = "rbeta(n, shape1 = 2, shape2 = 10)", max.doses = doses, n.dose = 50, lim = "cred")

plot(pred, overlay.split = TRUE)

summary(pred)

*# Ranking predictions*

pred <- predict(dpoly, E0 = "rbeta(n, shape1 = 2, shape2 = 10)", max.doses = doses, n.dose = 10)

rank <- rank(pred, lower_better = TRUE)

print(rank)

plot(rank)

*# Model fit assessment*

devplot(rcs, plot.type = "box")

devplot(dpoly, plot.type = "box")

fits_1 <- MBNMAdose::fitplot(dpoly)

fits_2 <- MBNMAdose::fitplot(rcs)

*# Inconsistency check*

splitcomps <- inconsistency.loops(network$data.ab, incldr = TRUE)

nodesplit <- mbnma.nodesplit(network, fun = dpoly(degree = 1), comparisons = splitcomps, link = "smd", method = "random")

print(nodesplit)

plot(nodesplit, plot.type = "density")

*# Additional dose plot*

plot(pred, disp.obs = TRUE)

**7. Reference:**

1. Dias S, Ades, A. E., Welton, N. J., Jansen, J. P. & Sutton, A. J. . Network meta-analysis for decision-making: John Wiley & Sons; 2018.

2. Dias S, Sutton AJ, Ades AE, Welton NJ. Evidence synthesis for decision making 2: a generalized linear modeling framework for pairwise and network meta-analysis of randomized controlled trials. Med Decis Making. 2013;33(5):607-17.
